# Diversity and Genetic Reassortment of Keystone Virus in Mosquito Populations in Florida

**DOI:** 10.4269/ajtmh.22-0594

**Published:** 2023-05-01

**Authors:** Maha A. Elbadry, Caroline A. Efstathion, Whitney A. Qualls, Massimiliano S. Tagliamonte, Md. Mahbubul Alam, Md. Siddiqur Rahman Khan, Sadie J. Ryan, Rui-de Xue, Remi N. Charrel, Lea Bangonan, Marco Salemi, Nazli Ayhan, John A. Lednicky, J. Glenn Morris

**Affiliations:** ^1^ Emerging Pathogens Institute, University of Florida, Gainesville, Florida;; ^2^ Volusia Mosquito Control District, New Smyrna Beach, Florida;; ^3^ Anastasia Mosquito Control District, St. Augustine, Florida;; ^4^ Department of Pathology, Immunology, and Laboratory Medicine, College of Medicine, University of Florida, Gainesville, Florida;; ^5^ Department of Environmental and Global Health, College of Public Health and Health Professions, University of Florida, Gainesville, Florida;; ^6^ Department of Geography, College of Liberal Arts and Sciences, University of Florida, Gainesville, Florida;; ^7^ Unité des Virus Emergents, Aix Marseille University, INSERM U1207, Marseille, France;; ^8^ Department of Medicine, College of Medicine, University of Florida, Gainesville, Florida

## Abstract

*Keystone orthobunyavirus* (KEYV), a member of the genus *Orthobunyavirus*, was first isolated in 1964 from mosquitoes in Keystone, Florida. Although data on human infections are limited, the virus has been linked to a fever/rash syndrome and, possibly, encephalitis, with early studies suggesting that 20% of persons in the Tampa, Florida, region had antibodies to KEYV. To assess the distribution and diversity of KEYV in other regions of Florida, we collected > 6,000 mosquitoes from 43 sampling sites in St. Johns County between June 2019 and April 2020. Mosquitoes were separated into pools by species and collection date and site. All pools with *Aedes* spp. (293 pools, 2,171 mosquitoes) were screened with a real-time reverse transcriptase polymerase chain reaction (rRT-PCR) assay that identifies KEYV and other closely related virus species of what was previously designated as the California encephalitis serogroup. In 2020, screening for KEYV was expanded to include 211 pools of *Culex* mosquitoes from sites where KEYV-positive *Aedes* spp. had been identified. rRT-PCR–positive samples were inoculated into cell cultures, and five KEYV isolates from *Aedes atlanticus* pools were isolated and sequenced. Analyses of the KEYV large genome segment sequences revealed two distinct KEYV clades, whereas analyses of the medium and small genome segments uncovered past reassortment events. Our data documented the ongoing seasonal circulation of multiple KEYV clades within *Ae. atlanticus* mosquito populations along the east coast of Florida, highlighting the need for further studies of the impact of this virus on human health.

## INTRODUCTION

Keystone virus (KEYV) is a member of the genus *Orthobunyavirus* and is the sole member of the species *Keystone orthobunyavirus*.^1^ It was first identified in 1964 in Keystone, a small town in west-central Florida near Tampa.^2^ Serologic studies after its identification suggested that approximately 20% of the population in endemic areas had antibodies to the virus^3^; there has been at least one case of meningoencephalitis in a child in which KEYV was implicated as a possible etiologic agent,^4^ and it recently was isolated from a teenager in north-central Florida with a fever/rash syndrome.^5^ KEYV was initially placed within what was designated as the California encephalitis serogroup, which included other *Orthobunyavirus* spp. that were associated with meningoencephalitis in humans, such as La Crosse virus and Jamestown Canyon virus (JCV). It is noteworthy that the illnesses they cause are currently on the U.S. CDC list of notifiable diseases. Although the California encephalitis serogroup classification scheme is no longer used, the association with other viruses linked to meningoencephalitis raises questions about whether KEYV might be an underrecognized cause of illness (including meningoencephalitis) among persons living in the southeastern United States, particularly as identification of the virus in clinical cases has not been possible outside of a research setting.

*Aedes atlanticus* has been identified as the primary vector for KEYV in prior studies, and there are data suggesting that the virus can undergo transovarian transmission in this species.^6^^–^^12^ The virus has also been associated with 10 other types of mosquitoes, including *Culex* spp., but the role of these other vector species in KEYV transmission and infection remains uncertain.^8^^,^^9^^,^^13^ Limited data are available on vertebrate hosts that sustain the virus in a sylvatic cycle, although cotton rats (*Sigmodon hispidus*) and rabbits (*Sylvilagus floridanus*) are known to be amplifying hosts; there are no data to assess the possibility of an urban transmission cycle in which humans might play a role. Despite the fact that KEYV has been recognized for more than 50 years, sequence data are available for only four KEYV genomes from outside of our group (Table 1), which has limited the ability of investigators to assess strain diversity and evolutionary trends. To better characterize the dynamics of KEYV in mosquito populations (and as an initial step in understanding the risk of KEYV infection in humans), we screened mosquito pools from St. Johns County on Florida’s east coast for KEYV by real-time reverse transcriptase polymerase chain reaction (rRT-PCR), virus isolation in cell cultures, genomic sequencing of virus isolates, and PCR of mosquito DNA for verification of mosquito species.

**Table 1:** KEYV strains for which sequence data are available

Strain	Host	Origin	Collection date	Segment L NCBI accession no.	Segment M NCBI accession no.	Segment S NCBI accession no.
AR14033	*Culex* sp.	TX	May 2016	MG821229.1	MG821230.1	MG821231.1
AVA1709441	*Culex* sp.	Orange County, TX	Aug. 2017	MG765469.1	MG765470.1	MG765471.1
B64-5587.05	*Aedes cf. atlanticus/ tormentor*	FL	1964	NC_043629.1	NC_043627.1	NC_043628.1
KEYVLK01	*Ae. atlanticus*	Sarasota County, Fl	June 2005	KT630288.1	KT630289.1	KT630290.1
KEYVLK02	*Ae. atlanticus*	Sarasota County, FL	June 2005	KT630291.1	KT630292.1	KT630293.1
Gainesville-1/2016	*Homo sapiens*	Alachua County, FL	Aug. 2016	MH016784.**1**	MH016785.**1**	MH016786.**1**
St. Johns County-FL-1/2019	*Ae. atlanticus*	St. Johns County, FL	July 2019	MT127621.1	MT127622.1	MT127623.1
St. Johns County-FL-2/2019	*Ae. atlanticus*	St. Johns County, FL	Oct. 2019	MZ156786.1	MZ156785.1	MZ156784.1
St. Johns County-FL-3/2019	*Ae. atlanticus*	St. Johns County, FL	Oct. 2019	MZ156789.1	MZ156788.1	MZ156787.1
St. Johns County-FL-4/2019	*Ae. atlanticus*	St. Johns County, FL	Oct. 2019	MZ156792.1	MZ156791.1	MZ156790.1
St. Johns County-FL-5/2019	*Ae. atlanticus*	St. Johns County, FL	Oct. 2019	MZ156795.1	MZ156794.1	MZ156793.1

KEYV = *Keystone orthobunyavirus*; NCBI = National Center for Biotechnology Information; no. = number.

## MATERIALS AND METHODS

### Sampling sites and strategy.

The Anastasia Mosquito Control District (AMCD) is responsible for mosquito surveillance and control in St. Johns County, Florida, which includes the city of St. Augustine. Routine surveillance is conducted by using CDC light traps (John W. Hock, Gainesville, FL) baited with CO_2_ and placed along with BG-Sentinel traps (BioGents, Regensburg, Germany), baited with BG lure, for mosquito capture. For the current study, mosquitoes in traps were anaesthetized via exposure to CO_2_ gas until no movement was observed. Taxonomists at AMCD identified mosquitoes to the species level by using taxonomic keys,^14^ and mosquitoes were then separated into pools by species, sex, date of collection, and trap type and location. For the current study, mosquito pools were stored at −80°C pending transfer to the arbovirus research laboratory at UF/EPI for further analyses. To confirm mosquito species identification in a subset of *Ae. atlanticus* mosquito pools, PCR was used to amplify ∼710 bp of the mitochondrial cytochrome C oxidase subunit I (COI) gene from mosquito pool samples. The amplified segments were then sequenced, and the results subjected to nucleotide Basic Local Alignment Search Tool (National Center for Biotechnology Information) analyses to confirm identity as previously described at Aix Marseille Université in France.^15^^,^^16^

**Figure 1.**
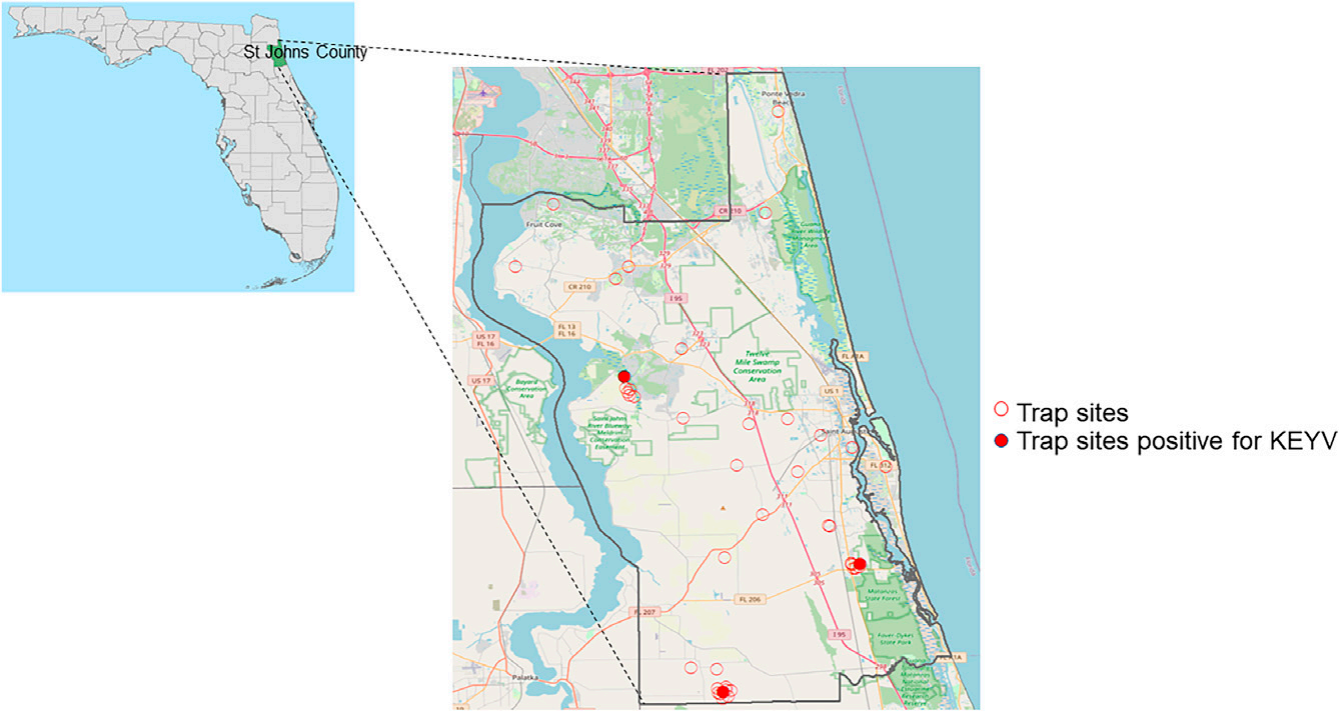
Map of sampling locations in St. Johns County. Map showing St. Johns County within Florida (left), and mosquito trapping sites in St. Johns County (red circles), showing the three trapping sites (filled red circles) for which mosquito pools were found positive for *Keystone orthobunyavirus* (KEYV) (right). County boundaries derived from the U.S. Census Bureau: American Community Survey 2010, from the Florida Geographic Data Library (FGDL, fgdl.org, accessed June 6, 2022). Basemap data for St. Johns county were obtained from Open Street Map (OSM), © OpenStreetMap (and) contributors, CC-BY-SA (openstreetmap.org).

Samples were collected from 43 sites in St. Johns County (Figure 1). Mosquito pools that contained *Ae. atlanticus*, *Aedes albopictus*, and *Aedes infirmatus* were screened using a multiplex RT-PCR for KEYV and Melao virus (MELV) as described subsequently. In 2020, additional sampling sites were selected around each of the positive sites from 2019, and screening at these sites was expanded to include collection and screening of mosquito pools containing the most common *Culex* species in northeast Florida (*Cx. coronator*, *Cx. melanoconian*, *Cx. quinquefasciatus* and *Cx. salinarius*).

### Real-time RT-PCR.

A novel KEYV/MELV primer and probe mix was designed at Aix Marseille Université in France to detect the genomic RNA of *Orthobunyavirus* spp. KEYV and MELV,^17^ which are closely related genetically, as well as other viruses, such as Serrado Navio, Inkoo, Jameston Canyon, Jerry Slough, and South River. Alignments are presented as supplemental material (Supplemental Document 1). Sequences of the primers and probes are presented in Table 2; the probes were 5′ labeled with the same dye (6-FAM). A synthetic standard RNA was used for assessing analytical sensitivity. The target regions, included in a plasmid synthetized by Genscript (GenScript, Piscataway, NJ), were amplified by PCR. The RNA transcript was synthetized in vitro using the MEGAshortscript^TM^ T7 Transcription Kit (Invitrogen-Thermo Fisher Scientific^TM^, Waltham, MA), and the RNA concentration was determined using a Thermo Scientific NanoDrop^TM^ (Thermo Fisher Scientific). The RNA transcript was serially diluted from 10^8^ to 10^2^ copies/µL, and dilutions were stored at –80°C. Limit of detection (LoD95) of the lyophilized reagents was calculated as previously described.^18^^–^^20^ Briefly, the evaluation of the sensitivity was done by using 14 serial dilutions of the quantified in vitro transcribed RNA containing 3.45 × 10^3^ to 4.21 × 10^1^ RNA copies/µL tested using 12 replicates for each. A Ct value > 40 was considered negative. The lower LoD was determined by probit regression analysis, using IBM SPSS Statistics software version 24. The LoD95 was defined as a concentration of viral copies, achieving a 95% hit rate. LoD95 was 104.93 (95% CI: 62.97–257.17; Supplemental Document 2).

**Table 2:** KEYV small genome rRT-PCR primers and probes

Primer probe name	Sequence	Conc in Rx
MELcxS35-56 (F)	CAGGTGCAAATGGATTTGATCC	400 nM
MELcxP113-137 (P)	CCGTTAGGATCTTYTTCCTTAATGC	160 nM
MELCXR164-145 (R)	CGAGHGAGAGCAGYTTTGGC	400 nM
MELcxS193-212 (F)	TTTGGAGASTGGCAGGTGGA	400 nM
MELCXP218-224 (P)	TCAAYAATCATTTTCCTGGRAACAGGA	160 nM
MELCXR273-249 (R)	TRAGATCGTTGTTACCAATTGGG	400 nM

Conc in Rx = concentration in reaction; F = forward primer; KEYV = *Keystone orthobunyavirus*; P = probe; R = reverse primer; rRT-PCR = real-time reverse transcriptase polymerase chain reaction.

Mosquito pools containing no more than 25 mosquitoes per pool were homogenized in chilled phosphate-buffered saline (PBS) and a mixture of 2 mm and 0.1 mm high-density yttrium-zirconium beads (Glen Mills, Clifton, NJ) that had been chilled at –20°C.^21^ Briefly, homogenates were prepared by bead-beading mosquitoes in 750 µL of PBS (Gibco, Thermo Fisher Scientific, Waltham, MA) in a Bead-Bug (Benchmark Scientific Sayerville, NJ) at 3,000 vibrations/minute for 90seconds, and the tubes were immediately moved to a chilled rack. The tubes were then centrifuged at 8,000 rpm for 1 minute to pellet the mosquito debris, and 140 µL of the supernatant was immediately added to 560 µL of Qiagen QIAamp virus RNA Extraction Buffer (Valencia, CA) for RNA extraction using the QIAamp viral RNA extraction kit according to the manufacturer’s instructions. The remaining ∼500 µL of the supernatant was transferred into sterile tubes containing 500 µL filtered 20% w/v trehalose in PBS to attain a final concentration of 10% (w/v) trehalose and stored immediately at –80°C until further analyses.

Real-time reverse transcriptase polymerase chain reaction was carried out using Super Script^TM^ III One Step RT-PCR with Platinum Taq polymerase (Thermo Fisher Scientific) as follows: 15 minutes at 50°C for the RT step, 2 minutes for Taq activation at 95°C, then 45 cycles of 15 seconds for denaturing at 95°C followed by 45 seconds for annealing and extension at 60°C.

### Cell lines.

Two cell lines that are susceptible and permissive for KEYV were obtained from the American Type Culture Collection (ATCC, Manassas, VA): C6/36 (*Aedes albopictus* (mosquito), ATCC CRL1660) and Vero E6 (*Cercopithecus aethiops* (African green monkey) kidney, ATCC CRL 1586). Both cell lines were grown as monolayers in a humidified atmosphere containing 5% CO_2_, the C6/36 cells at 28°C and Vero E6 cells at 37°C as described by Ahasan et al.^22^

### Virus isolation.

Mosquito pools with rRT-PCR Cq values < 38 were considered positive, and virus isolation was attempted in C6/36 and Vero E6 cells. In brief, 100-uL aliquots of unfiltered mosquito-pool supernatant were inoculated onto 2.8 × 10^6^ C6/36 cells and nearly confluent monolayers of Vero E6 cells in filter-cap T-25 flasks (Corning, Corning, NY), which were then incubated in 28°C and 37°C incubators, respectively, in the presence of 5% CO_2_. The cells were refed with cell culture maintenance media consisting of advanced Dulbecco’s modified Eagle medium (aDMEM) supplemented with gamma-irradiated, low-antibody, heat inactivated, 3% fetal bovine serum (HyClone, Logan, UT), penicillin-streptomycin-neomycin (Gibco, Thermo Fisher Scientific), and Glutamax-1 supplement (Gibco; at a final concentration of 3 mM). The cell maintenance media was changed every 3 days. The infected Vero E6 cells were observed for 14 days for any visible virus-induced cytopathic effects (CPE). The cell maintenance media of the C6/36 cell lines was blindly tested by rRT-PCR once every 3 days for virus because KEYV CPE may be difficult to detect in those cells. Vero-E6 cells that displayed CPE were scraped off the growing surface of the flasks and the cells and the maintenance media were tested by rRT-PCR for KEYV (or a closely related member of the California encephalitis serogroup viruses).

### Next-generation sequencing of virus genomes.

Next-generation sequencing was attempted on virus RNA purified from cell culture media wherein a Cq value of < 20 was attained in rRT-PCR tests. cDNA libraries were prepared using the NEB Ultra II RNAseq Library Prep Kit (New England Biolabs, Ipswich, MA), and sequencing was performed using a version 3 chemistry 600-cycle kit that was run on a MiSeq sequencer (Illumina, San Diego, CA). Host (i.e., African Green Monkey for Vero E6 cells) reads were removed using Kraken v2.0. De novo assembly was performed in CLC Genomics workbench v.10.

### Phylogenetic analyses.

Whole genome sequence alignments were performed with MAFFT^23^ and manually refined on AliView.^24^ Recombination and reassortment analyses were performed with an algorithm based on the PHI test^25^^,^^26^ implemented SplitsTree5^27^ and with RDP4.^28^ Presence of phylogenetic signal was verified by Likelihood mapping,^29^ as implemented in IQTREE v.2.0.6.^30^ Maximum likelihood trees were calculated with the same IQTREE version, with the best fitting nucleotide substitution model according to the Bayesian information criterion and 1,000 bootstrap replicates. Nucleotide and amino acid p-distances were calculated with Mega-X v.10.0.3.^31^ Trees were visualized and exported as image using FigTree v.1.4.4.^32^

### Plaque assay.

Plaque assays were performed in Vero E6 cells using a standard agarose overlay method as described by Hamilton et al.^33^ Briefly, newly confluent Vero E6 cells grown in six-well cell culture plates were inoculated with 0.2 mL of virus serially diluted in aDMEM. The virus was adsorbed to the cells for 1 hour at 37°C with manual rocking of the plates performed every 15 minutes. After virus adsorption, the cells were washed with aDMEM and the wells overlaid with 3 mL/well of primary overlay consisting of 1.6% w/v agarose (Invitrogen) mixed 1:1 with 2× complete Eagles’ modified essential medium (EMEM; Lonza, Walkersville, MD) containing 20% fetal bovine serum and antibiotics. The plates were inverted and incubated for 3 days at 37°C, then overlaid with 2 mL of secondary overlay of 1.6% w/v agarose mixed 1:1 with 2× EMEM containing serum, antibiotics, and 0.14 mg/ml neutral red (catalog no. N2889; Sigma-Aldrich, St. Louis, MO), the plates inverted, and incubated for 2 additional days to visualize plaques.

## RESULTS

As shown in Table 3, 293 mosquito pools (total of 2,171 mosquitoes) met our criteria for screening by the KEYV/MELV rRT-PCR. Criteria included being in a pool with *Ae. atlanticus*, *Ae. infirmatus*, or *Ae. albopictus* or in a *Culex* pool collected in a location where rRT-PCR-positive pools had been identified in 2019. Of the 98 *Ae. atlanticus* pools tested, 10 were positive by the KEYV/MELV rRT-PCR test. Multiple positive pools were collected at three sites (three to four positive pools/site), as highlighted in Figure 1; all positive pools were collected in Fall 2019. Because of uncertainties about differentiation of *Ae. atlanticus* and *Ae. informatus* in four of the 10 KEYV/MELV rRT-PCR positive pools, COI amplicons were sequenced to confirm mosquito identity; in all instances, the sequenced COI amplicons had 99% identity with that of *Ae. atlanticus* (GenBank JX259518.1) with no evidence of similarity with *Ae. infirmatus*. Of the 211 *Culex *spp. pools tested, 10 pools containing either *Cx. coronator* or *Cx. salinarius* were positive with the KEYV/MELV rRT-PCR.

**Table 3:** Number of mosquitoes/mosquito pools tested with KEYV/MELV rRT-PCR, by species and year, June 2019–April 2020

Species	2019			2020		
	Total no. of tested mosquitoes	No. of tested pools	No. of positive pools	Total no. of tested mosquitoes	No. of tested pools	No. of positive pools
*Aedes albopictus*	569	62	0	0	0	0
*Aedes atlanticus*	518	68	10	49	30	0
*Aedes infirmatus*	129	40	0	691	65	0
*Aedes* spp (unidentified)	166	28	0	0	0	0
*Culex coronator*	0	0	0	49	19	9
*Culex salinarius*	0	0	0	344	87	1
*Culex quinquefasciatus*	0	0	0	35	20	0
*Culex melanoconion*	0	0	0	231	53	0

KEYV = *Keystone orthobunyavirus*; MELV = Melao virus; No. = number; rRT-PCR = real-time reverse transcriptase polymerase chain reaction.

Homogenates from two of the 10 KEYV/MELV rRT-PCR-positive pools of *Ae. atlanticus* induced CPE after inoculation onto Vero-E6 cells but did not produce readily discernable CPE in C6/36 cells. A nearly complete genome sequence was obtained for all three KEYV genome segments from spent culture medium from one of these two rRT-PCR-positive mosquito pools (virus isolate: St. Johns County-FL-1/2019; details and accession numbers are provided in Table 1). When sequencing was attempted on spent cell culture medium from the second Vero E6 cell culture showing CPE, there were unreadable sections across the L, M, and S genome segments, consistent with the occurrence of nucleotide polymorphisms indicating the presence of more than one virus variant in the sample. In a plaque assay^33^ of the second KEYV positive batch, five well-separated plaques were picked and inoculated onto fresh Vero E6 and C6/36 cells. Four of the resulting virus isolates were successfully resequenced by next-generation sequencing, revealing the presence of three distinct KEYV variants: St. Johns County-FL-3/2019; St. Johns County-FL-4/2019; and isolates St. Johns County-FL-2/2019 and St. Johns County-FL-5/2019, which were identical. No virus was detected in the other plaque from Vero E6 cells.

A third KEYV/MELV rRT-PCR-positive mosquito pool of *Ae. atlanticus* induced CPE in C6/36 cells but not in Vero-E6 cells. On sequence analysis of C6/36 culture media Long Pine Key virus, a flavivirus, was identified; the sequence of this isolate has subsequently been posted in GenBank (MZ090957.1). It is unclear whether this flavivirus generated a positive rRT-PCR signal using the KEYV primer detection assay or if there was a yet unidentified orthobunyavirus that may have been present in the mosquito pool but was not recovered in cell culture.

None of the 10 KEYV/MELV rRT-PCR-positive pools from *Culex* spp. produced CPE in cell culture, and efforts at virus isolation were unsuccessful. For rRT-PCR-positive pools from *Culex* spp. and *Ae*. *atlanticus* from which virus isolation was not possible, samples had an average Cq value of 32.

### Phylogenetic analyses.

**Figure 2.**
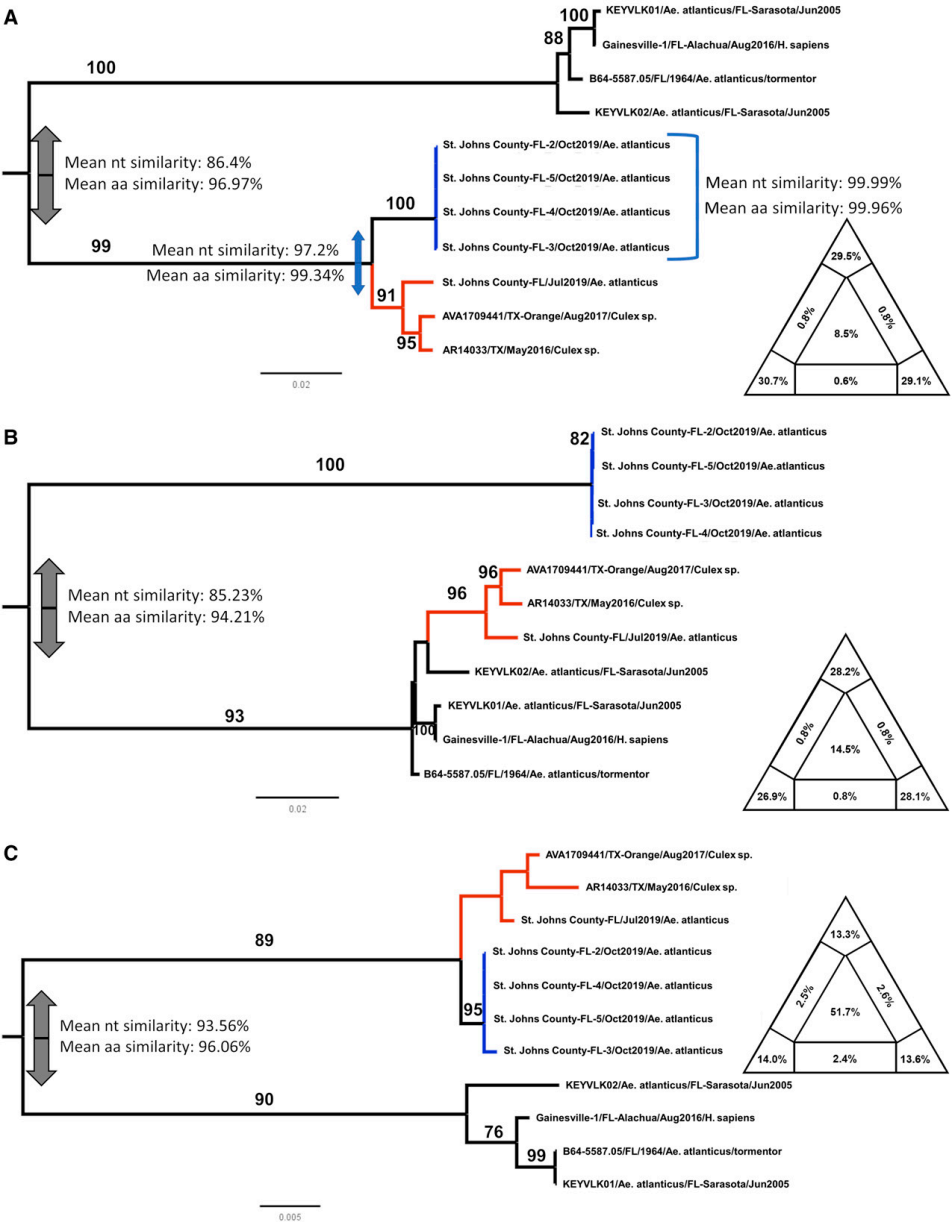
Maximum likelihood (ML) trees of *Keystone orthobunyavirus* (KEYV) strains. ML trees were inferred from 11 genome sequences, using the best fitting nucleotide substitution models as detected by Bayesian information criterion. Branches are scaled in number of nucleotide substitutions per site according to the bar below each tree. Nonparametric bootstrap values (1,000 replicates) are indicated along supported branches. The two clades hosting St. Johns county isolates are colored in red and blue. On the side of each tree, likelihood mapping shows the amount of phylogenetic signal in each alignment; details on this analysis are given in the methods section. Briefly, the higher the sum of the percentages in the triangle corners (completely resolved quartets), the higher is the phylogenetic signal contained in the alignment. The alignment used for the S segment in panel (**C**) has low phylogenetic signal (resolved quartets < 60%), and therefore caution must be used in drawing conclusions on the base of this tree results; still, the segment S tree topology matches the one for segment L, which has strong phylogenetic signal and bootstrap support. The tree inferred from segment M has a different topology from the other segments, a clear indication that genome rearrangement has occurred in the ancestry of the sequences. (**A**) Segment L ML tree; (**B**) segment M ML tree; and (**C**) segment S ML tree.

Three separate alignments were generated, one for each genomic segment (designated as L, M, and S), using all available KEYV sequences from the National Center for Biotechnology Information (NCBI) database and sequence data from the current study (Table 1). The likelihood mapping analyses showing the segment L and M alignments have a strong phylogenetic signal (Figure 2A and B) and therefore could be used for phylogenetic inference; in contrast, the segment S alignment has a much weaker signal (Figure 2C). No evidence of within-segment recombination was found in any of the segments.

Maximum likelihood (ML) trees, calculated with IQTREE v.2.0.6 (36), showed different phylogenetic histories, depending on the segment. An ML tree from segment L (Figure 2A) showed the St. Johns isolates, all obtained from *Ae. atlanticus* mosquitoes, clustering with KEYV sequences from *Culex* spp. from Texas. The other Florida KEYV sequences were derived from *Ae. atlanticus* and a human, respectively in Sarasota and Alachua counties, and clustered with the reference strain originally isolated in 1964. These two clades shared 86.4% nucleotide identity, and 96.9% amino acid identity in segment L. The segment M tree (Figure 2B) showed the St. Johns isolates from October 2019 grouped separately from the July isolate and all other sequences. Segment S alignment, although afflicted with low phylogenetic signal, matched segment L tree topology. The trees show two major clades, with high bootstrap support and high divergence.

**Figure 3.**
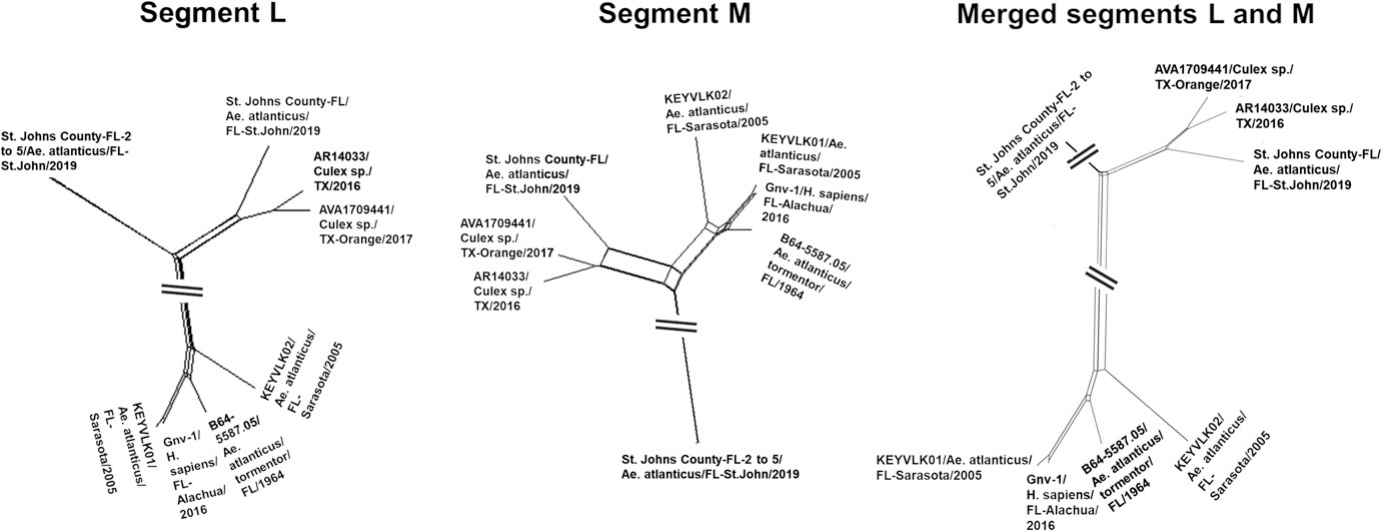
Neighbor net plots of segments L and M and merged sequences. Neighbor net was inferred from pair-wise p-distances of the 11 genomes, for segments L and M, separately, and then from the alignment resulting from merging the two fragments for each genome. Within-segment recombination for L and M, and rearrangement for the merged sequence was assessed by the PHI test. No significant evidence of recombination was found in either segment (PHI test *P* > 0.001), while a significant *P* value was found for the merged alignment (*P* < 10^−6^).

From the tree topologies, it appears that these viruses underwent reassortment events. To explore this hypothesis, we concatenated the L and M segments; we then applied algorithms that are normally used to test for recombination to assess such pseudo-sequences. At a first analysis by neighbor net and phi test (Figure 3), the new alignment showed strong evidence for reassortment (*P* < 10^−6^). RDP4 algorithms^28^ confirmed these results, recognizing the correct breakpoint coordinates within 20 nucleotides (Supplemental Document 3). The program results also corroborated the existence of unknown parental strains to the reassorted genomes.

## DISCUSSION

*Keystone orthobunyavirus* is, in many ways, a “forgotten” virus. First identified in 1964,^2^ with a series of subsequent studies documenting its presence at multiple environmental sites in the south and southeastern United States,^6^^–^^12^ there are few data available on the role of KEYV as a human pathogen, due in large part to the lack of a readily available test for clinical infections. Nonetheless, human infections with KEYV would appear to be common in the region, with studies in the 1960s and 1970s reporting that 19% to 21% of serum samples from normal, healthy people in the Tampa Bay region had neutralizing antibodies against the virus.^3^ In keeping with these findings, we recently screened 31 random, deidentified serum samples from the laboratory at UFHealth/Shands Hospital and found that nine (29%) of the 31 samples had neutralizing antibodies to KEYV, with titers as high as 1:40 (personal communication, J. Lednicky). This is comparable to the 28% seroprevalence seen with JCV (currently the third leading cause of arboviral neuroinvasive disease in the United States) in Michigan and the upper Midwest.^34^ Of note, since the implementation of IgM assays for JCV at the CDC in 2013, there has been a striking increase in the number of neuroinvasive JCV cases identified nationally, including first-time reports of cases from eight states,^35^ highlighting the critical importance of having diagnostic tests available to identify infections and recognize associated human illness.

The current study documented the continued circulation of KEYV in regional mosquito populations in Florida, with the virus being isolated from *Ae. atlanticus* mosquito pools. However, further studies are needed to assess the sensitivity of the rRT-PCR assay used in this study, as well as its specificity for other *Orthobunyavirus* species; nonetheless, its incorporation into an easily usable assay kit, as has been done by Aix Marseille Université, is a promising start toward development of human diagnostics. The significance of the positive rRT-PCR assay results obtained in the current study with *Culex* spp. is uncertain. In studies conducted by the CDC, KEYV has been isolated and sequenced from *Culex* species.^13^ Our failure to isolate the virus from *Culex* may reflect issues surrounding viability of the virus in *Culex* and/or viral load. It is also possible that the screening assay was picking up other cross-reacting viruses that were not amenable to our culture techniques.

Our genome sequence data are somewhat difficult to interpret, due in part to the small number of available KEYV sequences. Genetic analysis of the L segment showed evidence of two widely separated clades (86.4% nucleotide identity and 96.97% amino acid identity). The International Committee on Taxonomy of viruses requires less than 96% identity in the complete amino acid sequence of the L segment to define different species within the genus *Orthobunyavirus*.^1^ Although our clades did not quite meet these criteria, the degree of diversity is substantial, and, with additional data, it may be necessary to separate out species or subspecies of KEYV. Further, subsequent analyses suggested the possibility of earlier reassortment events with currently unidentified progenitor strains. Taxonomic classification might need to include whole genome analyses to detect the divergence and appearance of new strains/species after such reassortment events. Clearly, further studies (with sequencing of additional genomes) are needed to sort out underlying evolutionary pathways and possible associations between specific strains and geographic location, host species, and, possibly, clinical outcome.

## Financial Disclosure

Work was supported in part by the Florida Department of Health (contract no. 026365) awarded to J. G. M., by the European Commission (European Virus Archive Global project, grant agreement no. 871029) of the Horizon 2020 Research and Innovation Programme, and the European virus archive—Marseille under the label technological platforms of Aix-Marseille University.

## Supplemental Materials


Supplemental materials

Supplemental materials
