# Supplementary material for: Diversity and Genetic Reassortment of Keystone Virus in Mosquito Populations in Florida
Source: Am J Trop Med Hyg. 2023 May 1;108(6):1256–63. doi: 10.4269/ajtmh.22-0594 (PMC10540117; doi:10.4269/ajtmh.22-0594)
Supplement: Supplementary file 1 [file tpmd220594.SD1.pdf]

MELcxS35-56

```
#KT630290.1_Keystone_virus_strain_KEYV/Ochlerotatus_atlanticus/USA/KEYVLK01/2005 ATGGGTGATT TGGTTTTCTA TGATGTCGCA TCCACAGGTG CAAATGGATT TGATCCTGAT GCAGGGTATG TGGCATTTAT  [ 80]
#KT630293.1_Keystone_virus_strain_KEYV/Ochlerotatus_atlanticus/USA/KEYVLK02/2005 .......... .......... .......... .......... .......... .......... .......... ..........  [ 80]
#MH016786.1_Keystone_virus_strain_KEYV/Homo_sapiens/Gainesville-1/2016_nucleopro .......... .......... .......... .......... .......... .......... .......... ..........  [ 80]
#KX817323.1_Keystone_virus_strain_B64-5587.05_segment_S_complete_sequence        .......... .......... .......... .......... .......... .......... .......... ..........  [ 80]
#MG821231.1_Keystone_virus_isolate_AR14033_segment_S_complete_sequence           .......... .......... .......... .......... .......... .......... .......... ..........  [ 80]
#MG765471.1_Keystone_virus_isolate_AVA1709441_nucleocapsid_and_NSs_genes_complet .......... .......... .......... .......... .......... .......... .......... ..........  [ 80]
#KX817329.1_Melao_virus_strain_TRVL_9375_segment_S_complete_sequence             .....A.... ..A.C..T.. .......... ..A....... .......... ......A... .........C .........C  [ 80]
#KX817335.1_Serra_do_Navio_virus_strain_BeAr_103645_segment_S_complete_sequence  .....A.... .......T.. .......... ..A..C.... .......... ......A... .......T.. ..........  [ 80]
#KX817320.1_Jerry_Slough_virus_strain_BFS_4474_segment_S_complete_sequence       .....A.... .A.....T.. .......... .......... .......... ......A... .......T.. ..........  [ 80]
#KX817338.1_South_River_virus_strain_NJO-94F_segment_S_complete_sequence         .....A.... .......T.. .......... .......... .......... ......A... .......T.. ..........  [ 80]
#GU018050.2_South_River_virus_isolate_SORV-252_nucleoprotein_and_NSs_protein_gen .....A.... ....C..T.. .......... .......... .......... ......A... .......T.. ..........  [ 80]
#EF681804.1_Jamestown_Canyon_virus_isolate_5592-02_segment_S_nucleocapsid_protei .....A...C .......T.. .......... .......... .......... ......A... .......T.. ..........  [ 80]
#EF681805.1_Jamestown_Canyon_virus_isolate_368-99_segment_S_nucleocapsid_protein .....A...C .......T.. .......... .......... .......... ......A... .......T.. ..........  [ 80]
#EF681806.1_Jamestown_Canyon_virus_isolate_6163-03_segment_S_nucleocapsid_protei .....A...C .......T.. .......... .......... .......... ......A... .......T.. ..........  [ 80]
#EF681807.1_Jamestown_Canyon_virus_isolate_468-04_segment_S_nucleocapsid_protein .....A.... .......... .......... .......... .......... ......A... .......T.. ..........  [ 80]
#EF681808.1_Jamestown_Canyon_virus_isolate_2179-00_segment_S_nucleocapsid_protei .....A...C .......T.. .......... .......... .......... ......A... .......T.. ..........  [ 80]
#EF681809.1_Jamestown_Canyon_virus_isolate_779-98_segment_S_nucleocapsid_protein .....A.... .......... .......... .......... .......... ......A... .......T.. ..........  [ 80]
#EF681810.1_Jamestown_Canyon_virus_isolate_810-98_segment_S_nucleocapsid_protein .....A.... .......... .......... .......... .......... ......A... .......T.. ..........  [ 80]
#EF681811.1_Jamestown_Canyon_virus_isolate_811-00_segment_S_nucleocapsid_protein .....A...C .......T.. .......... .......... .......... ......A... .......T.. ..........  [ 80]
#EF681812.1_Jamestown_Canyon_virus_isolate_1697-03_segment_S_nucleocapsid_protei .....A.... .......... .......... .......... .......... ......A... .......T.. ..........  [ 80]
#EF681813.1_Jamestown_Canyon_virus_isolate_1425-02_segment_S_nucleocapsid_protei .....A.... .......... .......... .......... .......... ......A... .......T.. ..........  [ 80]
#EF681814.1_Jamestown_Canyon_virus_isolate_1441-04_segment_S_nucleocapsid_protei .....A.... .......... .......... .......... .......... ......A... .......T.. ..........  [ 80]
#EF681815.1_Jamestown_Canyon_virus_isolate_928-00_segment_S_nucleocapsid_protein .....A.... .......... .......... .......... .......... ......A... .......T.. ..........  [ 80]
#EF681816.1_Jamestown_Canyon_virus_isolate_1064-03_segment_S_nucleocapsid_protei .....A.... .......... .......... .......... .......... ......A... .......T.. ..........  [ 80]
#EF681817.1_Jamestown_Canyon_virus_isolate_1369-02_segment_S_nucleocapsid_protei .....A...C .......T.. .......... .......... .......... ......A... .......T.. ..........  [ 80]
#EF681818.1_Jamestown_Canyon_virus_isolate_1627-04_segment_S_nucleocapsid_protei .....A...C .......T.. .......... .......... .......... ......A... .......T.. ..........  [ 80]
#EF681819.1_Jamestown_Canyon_virus_isolate_1810-02_segment_S_nucleocapsid_protei .....A...C .......T.. .......... .......... .......... ......A... .......T.. ..........  [ 80]
#EF681820.1_Jamestown_Canyon_virus_isolate_2384-98_segment_S_nucleocapsid_protei .....A...C .......T.. .......... .......... .......... ......A... .......T.. ..........  [ 80]
#EF681821.1_Jamestown_Canyon_virus_isolate_2707-01_segment_S_nucleocapsid_protei .....A.... .......... .......... .......... .......... ......A... .......T.. ..........  [ 80]
#EF681822.1_Jamestown_Canyon_virus_isolate_2718-01_segment_S_nucleocapsid_protei .....A...C .......T.. .......... .......... .......... ......A... .......T.. ..........  [ 80]
#EF681823.1_Jamestown_Canyon_virus_isolate_3280-03_segment_S_nucleocapsid_protei .....A.... .......... .......... .......... .......... ......A... .......T.. ..........  [ 80]
#EF681824.1_Jamestown_Canyon_virus_isolate_3324-04_segment_S_nucleocapsid_protei .....A...C .......T.. .......... .......... .......... ......A... .......T.. ..........  [ 80]
#EF681825.1_Jamestown_Canyon_virus_isolate_3573-03_segment_S_nucleocapsid_protei .....A.... .......... .......... .......... .......... ......A... .......T.. ..........  [ 80]
#EF681826.1_Jamestown_Canyon_virus_isolate_3682-00_segment_S_nucleocapsid_protei .....A.... .......... .......... .......... .......... ......A... .......T.. ..........  [ 80]
#EF681827.1_Jamestown_Canyon_virus_isolate_4148-03_segment_S_nucleocapsid_protei .....A.... .......... ......T... .......... .......... ......A... .......T.. ..........  [ 80]
#EF681828.1_Jamestown_Canyon_virus_isolate_4473-00_segment_S_nucleocapsid_protei .....A.... .......... .......... .......... .......... ......A... .......T.. ..........  [ 80]
#EF681829.1_Jamestown_Canyon_virus_isolate_4742-04_segment_S_nucleocapsid_protei .....A...C .......T.. .......... .......... .......... ......A... .......T.. ..........  [ 80]
#EF681830.1_Jamestown_Canyon_virus_isolate_2274-05_segment_S_nucleocapsid_protei .....A...C .......T.. .......... .......... .......... ......A... .......T.. ..........  [ 80]
#EF681831.1_Jamestown_Canyon_virus_isolate_1472-05_segment_S_nucleocapsid_protei .....A...C .......T.. .......... .......... .......... ......A... .......T.. ..........  [ 80]
#EF681832.1_Jamestown_Canyon_virus_isolate_4910-02_segment_S_nucleocapsid_protei .....A.... .......... .......... .......... .......... ......A... .......T.. ..........  [ 80]
#EF681833.1_Jamestown_Canyon_virus_isolate_275-01_segment_S_nucleocapsid_protein .....A...C .......T.. .......... .......... .......... ......A... .......T.. ..........  [ 80]
#EF681834.1_Jamestown_Canyon_virus_isolate_339-05_segment_S_nucleocapsid_protein .....A...C .......T.. .......... .......... .......... ......A... .......T.. ..........  [ 80]
#EF681835.1_Jamestown_Canyon_virus_isolate_3836-05_segment_S_nucleocapsid_protei .....A...C .......T.. .......... .......... .......... ......A... .......T.. ..........  [ 80]
#EF681836.1_Jamestown_Canyon_virus_isolate_2286-00_segment_S_nucleocapsid_protei .....A.... .......... .......... .......... .......... ......A... .......T.. ..........  [ 80]
#EF681837.1_Jamestown_Canyon_virus_isolate_1044-05_segment_S_nucleocapsid_protei .....A...C .......T.. .......... .......... .......... ......A... .......T.. ..........  [ 80]
#EF681838.1_Jamestown_Canyon_virus_isolate_978-99_segment_S_nucleocapsid_protein .....A...C .......T.. .......... .......... .......... ......A... .......T.. ..........  [ 80]
#EF681839.1_Jamestown_Canyon_virus_isolate_4832-01_segment_S_nucleocapsid_protei .....A...C .......T.. .......... .......... .......... ......A... .......T.. ..........  [ 80]
#EF681841.1_Jamestown_Canyon_virus_isolate_7101-03_segment_S_nucleocapsid_protei .....A...C .......T.. .......... .......... .......... ......A... .......T.. ..........  [ 80]
#EF681842.1_Jamestown_Canyon_virus_isolate_Simsbury_segment_S_nucleocapsid_prote .....A...C .......T.. .......... .......... .......... ......A... .......T.. ..........  [ 80]
#EF681843.1_Jamestown_Canyon_virus_isolate_8011-03_segment_S_nucleocapsid_protei .....A...C .......T.. .......... .......... .......... ......A... .......T.. ..........  [ 80]
#EF681844.1_Jamestown_Canyon_virus_isolate_8536-03_segment_S_nucleocapsid_protei .....A.... .......... .......... .......... .......... ......A... .......T.. ..........  [ 80]
#EF681845.1_Jamestown_Canyon_virus_isolate_11497-03_segment_S_nucleocapsid_prote .....A...C .......T.. .......... .......... .......... ......A... .......T.. ..........  [ 80]
#EF681846.1_Jamestown_Canyon_virus_isolate_13995-03_segment_S_nucleocapsid_prote .....A...C .......T.. .......... .......... .......... ......A... .......T.. ..........  [ 80]
#EF681847.1_Jamestown_Canyon_virus_isolate_1768-98_segment_S_nucleocapsid_protei .....A.... .......... .......... .......... .......... ......A... .......T.. ..........  [ 80]
#EF681848.1_Jamestown_Canyon_virus_isolate_1385-06_segment_S_nucleocapsid_protei .....A...C .......T.. .......... .......... .......... ......A... .......T.. ..........  [ 80]
```

```
#EF681849.1_Jamestown_Canyon_virus_isolate_2989-06_segment_S_nucleocapsid_protei .....A...C ..A....T.. .......... .......... .......... ......A... .......T.. ..........  [ 80]
#EF681850.1_Jamestown_Canyon_virus_isolate_3381-06_segment_S_nucleocapsid_protei .....A...C .......T.. .......... .......... .......... ......A... .......T.. ..........  [ 80]
#EF681851.1_Jamestown_Canyon_virus_isolate_4095-06_segment_S_nucleocapsid_protei .....A...C .......T.. .......... .......... .......... ......A... .......T.. ..........  [ 80]
#EF681852.1_Jamestown_Canyon_virus_isolate_4078-06_segment_S_nucleocapsid_protei .....A...C .......T.. .......... .......... .......... ......A... .......T.. ..........  [ 80]
#EF681853.1_Jamestown_Canyon_virus_isolate_11-92_segment_S_nucleocapsid_protein_ .....A.... .......... .......... .......... .......... ......A... .......T.. ..........  [ 80]
#EF681854.1_Jamestown_Canyon_virus_isolate_23-97_segment_S_nucleocapsid_protein_ .....A.... .......... .......... .......... .......... ......A... .......T.. ..........  [ 80]
#EF681855.1_Jamestown_Canyon_virus_isolate_25-97_segment_S_nucleocapsid_protein_ .....A...C .......T.. .......... .......... .......... ......A... .......T.. ..........  [ 80]
#EF681856.1_Jamestown_Canyon_virus_isolate_29-97_segment_S_nucleocapsid_protein_ .....A.... .......... .......... .......... .......... ......A... .......T.. ..........  [ 80]
#EF681857.1_Jamestown_Canyon_virus_isolate_423-99_segment_S_nucleocapsid_protein .....A...C .......T.. .......... .......... .......... ......A... .......T.. ..........  [ 80]
#EF681858.1_Jamestown_Canyon_virus_isolate_1262-98_segment_S_nucleocapsid_protei .....A.... .......... .......... .......... .......... ......A... .......T.. ..........  [ 80]
#EF681859.1_Jamestown_Canyon_virus_isolate_3438-06_segment_S_nucleocapsid_protei .....A...C .......T.. .......... .......... .......... ......A... .......T.. ..........  [ 80]
#HM007350.1_Jamestown_Canyon_virus_strain_61V2235_nucleoprotein_and_NSs_protein_ .....A.... .......... .......... .......... .......... ......A... .......T.. ..........  [ 80]
#HM007353.1_Jamestown_Canyon_virus_strain_3573-03_nucleoprotein_gene_complete_cd .....A.... .......... .......... .......... .......... ......A... .......T.. ..........  [ 80]
#HM007356.1_Jamestown_Canyon_virus_strain_3324-04_nucleoprotein_and_NSs_protein_ .....A...C .......T.. .......... .......... .......... ......A... .......T.. ..........  [ 80]
#KM215518.1_Jamestown_Canyon_virus_isolate_F1819_nucleocapsid_protein_and_nonstr .....A.... .......T.. .......... .......... .......... ......A... .......T.. ..........  [ 80]
#KM215519.1_Jamestown_Canyon_virus_isolate_F6626_nucleocapsid_protein_and_nonstr .....A.... .......T.. .......... .......... .......... ......A... .......T.. ..........  [ 80]
#KM215520.1_Jamestown_Canyon_virus_isolate_ND0283_nucleocapsid_protein_and_nonst .....A.... .......T.. .......... .......... .......... ......A... .......T.. ..........  [ 80]
#KM215521.1_Jamestown_Canyon_virus_isolate_ND6194_nucleocapsid_protein_and_nonst .....A.... .......T.. .......... .......... .......... ......A... .......T.. ..........  [ 80]
#KM215522.1_Jamestown_Canyon_virus_isolate_W6701_nucleocapsid_protein_and_nonstr .....A.... .......T.. .......... .......... .......... ......A... .......T.. ..........  [ 80]
#KM215523.1_Jamestown_Canyon_virus_isolate_W14530_nucleocapsid_protein_and_nonst .....A.... .......T.. .......... .......... .......... ......A... .......T.. ..........  [ 80]
#KM215524.1_Jamestown_Canyon_virus_isolate_W16690_nucleocapsid_protein_and_nonst .....A.... .......T.. .......... .......... .......... ......A... .......T.. ..........  [ 80]
#KM215525.1_Jamestown_Canyon_virus_isolate_W18699_nucleocapsid_protein_and_nonst .....A.... .......... .......... .......... .......... ......A... .......T.. ..........  [ 80]
#KM215526.1_Jamestown_Canyon_virus_isolate_W15316_nucleocapsid_protein_and_nonst .....A.... .......T.. .......... .......... .......... ......A... .......T.. ..........  [ 80]
#KM215527.1_Jamestown_Canyon_virus_isolate_W17680_nucleocapsid_protein_and_nonst .....A.... .......T.. .......... .......... .......... ......A... .......T.. ..........  [ 80]
#KM215528.1_Jamestown_Canyon_virus_isolate_W19543_nucleocapsid_protein_and_nonst .....A.... .......T.. .......... .......... .......... ......A... .......T.. ..........  [ 80]
#KM215529.1_Jamestown_Canyon_virus_isolate_W19925_nucleocapsid_protein_and_nonst .....A.... .......T.. .......... .......... .......... ......A... .......T.. ..........  [ 80]
#KM215530.1_Jamestown_Canyon_virus_isolate_W20764_nucleocapsid_protein_and_nonst .....A.... .......T.. .......... .......... .......... ......A... .......T.. ..........  [ 80]
#KM215531.1_Jamestown_Canyon_virus_isolate_W22352_nucleocapsid_protein_and_nonst .....A...C .......T.. .......... .......... .......... ......A... .......T.. ..........  [ 80]
#KM215532.1_Jamestown_Canyon_virus_isolate_F1829_nucleocapsid_protein_and_nonstr .....A.... .......T.. .......... .......... .......... ......A... .......T.. ..........  [ 80]
#KM215533.1_Jamestown_Canyon_virus_isolate_F6228_nucleocapsid_protein_and_nonstr .....A.... .......T.. .......... .......... .......... ......A... .......T.. ..........  [ 80]
#KM215534.1_Jamestown_Canyon_virus_isolate_F6235_nucleocapsid_protein_and_nonstr .....A.... .......T.. .......... .......... .......... ......A... .......T.. ..........  [ 80]
#KM215535.1_Jamestown_Canyon_virus_isolate_F10095_nucleocapsid_protein_and_nonst .....A.... .......T.. .......... .......... .......... ......A... .......T.. ..........  [ 80]
#KM215536.1_Jamestown_Canyon_virus_isolate_F13418_nucleocapsid_protein_and_nonst .....A.... .......T.. .......... .......... .......... ......A... .......T.. ..........  [ 80]
#KM215537.1_Jamestown_Canyon_virus_isolate_F14162_nucleocapsid_protein_and_nonst .....A.... .......T.. .......... .......... .......... ......A... .......T.. ..........  [ 80]
#KM215538.1_Jamestown_Canyon_virus_isolate_F14183_nucleocapsid_protein_and_nonst .....A.... .......T.. .......... .......... .......... ......A... .......T.. ..........  [ 80]
#KM215539.1_Jamestown_Canyon_virus_isolate_F14278_nucleocapsid_protein_and_nonst .....A.... .......... .......... .......... .......... ......A... .......T.. ..........  [ 80]
#KM215540.1_Jamestown_Canyon_virus_isolate_F16109_nucleocapsid_protein_and_nonst .....A.... .......T.. .......... .......... .......... ......A... .......T.. ..........  [ 80]
#KM215541.1_Jamestown_Canyon_virus_isolate_W8270_nucleocapsid_protein_and_nonstr .....A.... .......T.. .......... .......... .......... ......A... .......T.. ..........  [ 80]
#KM215542.1_Jamestown_Canyon_virus_isolate_W16455_nucleocapsid_protein_and_nonst .....A.... .......T.. .......... .......... .......... ......A... .......T.. ..........  [ 80]
#KM215543.1_Jamestown_Canyon_virus_isolate_W18738_nucleocapsid_protein_and_nonst .....A.... .......T.. .......... .......... .......... ......A... .......T.. ..........  [ 80]
#KM215544.1_Jamestown_Canyon_virus_isolate_F4276_nucleocapsid_protein_and_nonstr .....A.... .......T.. .......... .......... .......... ......A... .......T.. ..........  [ 80]
#KM215545.1_Jamestown_Canyon_virus_isolate_W19962_nucleocapsid_protein_and_nonst .....A.... .......T.. .......... .......... .......... ......A... .......T.. ..........  [ 80]
#KM215546.1_Jamestown_Canyon_virus_isolate_F5609_nucleocapsid_protein_and_nonstr .....A.... .......T.. .......... .......... .......... ......A... .......T.. ..........  [ 80]
#KM215547.1_Jamestown_Canyon_virus_isolate_F7826_nucleocapsid_protein_and_nonstr .....A.... .......T.. .......... .......... .......... ......A... .......T.. ..........  [ 80]
#KM215548.1_Jamestown_Canyon_virus_isolate_F7932_nucleocapsid_protein_and_nonstr .....A.... .......T.. .......... .......... .......... ......A... .......T.. ..........  [ 80]
#KM215549.1_Jamestown_Canyon_virus_isolate_F8532_nucleocapsid_protein_and_nonstr .....A.... .......T.. .......... .......... .......... ......A... .......T.. ..........  [ 80]
#KM215550.1_Jamestown_Canyon_virus_isolate_F10720_nucleocapsid_protein_and_nonst .....A.... .......T.. .......... .......... .......... ......A... .......T.. ..........  [ 80]
#KM215551.1_Jamestown_Canyon_virus_isolate_ND1300_nucleocapsid_protein_and_nonst .....A.... .......T.. .......... .......... .......... ......A... .......T.. ..........  [ 80]
#KM215552.1_Jamestown_Canyon_virus_isolate_W13135_nucleocapsid_protein_and_nonst .....A.... .......T.. .......... .......... .......... ......A... .......T.. ..........  [ 80]
#KM215553.1_Jamestown_Canyon_virus_isolate_W16456_nucleocapsid_protein_and_nonst .....A.... .......T.. .......... .......... .......... ......A... .......T.. ..........  [ 80]
#KM215554.1_Jamestown_Canyon_virus_isolate_W16590_nucleocapsid_protein_and_nonst .....A.... .......T.. .......... .......... .......... ......A... .......T.. ..........  [ 80]
#KM215555.1_Jamestown_Canyon_virus_isolate_W19651_nucleocapsid_protein_and_nonst .....A.... .......T.. .......... .......... .......... ......A... .......T.. ..........  [ 80]
#KM215556.1_Jamestown_Canyon_virus_isolate_F3406_nucleocapsid_protein_and_nonstr .....A.... .......T.. .......... .......... .......... ......A... .......T.. ..........  [ 80]
#KM215557.1_Jamestown_Canyon_virus_isolate_F6577_nucleocapsid_protein_and_nonstr .....A.... .......T.. .......... .......... .......... ......A... .......T.. ..........  [ 80]
#KM215558.1_Jamestown_Canyon_virus_isolate_ND374_nucleocapsid_protein_and_nonstr .....A.... .......T.. .......... .......... .......... ......A... .......T.. ..........  [ 80]
#KM215559.1_Jamestown_Canyon_virus_isolate_W18393_nucleocapsid_protein_and_nonst .....A.... .......... .......... .......... .......... ......A... .......T.. ..........  [ 80]
#KM215560.1_Jamestown_Canyon_virus_isolate_W18901_nucleocapsid_protein_and_nonst .....A.... .......... .......... .......... .......... ......A... .......T.. ..........  [ 80]
```

```
#KX817317.1_Jamestown_Canyon_virus_strain_61V2235_segment_S_complete_sequence    .....A.... .......... .......... .......... .......... ......A... .......T.. ..........  [ 80]
#MH370817.1_Jamestown_Canyon_virus_isolate_L36708_segment_S_complete_sequence    .....A.... .......... .......... .......... .......... ......A... .......T.. ..........  [ 80]
#MH370820.1_Jamestown_Canyon_virus_isolate_MN256-260_segment_S_complete_sequence .....A.... .......T.. .......... .......... .......... ......A... .......T.. ..........  [ 80]
#U12799.1_Jamestown_Canyon_virus_DAV28_S_RNA_segment_N_and_NSs_protein_genes_com .....A.... .......... .......... .......... .......... ......A... .......T.. ..........  [ 80]
#U12796.1_Jamestown_Canyon_virus_61v2235_S_RNA_segment_N_and_NSs_protein_genes_c .....A.... .......... .......... .......... .......... ......A... .......T.. ..........  [ 80]
#KM215561.1_Jamestown_Canyon_virus_isolate_W23697_nucleocapsid_protein_and_nonst .....G.... .......T.. .......... .......... .......... ......A... .......T.. ..........  [ 80]
#KT288271.1_Inkoo_virus_strain_LEIV-15248Iv_segment_S_nucleoprotein_(N)_gene_com .....A.... .......... .......... .......... .......... ......A... .......T.. ..........  [ 80]
#KT288274.1_Inkoo_virus_strain_LEIV-18154Yak_segment_S_nucleoprotein_(N)_gene_co .....A.... .......... .......... .......... .......... ......A... .......T.. ..........  [ 80]
#KT288275.1_Inkoo_virus_strain_LEIV-9874Kar_segment_S_nucleoprotein_(N)_gene_com .....A.... .......... .......... .......... .......... ......A... .......T.. ..........  [ 80]
#KT288277.1_Inkoo_virus_strain_LEIV-18784Yak_segment_S_nucleoprotein_(N)_gene_co .....A.... .......... .......... .......... .......... ......A... .......T.. ..........  [ 80]
#KT288280.1_Inkoo_virus_strain_LEIV-22780Tyum_segment_S_nucleoprotein_(N)_gene_c .....A.... .......... .......... .......... .......... ......A... .......T.. ..........  [ 80]
#KT288283.1_Inkoo_virus_strain_LEIV-18152Yak_segment_S_nucleoprotein_(N)_gene_co .....A.... .......... .......... .......... .......... ......A... .......T.. ..........  [ 80]
#KT288286.1_Inkoo_virus_strain_LEIV-21643Kra_segment_S_nucleoprotein_(N)_gene_co .....A.... .......... .......... .......... .......... ......A... .......T.. ..........  [ 80]
#KX554935.1_Inkoo_virus_strain_Lovanger_nucleocapsid_protein_and_nonstructural_p .....A.... .......... .......... .......... .......... ......A... .......T.. ..........  [ 80]
#U47137.1_Inkoo_virus_Prototype_KN3641_nucleocapsid_protein_and_non-structural_p .....A.... .......... ......T... .......... .......... ......A... .......T.. ..........  [ 80]
#U47138.1_Inkoo_virus_SW_AR_83-161_nucleocapsid_protein_and_non-structural_prote .....A.... .......... .......... .......... .......... ......A... .......T.. ..........  [ 80]
#Z68496.1_Inkoo_virus_RNA_for_N_protein_and_RNA_for_NS_protein_strain_KN_3641    .....A.... .......... .....AA... .......... .......... ......A... .......T.. ..........  [ 80]
```

MELcxP113-137 MELcxR164-145

```
#KT630290.1_Keystone_virus_strain_KEYV/Ochlerotatus_atlanticus/USA/KEYVLK01/2005 GGCTAACCAT GGGGAGTCGA TCAGTCTGTC AACCGTTAGG ATCTTCTTCC TTAATGCCGC AAAGGCCAAA GCTGCTCTCA  [160]
#KT630293.1_Keystone_virus_strain_KEYV/Ochlerotatus_atlanticus/USA/KEYVLK02/2005 .......... .......... .......... .......... .......... .......... ...A...... ..........  [160]
#MH016786.1_Keystone_virus_strain_KEYV/Homo_sapiens/Gainesville-1/2016_nucleopro .......... .......... .......... .......... .......... .......... .......... ..........  [160]
#KX817323.1_Keystone_virus_strain_B64-5587.05_segment_S_complete_sequence        .......... .......... .......... .......... .......... .......... .......... ..........  [160]
#MG821231.1_Keystone_virus_isolate_AR14033_segment_S_complete_sequence           .........C ....A..A.. .......... .......... .....T.... .......... ...A...... A........G  [160]
#MG765471.1_Keystone_virus_isolate_AVA1709441_nucleocapsid_and_NSs_genes_complet .........C ....A..... .......... .......... .....T.... .......... ...A...... A........G  [160]
#KX817329.1_Melao_virus_strain_TRVL_9375_segment_S_complete_sequence             AAT.GCG... .....AG.T. ...A.T.... .G........ .......... .......... ...A...... .........T  [160]
#KX817335.1_Serra_do_Navio_virus_strain_BeAr_103645_segment_S_complete_sequence  ....GGT... .......... ...A...TA. .G........ .......... .......... ...A...... .........T  [160]
#KX817320.1_Jerry_Slough_virus_strain_BFS_4474_segment_S_complete_sequence       ....G....C ..A.....T. ...A...... .G........ .......... .......A.. ...A...... .........G  [160]
#KX817338.1_South_River_virus_strain_NJO-94F_segment_S_complete_sequence         ....G..... ..A..A..T. ...A...... .G........ .......... .......A.. ...A...... .........G  [160]
#GU018050.2_South_River_virus_isolate_SORV-252_nucleoprotein_and_NSs_protein_gen ....G..... ..A..A..T. .......... .G........ .......... .......A.. .......... .........G  [160]
#EF681804.1_Jamestown_Canyon_virus_isolate_5592-02_segment_S_nucleocapsid_protei ....G..... .....A..TG .......... .G........ .......... .......A.. ...A...... .........G  [160]
#EF681805.1_Jamestown_Canyon_virus_isolate_368-99_segment_S_nucleocapsid_protein ....G..... .....A..TG .......... .G........ .......... .......A.. ...A...... .........G  [160]
#EF681806.1_Jamestown_Canyon_virus_isolate_6163-03_segment_S_nucleocapsid_protei ....G..... .....A..TG .......... .G........ .......... .......A.. ...A...... .........G  [160]
#EF681807.1_Jamestown_Canyon_virus_isolate_468-04_segment_S_nucleocapsid_protein ....G....C .....AGTT. ...A....G. .G........ .......... .......A.. ...A...... .........G  [160]
#EF681808.1_Jamestown_Canyon_virus_isolate_2179-00_segment_S_nucleocapsid_protei ....G..... .....A..TG .......... .G........ .......... .......A.. ...A...... .........G  [160]
#EF681809.1_Jamestown_Canyon_virus_isolate_779-98_segment_S_nucleocapsid_protein ....G....C .....AGTT. ...A....G. .G........ .......... .......A.. ...A...... .........G  [160]
#EF681810.1_Jamestown_Canyon_virus_isolate_810-98_segment_S_nucleocapsid_protein ....G....C .....AGTT. ...A....G. .G........ .......... .......A.. ...A...... .........G  [160]
#EF681811.1_Jamestown_Canyon_virus_isolate_811-00_segment_S_nucleocapsid_protein ....G..... .....A..TG .......... .G........ .......... .......A.. ...A...... .........G  [160]
#EF681812.1_Jamestown_Canyon_virus_isolate_1697-03_segment_S_nucleocapsid_protei ....G....C .....AGTT. ...A....G. .G........ .......... .......A.. ...A...... .........G  [160]
#EF681813.1_Jamestown_Canyon_virus_isolate_1425-02_segment_S_nucleocapsid_protei ....G....C .....AGTT. ...A....G. .G........ .......... .......A.. ...A...... .........G  [160]
#EF681814.1_Jamestown_Canyon_virus_isolate_1441-04_segment_S_nucleocapsid_protei ....G....C .....AGTT. ...A....G. .G........ .......... .......A.. ...A...... .........G  [160]
#EF681815.1_Jamestown_Canyon_virus_isolate_928-00_segment_S_nucleocapsid_protein ....G....C .....AGTT. ...A....G. .G........ .......... .......A.. ...A...... .........G  [160]
#EF681816.1_Jamestown_Canyon_virus_isolate_1064-03_segment_S_nucleocapsid_protei ....G..... .....AG.T. ...A.T..G. .G........ .......... .......A.. ...A...... .........G  [160]
#EF681817.1_Jamestown_Canyon_virus_isolate_1369-02_segment_S_nucleocapsid_protei ....G..... .....A..TG .......... .G........ .......... .......A.. ...A...... .........G  [160]
#EF681818.1_Jamestown_Canyon_virus_isolate_1627-04_segment_S_nucleocapsid_protei ....G..... .....A..TG .......... .G........ .......... .......A.. ...A...... .........G  [160]
#EF681819.1_Jamestown_Canyon_virus_isolate_1810-02_segment_S_nucleocapsid_protei ....G..... .....A..TG .......... .G........ .......... .......A.. ...A...... .........G  [160]
#EF681820.1_Jamestown_Canyon_virus_isolate_2384-98_segment_S_nucleocapsid_protei ....G..... .....A..TG .......... .G........ .......... .......G.. ...A...... .........G  [160]
#EF681821.1_Jamestown_Canyon_virus_isolate_2707-01_segment_S_nucleocapsid_protei ....G....C .....AGTT. ...A....G. .G........ .......... .......A.. ...A...... .........G  [160]
#EF681822.1_Jamestown_Canyon_virus_isolate_2718-01_segment_S_nucleocapsid_protei ....G..... .....A..TG .......... .G........ .......... .......A.. ...A...... .........G  [160]
#EF681823.1_Jamestown_Canyon_virus_isolate_3280-03_segment_S_nucleocapsid_protei ....G....C .....AGTT. ...A....G. .G........ .......... .......A.. ...A...... .........G  [160]
#EF681824.1_Jamestown_Canyon_virus_isolate_3324-04_segment_S_nucleocapsid_protei ....G..... .....A..TG .......... .G........ .......... .......A.. ...A...... .........G  [160]
#EF681825.1_Jamestown_Canyon_virus_isolate_3573-03_segment_S_nucleocapsid_protei ....G....C .....AGTT. ...A....G. .G........ .......... .......A.. ...A...... .........G  [160]
#EF681826.1_Jamestown_Canyon_virus_isolate_3682-00_segment_S_nucleocapsid_protei ....G....C .....AGTT. ...A....G. .G........ .......... .......A.. ...A...... .........G  [160]
#EF681827.1_Jamestown_Canyon_virus_isolate_4148-03_segment_S_nucleocapsid_protei ....G..... .....AG.T. ...A.T..G. .G........ .......... .......A.. ...A...... .........G  [160]
#EF681828.1_Jamestown_Canyon_virus_isolate_4473-00_segment_S_nucleocapsid_protei ....G..... .....AG.T. ...A.T..G. .G........ .......... .......A.. ...A...... .........G  [160]
#EF681829.1_Jamestown_Canyon_virus_isolate_4742-04_segment_S_nucleocapsid_protei ....G..... .....A..TG .......... .G........ .......... .......A.. ...A...... .........G  [160]
#EF681830.1_Jamestown_Canyon_virus_isolate_2274-05_segment_S_nucleocapsid_protei ....G..... .....A..TG .......... .G........ .......... .......A.. ...A...... .........G  [160]
#EF681831.1_Jamestown_Canyon_virus_isolate_1472-05_segment_S_nucleocapsid_protei ....G..... .....A..TG .......... .G........ .......... .......A.. ...A...... .........G  [160]
#EF681832.1_Jamestown_Canyon_virus_isolate_4910-02_segment_S_nucleocapsid_protei ....G....C .....AGTT. ...A....G. .G........ .......... .......A.. ...A...... .........G  [160]
#EF681833.1_Jamestown_Canyon_virus_isolate_275-01_segment_S_nucleocapsid_protein ....G..... .....A..TG .......... .G........ .......... .......A.. ...A...... .........G  [160]
#EF681834.1_Jamestown_Canyon_virus_isolate_339-05_segment_S_nucleocapsid_protein ....G..... .....A..TG .......... .G........ .......... .......A.. ...A...... .........G  [160]
#EF681835.1_Jamestown_Canyon_virus_isolate_3836-05_segment_S_nucleocapsid_protei ....G..... .....A..TG .......... .G........ .......... .......A.. ...A...... .........G  [160]
#EF681836.1_Jamestown_Canyon_virus_isolate_2286-00_segment_S_nucleocapsid_protei ....G....C .....AGTT. ...A....G. .G........ .......... .......A.. ...A...... .........G  [160]
#EF681837.1_Jamestown_Canyon_virus_isolate_1044-05_segment_S_nucleocapsid_protei ....G..... .....A..TG .......... .G........ .......... .......A.. ...A...... .........G  [160]
#EF681838.1_Jamestown_Canyon_virus_isolate_978-99_segment_S_nucleocapsid_protein ....G..... .....A..TG .......... .G........ .......... .......A.. ...A...... .........G  [160]
#EF681839.1_Jamestown_Canyon_virus_isolate_4832-01_segment_S_nucleocapsid_protei ....G..... .....A..TG .......... .G........ .......... .......A.. ...A...... .........G  [160]
#EF681841.1_Jamestown_Canyon_virus_isolate_7101-03_segment_S_nucleocapsid_protei ....G..... .....A..TG .......... .G........ .......... .......A.. ...A...... .........G  [160]
#EF681842.1_Jamestown_Canyon_virus_isolate_Simsbury_segment_S_nucleocapsid_prote ....G..... .....A..TG .......... .G........ .......... .......A.. ...A...... .........G  [160]
#EF681843.1_Jamestown_Canyon_virus_isolate_8011-03_segment_S_nucleocapsid_protei ....G..... .....A..TG .......... .G........ .......... .......G.. ...A...... .........G  [160]
#EF681844.1_Jamestown_Canyon_virus_isolate_8536-03_segment_S_nucleocapsid_protei ....G....C .....AGTT. ...A....G. .G........ .......... .......A.. ...A...... .........G  [160]
#EF681845.1_Jamestown_Canyon_virus_isolate_11497-03_segment_S_nucleocapsid_prote ....G..... .....A..TG .......... .G........ .......... .......A.. ...A...... .........G  [160]
#EF681846.1_Jamestown_Canyon_virus_isolate_13995-03_segment_S_nucleocapsid_prote ....G..... .....A..TG .......... .G........ .......... .......A.. ...A...... .........G  [160]
#EF681847.1_Jamestown_Canyon_virus_isolate_1768-98_segment_S_nucleocapsid_protei ....G....C .....AGTT. ...A....G. .G........ .......... .......A.. ...A...... .........G  [160]
#EF681848.1_Jamestown_Canyon_virus_isolate_1385-06_segment_S_nucleocapsid_protei ....G..... .....A..TG .......... .G........ .......... .......A.. ...A...... .........G  [160]
```

#EF681849.1_Jamestown_Canyon_virus_isolate_2989-06_segment_S_nucleocapsid_protei ....G..... .....A..TG .... .G........ ....A.. ...A...... ....G [160]
#EF681850.1_Jamestown_Canyon_virus_isolate_3381-06_segment_S_nucleocapsid_protei ....G..... .....A..TG .... .G........ ....A.. ...A...... ....G [160]
#EF681851.1_Jamestown_Canyon_virus_isolate_4095-06_segment_S_nucleocapsid_protei ....G..... .....A..TG .... .G........ ....A.. ...A...... ....G [160]
#EF681852.1_Jamestown_Canyon_virus_isolate_4078-06_segment_S_nucleocapsid_protei ....G..... .....A..TG .... .G........ ....A.. ...A...... ....G [160]
#EF681853.1_Jamestown_Canyon_virus_isolate_11-92_segment_S_nucleocapsid_protein_ ....G....C .....AGTT. ...A....G. .G........ ....A.. ...A...... ....G [160]
#EF681854.1_Jamestown_Canyon_virus_isolate_23-97_segment_S_nucleocapsid_protein_ ....G....C .....AGTT. ...A....G. .G........ ....A.. ...A...... ....G [160]
#EF681855.1_Jamestown_Canyon_virus_isolate_25-97_segment_S_nucleocapsid_protein_ ....G..... .....A..TG .... .G........ ....A.. ...A...... ....G [160]
#EF681856.1_Jamestown_Canyon_virus_isolate_29-97_segment_S_nucleocapsid_protein_ ....G....C .....AGTT. ...A....G. .G........ ....A.. ...A...... ....G [160]
#EF681857.1_Jamestown_Canyon_virus_isolate_423-99_segment_S_nucleocapsid_protein ....G..... .....A..TG .... .G........ ....A.. ...A...... ....G [160]
#EF681858.1_Jamestown_Canyon_virus_isolate_1262-98_segment_S_nucleocapsid_protei ....G....C .....AGTT. ...A....G. .G........ ....A.. ...A...... ....G [160]
#EF681859.1_Jamestown_Canyon_virus_isolate_3438-06_segment_S_nucleocapsid_protei ....G..... .....A..TG .... .G........ ....A.. ...A...... ....G [160]
#HM007350.1_Jamestown_Canyon_virus_strain_61V2235_nucleoprotein_and_NSs_protein_ ....G....C ..A.....T. ...A...... .G........ ....A.. ...A...... ....G [160]
#HM007353.1_Jamestown_Canyon_virus_strain_3573-03_nucleoprotein_gene_complete_cd ....G....C .....AGTT. ...A....G. .G........ ....A.. ...A...... ....G [160]
#HM007356.1_Jamestown_Canyon_virus_strain_3324-04_nucleoprotein_and_NSs_protein_ ....G..... .....A..TG .... .G........ ....A.. ...A...... ....G [160]
#KM215518.1_Jamestown_Canyon_virus_isolate_F1819_nucleocapsid_protein_and_nonstr ....G..... .....A..TG .... .G........ ....A.. ...A...... ....G [160]
#KM215519.1_Jamestown_Canyon_virus_isolate_F6626_nucleocapsid_protein_and_nonstr ....G..... .....A..TG .... .G........ ....A.. ...A...... ....G [160]
#KM215520.1_Jamestown_Canyon_virus_isolate_ND0283_nucleocapsid_protein_and_nonst ....G..... .....A..TG .... .G........ ....A.. ...A...... ....G [160]
#KM215521.1_Jamestown_Canyon_virus_isolate_ND6194_nucleocapsid_protein_and_nonst ....G..... .....A..TG .... .G........ ....A.. ...A...... ....G [160]
#KM215522.1_Jamestown_Canyon_virus_isolate_W6701_nucleocapsid_protein_and_nonstr ....G..... .....A..TG .... .G........ ....A.. ...A...... ....G [160]
#KM215523.1_Jamestown_Canyon_virus_isolate_W14530_nucleocapsid_protein_and_nonst ....G..... .....A..TG .... .G........ ....A.. ...A...... ....G [160]
#KM215524.1_Jamestown_Canyon_virus_isolate_W16690_nucleocapsid_protein_and_nonst ....G..... .....A..TG .... .G........ ....A.. ...A...... ....G [160]
#KM215525.1_Jamestown_Canyon_virus_isolate_W18699_nucleocapsid_protein_and_nonst ....G....C ..A.....T. ...A...... .G........ ....A.. ...A...... ....G [160]
#KM215526.1_Jamestown_Canyon_virus_isolate_W15316_nucleocapsid_protein_and_nonst ....G..... .....A..TG .....T.... .G........ ....A.. ...A...... ....G [160]
#KM215527.1_Jamestown_Canyon_virus_isolate_W17680_nucleocapsid_protein_and_nonst ....G..... .....A..TG .... .G........ ....A.. ...A...... ....G [160]
#KM215528.1_Jamestown_Canyon_virus_isolate_W19543_nucleocapsid_protein_and_nonst ....G..... .....A..TG .... .G........ ....A.. ...A...... ....G [160]
#KM215529.1_Jamestown_Canyon_virus_isolate_W19925_nucleocapsid_protein_and_nonst ....G..... .....A..TG .... .G........ ....A.. ...A...... ....G [160]
#KM215530.1_Jamestown_Canyon_virus_isolate_W20764_nucleocapsid_protein_and_nonst ....G..... .....A..TG .... .G........ ....A.. ...A...... ....G [160]
#KM215531.1_Jamestown_Canyon_virus_isolate_W22352_nucleocapsid_protein_and_nonst ....G..... .....A..TG .... .G........ ....A.. ...A...... ....G [160]
#KM215532.1_Jamestown_Canyon_virus_isolate_F1829_nucleocapsid_protein_and_nonstr ....G..... .....A..TG .... .G........ ....A.. ...A...... ....G [160]
#KM215533.1_Jamestown_Canyon_virus_isolate_F6228_nucleocapsid_protein_and_nonstr ....G..... .....A..TG .... .G........ ....A.. ...A...... ....G [160]
#KM215534.1_Jamestown_Canyon_virus_isolate_F6235_nucleocapsid_protein_and_nonstr ....G..... .....A..TG .... .G........ ....A.. ...A...... ....G [160]
#KM215535.1_Jamestown_Canyon_virus_isolate_F10095_nucleocapsid_protein_and_nonst ....G..... .....A..TG .... .G........ ....A.. ...A...... ....G [160]
#KM215536.1_Jamestown_Canyon_virus_isolate_F13418_nucleocapsid_protein_and_nonst ....G..... .....A..TG .... .G........ ....A.. ...A...... ....G [160]
#KM215537.1_Jamestown_Canyon_virus_isolate_F14162_nucleocapsid_protein_and_nonst ....G..... .....A..TG .... .G........ ....A.. ...A...... ....G [160]
#KM215538.1_Jamestown_Canyon_virus_isolate_F14183_nucleocapsid_protein_and_nonst ....G..... .....A..TG .... .G........ ....A.. ...A...... ....G [160]
#KM215539.1_Jamestown_Canyon_virus_isolate_F14278_nucleocapsid_protein_and_nonst ....G..... .....A..TG .... .G........ ....A.. ...A...... ....G [160]
#KM215540.1_Jamestown_Canyon_virus_isolate_F16109_nucleocapsid_protein_and_nonst ....G..... .....A..TG .... .G........ ....A.. ...A...... ....G [160]
#KM215541.1_Jamestown_Canyon_virus_isolate_W8270_nucleocapsid_protein_and_nonstr ....G..... .....A..TG .... .G........ ....A.. ...A...... ....G [160]
#KM215542.1_Jamestown_Canyon_virus_isolate_W16455_nucleocapsid_protein_and_nonst ....G..... .....A..TG .... .G........ ....A.. ...A...... ....G [160]
#KM215543.1_Jamestown_Canyon_virus_isolate_W18738_nucleocapsid_protein_and_nonst ....G..... .....A..TG .... .G........ ....A.. ...A...... ....G [160]
#KM215544.1_Jamestown_Canyon_virus_isolate_F4276_nucleocapsid_protein_and_nonstr ....G..... .....A..TG .... .G........ ....A.. ...A...... ....G [160]
#KM215545.1_Jamestown_Canyon_virus_isolate_W19962_nucleocapsid_protein_and_nonst ....G..... .....A..TG .... .G........ ....A.. ...A...... ....G [160]
#KM215546.1_Jamestown_Canyon_virus_isolate_F5609_nucleocapsid_protein_and_nonstr ....G..... .....A..TG .... .G........ ....A.. ...A...... ....G [160]
#KM215547.1_Jamestown_Canyon_virus_isolate_F7826_nucleocapsid_protein_and_nonstr ....G..... .....A..TG .... .G........ ....A.. ...A...... ....G [160]
#KM215548.1_Jamestown_Canyon_virus_isolate_F7932_nucleocapsid_protein_and_nonstr ....G..... .....A..TG .... .G........ ....A.. ...A...... ....G [160]
#KM215549.1_Jamestown_Canyon_virus_isolate_F8532_nucleocapsid_protein_and_nonstr ....G..... .....A..TG .... .G........ ....A.. ...A...... ....G [160]
#KM215550.1_Jamestown_Canyon_virus_isolate_F10720_nucleocapsid_protein_and_nonst ....G..... .....A..TG .... .G........ ....A.. ...A...... ....G [160]
#KM215551.1_Jamestown_Canyon_virus_isolate_ND1300_nucleocapsid_protein_and_nonst ....G..... .....A..TG .... .G........ .....T.... ....A.. ...A...... ....G [160]
#KM215552.1_Jamestown_Canyon_virus_isolate_W13135_nucleocapsid_protein_and_nonst ....G..... .....A..TG ...A...... .G........ ....A.. ...A...... ....G [160]
#KM215553.1_Jamestown_Canyon_virus_isolate_W16456_nucleocapsid_protein_and_nonst ....G..... .....A..TG .... .G........ ....A.. ...A...... ....G [160]
#KM215554.1_Jamestown_Canyon_virus_isolate_W16590_nucleocapsid_protein_and_nonst ....G..... .....A..TG ...A...... .G........ ....A.. ...A...... ....G [160]
#KM215555.1_Jamestown_Canyon_virus_isolate_W19651_nucleocapsid_protein_and_nonst ....G..... .....A..TG .... .G........ ....A.. ...A...... ....G [160]
#KM215556.1_Jamestown_Canyon_virus_isolate_F3406_nucleocapsid_protein_and_nonstr ....G..... .....A..TG .... .G........ ....A.. ...A...... ....G [160]
#KM215557.1_Jamestown_Canyon_virus_isolate_F6577_nucleocapsid_protein_and_nonstr ....G..... .....A..TG .... .G........ ....A.. ...A...... ....T [160]
#KM215558.1_Jamestown_Canyon_virus_isolate_ND374_nucleocapsid_protein_and_nonstr ....G..... .....A..TG .... .G........ ....A.. ...A...... ....G [160]
#KM215559.1_Jamestown_Canyon_virus_isolate_W18393_nucleocapsid_protein_and_nonst ....G....C ..A.....T. ...A...... .G........ ....A.. ...A...... ....G [160]
#KM215560.1_Jamestown_Canyon_virus_isolate_W18901_nucleocapsid_protein_and_nonst ....G....C ..A.....T. ...A...... .G........ ....A.. ...A...... ....G [160]

```
#KX817317.1_Jamestown_Canyon_virus_strain_61V2235_segment_S_complete_sequence    ....G....C ..A.....T. ...A...... .G........ .......... ......A... ...A...... .........G  [160]
#MH370817.1_Jamestown_Canyon_virus_isolate_L36708_segment_S_complete_sequence    ....G....C .....AGTT. ...A....G. .G........ .......... ......A... ...A...... .........G  [160]
#MH370820.1_Jamestown_Canyon_virus_isolate_MN256-260_segment_S_complete_sequence ....G..... .....A..TG .......... .G........ .......... ......A... ...A...... .........G  [160]
#U12799.1_Jamestown_Canyon_virus_DAV28_S_RNA_segment_N_and_NSs_protein_genes_com ....G....C ..A.....T. ...A...... TG........ .......... ......A... ...A...... .........G  [160]
#U12796.1_Jamestown_Canyon_virus_61v2235_S_RNA_segment_N_and_NSs_protein_genes_c ....G....C ..A.....T. ...A...... .G........ .......... ......A... ...A...... .........G  [160]
#KM215561.1_Jamestown_Canyon_virus_isolate_W23697_nucleocapsid_protein_and_nonst ....G..... .....A..TG .......... .G........ .......... ......A... ...A...... .........G  [160]
#KT288271.1_Inkoo_virus_strain_LEIV-15248Iv_segment_S_nucleoprotein_(N)_gene_com ....G..... ..A..A..T. ...A.T..G. .G........ .......... ......A... ...A...... .........G  [160]
#KT288274.1_Inkoo_virus_strain_LEIV-18154Yak_segment_S_nucleoprotein_(N)_gene_co ....G..... ..A..A..T. ...A.T..G. .G........ .......... ......A... ...A...... .........G  [160]
#KT288275.1_Inkoo_virus_strain_LEIV-9874Kar_segment_S_nucleoprotein_(N)_gene_com ....G..... ..A..A..T. ...A.T..G. .G........ .......... ......A... ...A...... .........G  [160]
#KT288277.1_Inkoo_virus_strain_LEIV-18784Yak_segment_S_nucleoprotein_(N)_gene_co ....G..... ..A..A..T. ...A.T..G. .G........ .......... ......A... ...A...... .........G  [160]
#KT288280.1_Inkoo_virus_strain_LEIV-22780Tyum_segment_S_nucleoprotein_(N)_gene_c ....G..... ..A..A..T. ...A.T..G. .G........ .......... ......A... ...A...... .........G  [160]
#KT288283.1_Inkoo_virus_strain_LEIV-18152Yak_segment_S_nucleoprotein_(N)_gene_co ....G..... ..A..A..T. ...A.T..G. .G........ .......... ......A... ...A...... .........G  [160]
#KT288286.1_Inkoo_virus_strain_LEIV-21643Kra_segment_S_nucleoprotein_(N)_gene_co ....G..... ..A..A..T. ...A.T..G. .G........ .......... ......A... .......... .........G  [160]
#KX554935.1_Inkoo_virus_strain_Lovanger_nucleocapsid_protein_and_nonstructural_p ....G..... ..A..A..T. ...A.T..G. .G........ .......... ......A... ...A...... .........G  [160]
#U47137.1_Inkoo_virus_Prototype_KN3641_nucleocapsid_protein_and_non-structural_p ....G..... ..A..A..T. ...A.T..G. .G........ .......... ......A... ...A...... .........G  [160]
#U47138.1_Inkoo_virus_SW_AR_83-161_nucleocapsid_protein_and_non-structural_prote ....G..... ..A..A..T. .T.A.T..G. .G........ .......... ......A... ...A...... .........G  [160]
#Z68496.1_Inkoo_virus_RNA_for_N_protein_and_RNA_for_NS_protein_strain_KN_3641    ....G..... ..A..A..T. ...A.T..G. .G........ .......... ......A... ...A...... .........G  [160]
```

```
                                                                                                                     MELcxS193-212                   MELcxP218-244
#KT630290.1_Keystone_virus_strain_KEYV/Ochlerotatus_atlanticus/USA/KEYVLK01/2005 CTCGTAAGCC GGAGCGGAAG GCTACACCTA AGTTTGGAGA GTGGCAGGTG GAGATCGTCA ATAATCATTT TCCTGGAAAC  [240]
#KT630293.1_Keystone_virus_strain_KEYV/Ochlerotatus_atlanticus/USA/KEYVLK02/2005 ........T. .......... .......... .......... .......... .......... .......... ..........  [240]
#MH016786.1_Keystone_virus_strain_KEYV/Homo_sapiens/Gainesville-1/2016_nucleopro .......... .......... .......... .......... .......... .......... .......... ..........  [240]
#KX817323.1_Keystone_virus_strain_B64-5587.05_segment_S_complete_sequence        .......... .......... .......... .......... .......... .......... .......... ..........  [240]
#MG821231.1_Keystone_virus_isolate_AR14033_segment_S_complete_sequence           .......... .......... .......... .......... .......... .......... .......... ..........  [240]
#MG765471.1_Keystone_virus_isolate_AVA1709441_nucleocapsid_and_NSs_genes_complet .......... .......... .......... .......... .......... .......... .......... ..........  [240]
#KX817329.1_Melao_virus_strain_TRVL_9375_segment_S_complete_sequence             .......A.. ....A....A .....T.... .A........ C......... ..A..T.... .C........ ..........  [240]
#KX817335.1_Serra_do_Navio_virus_strain_BeAr_103645_segment_S_complete_sequence  ....C..... ....A....A .....T.... .......... C......... .....T.... .......... ..........  [240]
#KX817320.1_Jerry_Slough_virus_strain_BFS_4474_segment_S_complete_sequence       .......A.. .........A .....T.... .......... .......... ......A... .......... ..........  [240]
#KX817338.1_South_River_virus_strain_NJO-94F_segment_S_complete_sequence         .......A.. .......... ....AT.... .A........ .......... .....T.... .......... ..........  [240]
#GU018050.2_South_River_virus_isolate_SORV-252_nucleoprotein_and_NSs_protein_gen .......A.. .......... ....AT.... .A........ .......... .....T.... .......... ..........  [240]
#EF681804.1_Jamestown_Canyon_virus_isolate_5592-02_segment_S_nucleocapsid_protei .......A.. .........A .....T.... .A........ .......... ..A..T.... .......... ..........  [240]
#EF681805.1_Jamestown_Canyon_virus_isolate_368-99_segment_S_nucleocapsid_protein .......A.. .........A .....T.... .A........ .......... ..A..T.... .......... ..........  [240]
#EF681806.1_Jamestown_Canyon_virus_isolate_6163-03_segment_S_nucleocapsid_protei .......A.. .........A .....T.... .A........ .......... ..A..T.... .......... ..........  [240]
#EF681807.1_Jamestown_Canyon_virus_isolate_468-04_segment_S_nucleocapsid_protein .......A.. .........A .....T.... .......... .......... .....T.... .......... ......G...  [240]
#EF681808.1_Jamestown_Canyon_virus_isolate_2179-00_segment_S_nucleocapsid_protei .......A.. .........A .....T.... .A........ .......... ..A..T.... .......... ..........  [240]
#EF681809.1_Jamestown_Canyon_virus_isolate_779-98_segment_S_nucleocapsid_protein .......A.. .........A .....T.... .......... .......... .....T.... .......... ......G...  [240]
#EF681810.1_Jamestown_Canyon_virus_isolate_810-98_segment_S_nucleocapsid_protein .......A.. .........A .....T.... .......... .......... .....T.... .......... ......G...  [240]
#EF681811.1_Jamestown_Canyon_virus_isolate_811-00_segment_S_nucleocapsid_protein .......A.. .........A .....T.... .A........ .......... ..A..T.... .......... ..........  [240]
#EF681812.1_Jamestown_Canyon_virus_isolate_1697-03_segment_S_nucleocapsid_protei .......A.. .........A .....T.... .......... .......... .....T.... .......... ......G...  [240]
#EF681813.1_Jamestown_Canyon_virus_isolate_1425-02_segment_S_nucleocapsid_protei .......A.. .........A .....T.... .......... .......... .....T.... .......... ......G...  [240]
#EF681814.1_Jamestown_Canyon_virus_isolate_1441-04_segment_S_nucleocapsid_protei .......A.. .........A .....T.... .......... .......... .....T.... .......... ......G...  [240]
#EF681815.1_Jamestown_Canyon_virus_isolate_928-00_segment_S_nucleocapsid_protein .......A.. .........A .....T.... .......... .......... .....T.... .......... ......G...  [240]
#EF681816.1_Jamestown_Canyon_virus_isolate_1064-03_segment_S_nucleocapsid_protei .......A.. .........A .....T.... .......... .......... .....T.... .......... ..........  [240]
#EF681817.1_Jamestown_Canyon_virus_isolate_1369-02_segment_S_nucleocapsid_protei .......A.. .........A .....T.... .A........ .......... ..A..T.... .......... ..........  [240]
#EF681818.1_Jamestown_Canyon_virus_isolate_1627-04_segment_S_nucleocapsid_protei .......A.. .........A .....T.... .A........ .......... ..A..T.... .......... ..........  [240]
#EF681819.1_Jamestown_Canyon_virus_isolate_1810-02_segment_S_nucleocapsid_protei .......A.. .........A .....T.... .A........ .......... ..A..T.... .......... ..........  [240]
#EF681820.1_Jamestown_Canyon_virus_isolate_2384-98_segment_S_nucleocapsid_protei .......A.. .........A .....T.... .A........ .......... ..A..T.... .......... ..........  [240]
#EF681821.1_Jamestown_Canyon_virus_isolate_2707-01_segment_S_nucleocapsid_protei .......A.. .........A .....T.... .......... .......... .....T.... .......... ......G...  [240]
#EF681822.1_Jamestown_Canyon_virus_isolate_2718-01_segment_S_nucleocapsid_protei .......A.. .........A .....T.... .A........ .......... ..A..T.... .......... ..........  [240]
#EF681823.1_Jamestown_Canyon_virus_isolate_3280-03_segment_S_nucleocapsid_protei .......A.. .........A .....T.... .......... .......... .....T.... .......... ......G...  [240]
#EF681824.1_Jamestown_Canyon_virus_isolate_3324-04_segment_S_nucleocapsid_protei .......A.. .........A .....T.... .A........ .......... ..A..T.... .......... ..........  [240]
#EF681825.1_Jamestown_Canyon_virus_isolate_3573-03_segment_S_nucleocapsid_protei .......A.. .........A .....T.... .......... .......... .....T.... .......... ......G...  [240]
#EF681826.1_Jamestown_Canyon_virus_isolate_3682-00_segment_S_nucleocapsid_protei .......A.. .........A .....T.... .......... .......... .....T.... .......... ......G...  [240]
#EF681827.1_Jamestown_Canyon_virus_isolate_4148-03_segment_S_nucleocapsid_protei .......A.. .........A .....T.... .......... .......... .....T.... .......... ..........  [240]
#EF681828.1_Jamestown_Canyon_virus_isolate_4473-00_segment_S_nucleocapsid_protei .......A.. .........A .....T.... .......... .......... .....T.... .......... ..........  [240]
#EF681829.1_Jamestown_Canyon_virus_isolate_4742-04_segment_S_nucleocapsid_protei .......A.. .........A .....T.... .A........ .......... ..A..T.... .......... ..........  [240]
#EF681830.1_Jamestown_Canyon_virus_isolate_2274-05_segment_S_nucleocapsid_protei .......A.. .........A .....T.... .A........ .......... ..A..T.... .......... ..........  [240]
#EF681831.1_Jamestown_Canyon_virus_isolate_1472-05_segment_S_nucleocapsid_protei .......A.. .........A .....T.... .A........ .......... ..A..T.... .......... ..........  [240]
#EF681832.1_Jamestown_Canyon_virus_isolate_4910-02_segment_S_nucleocapsid_protei .......A.. .........A .....T.... .......... .......... .....T.... .......... ......G...  [240]
#EF681833.1_Jamestown_Canyon_virus_isolate_275-01_segment_S_nucleocapsid_protein .......A.. .........A .....T.... .A........ .......... ..A..T.... .......... ..........  [240]
#EF681834.1_Jamestown_Canyon_virus_isolate_339-05_segment_S_nucleocapsid_protein .......A.. .........A .....T.... .A........ .......... ..A..T.... .......... ..........  [240]
#EF681835.1_Jamestown_Canyon_virus_isolate_3836-05_segment_S_nucleocapsid_protei .......A.. .........A .....T.... .A........ .......... ..A..T.... .......... ..........  [240]
#EF681836.1_Jamestown_Canyon_virus_isolate_2286-00_segment_S_nucleocapsid_protei .......A.. .........A .....T.... .......... .......... .....T.... .......... ......G...  [240]
#EF681837.1_Jamestown_Canyon_virus_isolate_1044-05_segment_S_nucleocapsid_protei .......A.. .........A .....T.... .A........ .......... ..A..T.... .......... ..........  [240]
#EF681838.1_Jamestown_Canyon_virus_isolate_978-99_segment_S_nucleocapsid_protein .......A.. .........A .....T.... .A........ .......... ..A..T.... .......... ..........  [240]
#EF681839.1_Jamestown_Canyon_virus_isolate_4832-01_segment_S_nucleocapsid_protei .......A.. .........A .....T.... .A........ .......... ..A..T.... .......... ..........  [240]
#EF681841.1_Jamestown_Canyon_virus_isolate_7101-03_segment_S_nucleocapsid_protei .......A.. .........A .....T.... .A........ .......... ..A..T.... .......... ..........  [240]
#EF681842.1_Jamestown_Canyon_virus_isolate_Simsbury_segment_S_nucleocapsid_prote .......A.. .........A .....T.... .A........ .......... ..A..T.... .......... ..........  [240]
#EF681843.1_Jamestown_Canyon_virus_isolate_8011-03_segment_S_nucleocapsid_protei .......A.. .........A .....T.... .A........ .......... ..A..T.... .......... ..........  [240]
#EF681844.1_Jamestown_Canyon_virus_isolate_8536-03_segment_S_nucleocapsid_protei .......A.. .........A .....T.... .......... .......... .....T.... .......... ......G...  [240]
#EF681845.1_Jamestown_Canyon_virus_isolate_11497-03_segment_S_nucleocapsid_prote .......A.. .........A .....T.... .A........ .......... ..A..T.... .......... ..........  [240]
#EF681846.1_Jamestown_Canyon_virus_isolate_13995-03_segment_S_nucleocapsid_prote .......A.. .........A .....T.... .A........ .......... ..A..T.... .......... ..........  [240]
#EF681847.1_Jamestown_Canyon_virus_isolate_1768-98_segment_S_nucleocapsid_protei .......A.. .........A .....T.... .......... .......... .....T.... .......... ......G...  [240]
#EF681848.1_Jamestown_Canyon_virus_isolate_1385-06_segment_S_nucleocapsid_protei .......A.. .........A .....T.... .A........ .......... ..A..T.... .......... ..........  [240]
```

```
#EF681849.1_Jamestown_Canyon_virus_isolate_2989-06_segment_S_nucleocapsid_protei .......A.. .........A .....T.... .A........ .......... ..A..T.... .......... ..........  [240]
#EF681850.1_Jamestown_Canyon_virus_isolate_3381-06_segment_S_nucleocapsid_protei .......A.. .........A .....T.... .A........ .......... ..A..T.... .......... ..........  [240]
#EF681851.1_Jamestown_Canyon_virus_isolate_4095-06_segment_S_nucleocapsid_protei .......A.. .........A .....T.... .A........ .......... ..A..T.... .......... ..........  [240]
#EF681852.1_Jamestown_Canyon_virus_isolate_4078-06_segment_S_nucleocapsid_protei .......A.. .........A .....T.... .A........ .......... ..A..T.... .......... ..........  [240]
#EF681853.1_Jamestown_Canyon_virus_isolate_11-92_segment_S_nucleocapsid_protein_ .......A.. .........A .....T.... .......... .......... .....T.... .......... ......G...  [240]
#EF681854.1_Jamestown_Canyon_virus_isolate_23-97_segment_S_nucleocapsid_protein_ .......A.. .........A .....T.... .......... .......... .....T.... .......... ......G...  [240]
#EF681855.1_Jamestown_Canyon_virus_isolate_25-97_segment_S_nucleocapsid_protein_ .......A.. .........A .....T.... .A........ .......... ..A..T.... .......... ..........  [240]
#EF681856.1_Jamestown_Canyon_virus_isolate_29-97_segment_S_nucleocapsid_protein_ .......A.. .........A .....T.... .......... .......... .....T.... .......... ......G...  [240]
#EF681857.1_Jamestown_Canyon_virus_isolate_423-99_segment_S_nucleocapsid_protein .......A.. .........A .....T.... .A........ .......... ..A..T.... .......... ..........  [240]
#EF681858.1_Jamestown_Canyon_virus_isolate_1262-98_segment_S_nucleocapsid_protei .......A.. .........A .....T.... .......... .......... .....T.... .......... ......G...  [240]
#EF681859.1_Jamestown_Canyon_virus_isolate_3438-06_segment_S_nucleocapsid_protei .......A.. .........A .....T.... .A........ .......... ..A..T.... .......... ..........  [240]
#HM007350.1_Jamestown_Canyon_virus_strain_61V2235_nucleoprotein_and_NSs_protein_ .......A.. .........A .....T.... .......... .......... .......... .......... .T........  [240]
#HM007353.1_Jamestown_Canyon_virus_strain_3573-03_nucleoprotein_gene_complete_cd .......A.. .........A .....T.... .......... .......... .....T.... .......... ......G...  [240]
#HM007356.1_Jamestown_Canyon_virus_strain_3324-04_nucleoprotein_and_NSs_protein_ .......A.. .........A .....T.... .A........ .......... ..A..T.... .......... ..........  [240]
#KM215518.1_Jamestown_Canyon_virus_isolate_F1819_nucleocapsid_protein_and_nonstr .......A.. .........A .....T.... .A........ .......... ..A..T.... .......... ..........  [240]
#KM215519.1_Jamestown_Canyon_virus_isolate_F6626_nucleocapsid_protein_and_nonstr .......A.. .........A .....T.... .A........ .......... ..A..T.... .......... ..........  [240]
#KM215520.1_Jamestown_Canyon_virus_isolate_ND0283_nucleocapsid_protein_and_nonst .......A.. .........A .....T.... .A........ .......... ..A..T.... .......... ..........  [240]
#KM215521.1_Jamestown_Canyon_virus_isolate_ND6194_nucleocapsid_protein_and_nonst .......A.. .........A .....T.... .A........ .......... ..A..T.... .......... ..........  [240]
#KM215522.1_Jamestown_Canyon_virus_isolate_W6701_nucleocapsid_protein_and_nonstr .......A.. .........A .....T.... .A........ .......... ..A..T.... .......... ..........  [240]
#KM215523.1_Jamestown_Canyon_virus_isolate_W14530_nucleocapsid_protein_and_nonst .......A.. .........A .....T.... .A........ .......... ..A..T.... .......... ..........  [240]
#KM215524.1_Jamestown_Canyon_virus_isolate_W16690_nucleocapsid_protein_and_nonst .......A.. .........A .....T.... .A........ .......... ..A..T.... .......... ..........  [240]
#KM215525.1_Jamestown_Canyon_virus_isolate_W18699_nucleocapsid_protein_and_nonst .......A.. .........A .....T.... .......... .......... ......A... .......... ......G...  [240]
#KM215526.1_Jamestown_Canyon_virus_isolate_W15316_nucleocapsid_protein_and_nonst .......A.. .........A .....T.... .A........ .......... ..A..T.... .......... ..........  [240]
#KM215527.1_Jamestown_Canyon_virus_isolate_W17680_nucleocapsid_protein_and_nonst .......A.. .........A .....T.... .A........ .......... ..A..T.... .......... ..........  [240]
#KM215528.1_Jamestown_Canyon_virus_isolate_W19543_nucleocapsid_protein_and_nonst .......A.. .........A .....T.... .A........ .......... ..A..T.... .......... ..........  [240]
#KM215529.1_Jamestown_Canyon_virus_isolate_W19925_nucleocapsid_protein_and_nonst .......A.. .........A .....T.... .A........ .......... ..A..T.... .......... ..........  [240]
#KM215530.1_Jamestown_Canyon_virus_isolate_W20764_nucleocapsid_protein_and_nonst .......A.. .........A .....T.... .A........ .......... ..A..T.... .......... ..........  [240]
#KM215531.1_Jamestown_Canyon_virus_isolate_W22352_nucleocapsid_protein_and_nonst .......A.. .........A .....T.... .A........ .......... ..A..T.... .......... ..........  [240]
#KM215532.1_Jamestown_Canyon_virus_isolate_F1829_nucleocapsid_protein_and_nonstr .......A.. .........A .....T.... .A........ .......... ..A..T.... .......... ..........  [240]
#KM215533.1_Jamestown_Canyon_virus_isolate_F6228_nucleocapsid_protein_and_nonstr .......A.. .........A .....T.... .A........ .......... ..A..T.... .......... ..........  [240]
#KM215534.1_Jamestown_Canyon_virus_isolate_F6235_nucleocapsid_protein_and_nonstr .......A.. .........A .....T.... .A........ .......... ..A..T.... .......... ..........  [240]
#KM215535.1_Jamestown_Canyon_virus_isolate_F10095_nucleocapsid_protein_and_nonst .......A.. .........A .....T.... .A........ .......... ..A..T.... .......... ..........  [240]
#KM215536.1_Jamestown_Canyon_virus_isolate_F13418_nucleocapsid_protein_and_nonst .......A.. .........A .....T.... .A........ .......... ..A..T.... .......... ..........  [240]
#KM215537.1_Jamestown_Canyon_virus_isolate_F14162_nucleocapsid_protein_and_nonst .......A.. .........A .....T.... .A........ .......... ..A..T.... .......... ..........  [240]
#KM215538.1_Jamestown_Canyon_virus_isolate_F14183_nucleocapsid_protein_and_nonst .......A.. .........A .....T.... .A........ .......... ..A..T.... .......... ..........  [240]
#KM215539.1_Jamestown_Canyon_virus_isolate_F14278_nucleocapsid_protein_and_nonst .......A.. .........A .....T.... .A........ .......... ..A..T.... .......... ..........  [240]
#KM215540.1_Jamestown_Canyon_virus_isolate_F16109_nucleocapsid_protein_and_nonst .......A.. .........A .....T.... .A........ .......... ..A..T.... .......... ..........  [240]
#KM215541.1_Jamestown_Canyon_virus_isolate_W8270_nucleocapsid_protein_and_nonstr .......A.. .........A .....T.... .A........ .......... ..A..T.... .......... ..........  [240]
#KM215542.1_Jamestown_Canyon_virus_isolate_W16455_nucleocapsid_protein_and_nonst .......A.. .........A .....T.... .A........ .......... ..A..T.... .......... ..........  [240]
#KM215543.1_Jamestown_Canyon_virus_isolate_W18738_nucleocapsid_protein_and_nonst .......A.. .........A .....T.... .A........ .......... ..A..T.... .......... ..........  [240]
#KM215544.1_Jamestown_Canyon_virus_isolate_F4276_nucleocapsid_protein_and_nonstr .......A.. .........A .....T.... .A........ .......... ..A..T.... .......... ..........  [240]
#KM215545.1_Jamestown_Canyon_virus_isolate_W19962_nucleocapsid_protein_and_nonst .......A.. .........A .....T.... .A........ .......... ..A..T.... .......... ..........  [240]
#KM215546.1_Jamestown_Canyon_virus_isolate_F5609_nucleocapsid_protein_and_nonstr .......A.. .........A .....T.... .A........ .......... ..A..T.... .......... ..........  [240]
#KM215547.1_Jamestown_Canyon_virus_isolate_F7826_nucleocapsid_protein_and_nonstr .......A.. .........A .....T.... .A........ .......... ..A..T.... .......... ..........  [240]
#KM215548.1_Jamestown_Canyon_virus_isolate_F7932_nucleocapsid_protein_and_nonstr .......A.. .........A .....T.... .A........ .......... ..A..T.... .......... ..........  [240]
#KM215549.1_Jamestown_Canyon_virus_isolate_F8532_nucleocapsid_protein_and_nonstr .......A.. .........A .....T.... .A........ .......... ..A..T.... .......... ..........  [240]
#KM215550.1_Jamestown_Canyon_virus_isolate_F10720_nucleocapsid_protein_and_nonst .......A.. .........A .....T.... .A........ .......... ..A..T.... .......... ..........  [240]
#KM215551.1_Jamestown_Canyon_virus_isolate_ND1300_nucleocapsid_protein_and_nonst .......A.. .........A .....T.... .A........ .......... ..A..T.... .......... ..........  [240]
#KM215552.1_Jamestown_Canyon_virus_isolate_W13135_nucleocapsid_protein_and_nonst .......A.. .........A .....T.... .A........ .......... ..A..T.... .......... ..........  [240]
#KM215553.1_Jamestown_Canyon_virus_isolate_W16456_nucleocapsid_protein_and_nonst .......A.. .........A .....T.... .A........ .......... ..A..T.... .......... ..........  [240]
#KM215554.1_Jamestown_Canyon_virus_isolate_W16590_nucleocapsid_protein_and_nonst .......A.. .........A .....T.... .A........ .......... ..A..T.... .......... ..........  [240]
#KM215555.1_Jamestown_Canyon_virus_isolate_W19651_nucleocapsid_protein_and_nonst .......A.. .........A .....T.... .A........ .......... ..A..T.... .......... ..........  [240]
#KM215556.1_Jamestown_Canyon_virus_isolate_F3406_nucleocapsid_protein_and_nonstr .......A.. .........A .....T.... .A........ .......... ..A..T.... .......... ..........  [240]
#KM215557.1_Jamestown_Canyon_virus_isolate_F6577_nucleocapsid_protein_and_nonstr .......A.. .........A .....T.... .A........ .......... ..A..T.... .......... ..........  [240]
#KM215558.1_Jamestown_Canyon_virus_isolate_ND374_nucleocapsid_protein_and_nonstr .......A.. .........A .....T.... .A........ .......... ..A..T.... .......... ..........  [240]
#KM215559.1_Jamestown_Canyon_virus_isolate_W18393_nucleocapsid_protein_and_nonst .......A.. .........A .....T.... .......... .......... ......A... .......... ......G...  [240]
#KM215560.1_Jamestown_Canyon_virus_isolate_W18901_nucleocapsid_protein_and_nonst .......A.. .........A .....T.... .......... .......... ......A... .......... ......G...  [240]
```

```
#KX817317.1_Jamestown_Canyon_virus_strain_61V2235_segment_S_complete_sequence    .......A.. .........A .....T.... .......... .......... .......... .......... .T........  [240]
#MH370817.1_Jamestown_Canyon_virus_isolate_L36708_segment_S_complete_sequence    .......A.. .........A .....T.... .......... .......... .....T.... .......... ......G...  [240]
#MH370820.1_Jamestown_Canyon_virus_isolate_MN256-260_segment_S_complete_sequence .......A.. .........A .....T.... .A........ .......... ..A..T.... .......... ..........  [240]
#U12799.1_Jamestown_Canyon_virus_DAV28_S_RNA_segment_N_and_NSs_protein_genes_com .......A.. .........A .....T.... .......... .......... .......... .......... .T........  [240]
#U12796.1_Jamestown_Canyon_virus_61v2235_S_RNA_segment_N_and_NSs_protein_genes_c .......A.. .........A .....T.... .......... .......... .......... .......... .T........  [240]
#KM215561.1_Jamestown_Canyon_virus_isolate_W23697_nucleocapsid_protein_and_nonst .......A.. .........A .....T.... .A........ .......... ..A..T.... .......... ..........  [240]
#KT288271.1_Inkoo_virus_strain_LEIV-15248Iv_segment_S_nucleoprotein_(N)_gene_com .......A.. .........A .....T.... .A........ .......... .....T.... .......... ..........  [240]
#KT288274.1_Inkoo_virus_strain_LEIV-18154Yak_segment_S_nucleoprotein_(N)_gene_co .......A.. .........A .....T.... .A........ .......... .....T.... .......... ..........  [240]
#KT288275.1_Inkoo_virus_strain_LEIV-9874Kar_segment_S_nucleoprotein_(N)_gene_com .......A.. .........A .....T.... .A........ .......... .....T.... .......... ..........  [240]
#KT288277.1_Inkoo_virus_strain_LEIV-18784Yak_segment_S_nucleoprotein_(N)_gene_co .......A.. .........A .....T.... .A........ .......... .....T.... .......... ..........  [240]
#KT288280.1_Inkoo_virus_strain_LEIV-22780Tyum_segment_S_nucleoprotein_(N)_gene_c .......A.. .........A .....T.... .A........ .......... .....T.... .......... ..........  [240]
#KT288283.1_Inkoo_virus_strain_LEIV-18152Yak_segment_S_nucleoprotein_(N)_gene_co .......A.. .........A .....T.... .A........ .......... .....T.... .......... ..........  [240]
#KT288286.1_Inkoo_virus_strain_LEIV-21643Kra_segment_S_nucleoprotein_(N)_gene_co .......A.. .........A .....T.... .A........ .......... .....T.... .......... ..........  [240]
#KX554935.1_Inkoo_virus_strain_Lovanger_nucleocapsid_protein_and_nonstructural_p .......A.. .........A .....T.... .A........ .......... .....T.... .......... ..........  [240]
#U47137.1_Inkoo_virus_Prototype_KN3641_nucleocapsid_protein_and_non-structural_p .......A.. .........A .....T.... .A........ .......... .....T.... .......... ..........  [240]
#U47138.1_Inkoo_virus_SW_AR_83-161_nucleocapsid_protein_and_non-structural_prote .......A.. .........A .....T.... .A........ .......... .....T.... .......... ..........  [240]
#Z68496.1_Inkoo_virus_RNA_for_N_protein_and_RNA_for_NS_protein_strain_KN_3641    .......A.. .........A .....T.... .A........ .......... .....T.... .......... ..........  [240]
```

MELcxR273-249

```
#KT630290.1_Keystone_virus_strain_KEYV/Ochlerotatus_atlanticus/USA/KEYVLK01/2005 AGGAACAACC CAATTGGTAA CAACGATCTT ACCTTGCACC GGATTTCAGG ATATCTAGCT AGATGGGTGC TGGAGCACTT  [320]
#KT630293.1_Keystone_virus_strain_KEYV/Ochlerotatus_atlanticus/USA/KEYVLK02/2005 .......... .......... .......... .......... .......G.. .......... .......... ..........  [320]
#MH016786.1_Keystone_virus_strain_KEYV/Homo_sapiens/Gainesville-1/2016_nucleopro .......... .......... .......... .......... .......... .......... .......... ..........  [320]
#KX817323.1_Keystone_virus_strain_B64-5587.05_segment_S_complete_sequence         .......... .......... .......... .......... .......... .......... .......... ..........  [320]
#MG821231.1_Keystone_virus_isolate_AR14033_segment_S_complete_sequence            .......... .......... .......... .......... ..G....... .......... .........T ....A.....  [320]
#MG765471.1_Keystone_virus_isolate_AVA1709441_nucleocapsid_and_NSs_genes_complet .......... .......... .......... .......... ..G....... .......... .........T ....A.....  [320]
#KX817329.1_Melao_virus_strain_TRVL_9375_segment_S_complete_sequence              .......... .......... .......... ...A.C..T. ..C....... ......T..C .......... .A..T.TG..  [320]
#KX817335.1_Serra_do_Navio_virus_strain_BeAr_103645_segment_S_complete_sequence   .......... .......... .......... ...A.C...A .A..C..... ....T..... C.C...ACAT .A..A.....  [320]
#KX817320.1_Jerry_Slough_virus_strain_BFS_4474_segment_S_complete_sequence        .......... .......... .......... ...A.C..TA ..C....... .........C ........T. .T.....T..  [320]
#KX817338.1_South_River_virus_strain_NJO-94F_segment_S_complete_sequence          .......... .......... .......... ...A.C.... ..C....T.. ....T..... ........T. .T.....T..  [320]
#GU018050.2_South_River_virus_isolate_SORV-252_nucleoprotein_and_NSs_protein_gen .......... .......... .......... ...A.C..T. ..C....C.. .........C ........T. .T.....T..  [320]
#EF681804.1_Jamestown_Canyon_virus_isolate_5592-02_segment_S_nucleocapsid_protei .......G.. .......... .......... ...A.C..T. ..C....... .........C ........T. .T.....T..  [320]
#EF681805.1_Jamestown_Canyon_virus_isolate_368-99_segment_S_nucleocapsid_protein .......... .......... .......... ...A.C..T. ..C....... .........C ........T. .T.....T..  [320]
#EF681806.1_Jamestown_Canyon_virus_isolate_6163-03_segment_S_nucleocapsid_protei .......... .......... .......... ...A.C..T. ..C....... .........C ........T. .T.....T..  [320]
#EF681807.1_Jamestown_Canyon_virus_isolate_468-04_segment_S_nucleocapsid_protein .......... .......... .......... ...A.C..T. ..C....C.. .........C ........T. .T.....T..  [320]
#EF681808.1_Jamestown_Canyon_virus_isolate_2179-00_segment_S_nucleocapsid_protei .......... .......... .......... ...A.C..T. ..C....... .........C ........T. .T.....T..  [320]
#EF681809.1_Jamestown_Canyon_virus_isolate_779-98_segment_S_nucleocapsid_protein .......... .......... .......... ...A.C..T. ..C....C.. .........C ........T. .T.....T..  [320]
#EF681810.1_Jamestown_Canyon_virus_isolate_810-98_segment_S_nucleocapsid_protein .......... .......... .......... ...A.C..T. ..C....C.. .........C ........T. .T.....T..  [320]
#EF681811.1_Jamestown_Canyon_virus_isolate_811-00_segment_S_nucleocapsid_protein .......... .......... .......... ...A.C..T. ..C....... .........C ........T. .T.....T..  [320]
#EF681812.1_Jamestown_Canyon_virus_isolate_1697-03_segment_S_nucleocapsid_protei .......... .......... .......... ...A.C..T. ..C....C.. .........C ........T. .T.....T..  [320]
#EF681813.1_Jamestown_Canyon_virus_isolate_1425-02_segment_S_nucleocapsid_protei .......... .......... .......... ...A.C..T. ..C....C.. .........C ........T. .T.....T..  [320]
#EF681814.1_Jamestown_Canyon_virus_isolate_1441-04_segment_S_nucleocapsid_protei .......... .......... .......... ...A.C..T. ..C....C.. .........C ........T. .T.....T..  [320]
#EF681815.1_Jamestown_Canyon_virus_isolate_928-00_segment_S_nucleocapsid_protein .......... .......... .......... ...A.C..T. ..C....C.. .........C ........T. .T.....T..  [320]
#EF681816.1_Jamestown_Canyon_virus_isolate_1064-03_segment_S_nucleocapsid_protei .......... .......... .......... ...A.C..T. ..C....C.. ....T....C ........T. .T..A..T..  [320]
#EF681817.1_Jamestown_Canyon_virus_isolate_1369-02_segment_S_nucleocapsid_protei .......... .......... .......... ...A.C..T. ..C....... .........C ........T. .T.....T..  [320]
#EF681818.1_Jamestown_Canyon_virus_isolate_1627-04_segment_S_nucleocapsid_protei .......... .......... .......... ...A.C..T. ..C....... .........C ........T. .T.....T..  [320]
#EF681819.1_Jamestown_Canyon_virus_isolate_1810-02_segment_S_nucleocapsid_protei .......... .......... .......... ...A.C..T. ..C....... .........C ........T. .T.....T..  [320]
#EF681820.1_Jamestown_Canyon_virus_isolate_2384-98_segment_S_nucleocapsid_protei .......... .......... .......... ...A.C..T. ..C....... .........C ........T. .T.....T..  [320]
#EF681821.1_Jamestown_Canyon_virus_isolate_2707-01_segment_S_nucleocapsid_protei .......... .......... .......... ...A.C..T. ..C....C.. .........C ........T. .T.....T..  [320]
#EF681822.1_Jamestown_Canyon_virus_isolate_2718-01_segment_S_nucleocapsid_protei .......... .......... .......... ...A.C..T. ..C....... .........C ........T. .T.....T..  [320]
#EF681823.1_Jamestown_Canyon_virus_isolate_3280-03_segment_S_nucleocapsid_protei .......... .......... .......... ...A.C..T. ..C....C.. .........C ........T. .T.....T..  [320]
#EF681824.1_Jamestown_Canyon_virus_isolate_3324-04_segment_S_nucleocapsid_protei .......... .......... .......... ...A.C..T. ..C....... .........C ........T. .T.....T..  [320]
#EF681825.1_Jamestown_Canyon_virus_isolate_3573-03_segment_S_nucleocapsid_protei .......... .......... .......... ...A.C..T. ..C....C.. .........C ........T. .T.....T..  [320]
#EF681826.1_Jamestown_Canyon_virus_isolate_3682-00_segment_S_nucleocapsid_protei .......... .......... .......... ...A.C..T. ..C....C.. .........C ........T. .T.....T..  [320]
#EF681827.1_Jamestown_Canyon_virus_isolate_4148-03_segment_S_nucleocapsid_protei .......... .......... .......... ...A.C..T. ..C....T.. ....T....C ........T. .T..A..T..  [320]
#EF681828.1_Jamestown_Canyon_virus_isolate_4473-00_segment_S_nucleocapsid_protei .......... .......... .......... ...A.C..T. ..C....T.. ....T....C ........T. .T..A..T..  [320]
#EF681829.1_Jamestown_Canyon_virus_isolate_4742-04_segment_S_nucleocapsid_protei .......... .......... .......... ...A.C..T. ..C....... ....T....C ........T. .T.....T..  [320]
#EF681830.1_Jamestown_Canyon_virus_isolate_2274-05_segment_S_nucleocapsid_protei .......... .......... .......... ...A.C..T. ..C....... .......... ........T. .T.....T..  [320]
#EF681831.1_Jamestown_Canyon_virus_isolate_1472-05_segment_S_nucleocapsid_protei .......... .......... .......... ...A.C..T. ..C....... .........C ........T. .T.....T..  [320]
#EF681832.1_Jamestown_Canyon_virus_isolate_4910-02_segment_S_nucleocapsid_protei .......... .......... .......... ...A.C..T. ..C....C.. .........C ........T. .T.....T..  [320]
#EF681833.1_Jamestown_Canyon_virus_isolate_275-01_segment_S_nucleocapsid_protein .......... .......... .......... ...A.C..T. ..C....... .........C ........T. .T.....T..  [320]
#EF681834.1_Jamestown_Canyon_virus_isolate_339-05_segment_S_nucleocapsid_protein .......... .......... .......... ...A.C..T. ..C....... .........C ........T. .T.....T..  [320]
#EF681835.1_Jamestown_Canyon_virus_isolate_3836-05_segment_S_nucleocapsid_protei .......... .......... .......... ...A.C..T. ..C....... .........C ........T. .T.....T..  [320]
#EF681836.1_Jamestown_Canyon_virus_isolate_2286-00_segment_S_nucleocapsid_protei .......... .......... .......... ...A.C..T. ..C....C.. .........C ........T. .T.....T..  [320]
#EF681837.1_Jamestown_Canyon_virus_isolate_1044-05_segment_S_nucleocapsid_protei .......... .......... .......... ...A.C..T. ..C....... .........C ........T. .T.....T..  [320]
#EF681838.1_Jamestown_Canyon_virus_isolate_978-99_segment_S_nucleocapsid_protein .......... .......... .......... ...A.C..T. ..C....... .........C ........T. .T.....T..  [320]
#EF681839.1_Jamestown_Canyon_virus_isolate_4832-01_segment_S_nucleocapsid_protei .......... .......... .......... ...A.C..T. ..C....... .........C ........T. .T.....T..  [320]
#EF681841.1_Jamestown_Canyon_virus_isolate_7101-03_segment_S_nucleocapsid_protei .......... .......... .......... ...A.C..T. ..C....... .........C ........T. .T.....T..  [320]
#EF681842.1_Jamestown_Canyon_virus_isolate_Simsbury_segment_S_nucleocapsid_prote .......... .......... .......... ...A.C..T. ..C....... .........C ........T. .T.....T..  [320]
#EF681843.1_Jamestown_Canyon_virus_isolate_8011-03_segment_S_nucleocapsid_protei .......... .......... .......... ...A.C..T. ..C....... .........C ........T. .T.....T..  [320]
#EF681844.1_Jamestown_Canyon_virus_isolate_8536-03_segment_S_nucleocapsid_protei .......... .......... .......... ...A.C..T. ..C....C.. .........C ........T. .T.....T..  [320]
#EF681845.1_Jamestown_Canyon_virus_isolate_11497-03_segment_S_nucleocapsid_prote .......... .......... .......... ...A.C..T. ..C....... .........C ........T. .T.....T..  [320]
#EF681846.1_Jamestown_Canyon_virus_isolate_13995-03_segment_S_nucleocapsid_prote .......... .......... .......... ...A.C..T. ..C....... .........C ........T. .T.....T..  [320]
#EF681847.1_Jamestown_Canyon_virus_isolate_1768-98_segment_S_nucleocapsid_protei .......... .......... .......... ...A.C..T. ..C....C.. .........C ........T. .T.....T..  [320]
#EF681848.1_Jamestown_Canyon_virus_isolate_1385-06_segment_S_nucleocapsid_protei .......... .......... .......... ...A.C..T. ..C....... .........C ........T. .T.....T..  [320]
```

```
#EF681849.1_Jamestown_Canyon_virus_isolate_2989-06_segment_S_nucleocapsid_protei .......... .......... .......... ...A.C..T. ..C....... .........C ........T. .T.....T..  [320]
#EF681850.1_Jamestown_Canyon_virus_isolate_3381-06_segment_S_nucleocapsid_protei .......... .......... .......... ...A.C..T. ..C....... .........C ........T. .T.....T..  [320]
#EF681851.1_Jamestown_Canyon_virus_isolate_4095-06_segment_S_nucleocapsid_protei .......... .......... .......... ...A.C..T. ..C....... .........C ........T. .T.....T..  [320]
#EF681852.1_Jamestown_Canyon_virus_isolate_4078-06_segment_S_nucleocapsid_protei .......... .......... .......... ...A.C..T. ..C....... .........C ........T. .T.....T..  [320]
#EF681853.1_Jamestown_Canyon_virus_isolate_11-92_segment_S_nucleocapsid_protein_ .......... .......... .......... ...A.C..T. ..C....C.. .........C ........T. .T.....T..  [320]
#EF681854.1_Jamestown_Canyon_virus_isolate_23-97_segment_S_nucleocapsid_protein_ .......... .......... .......... ...A.C..T. ..C....C.. .........C ........T. .T.....T..  [320]
#EF681855.1_Jamestown_Canyon_virus_isolate_25-97_segment_S_nucleocapsid_protein_ .......... .......... .......... ...A.C..T. ..C....... .........C ........T. .T.....T..  [320]
#EF681856.1_Jamestown_Canyon_virus_isolate_29-97_segment_S_nucleocapsid_protein_ .......... .......... .......... ...A.C..T. ..C....C.. .........C ........T. .T.....T..  [320]
#EF681857.1_Jamestown_Canyon_virus_isolate_423-99_segment_S_nucleocapsid_protein .......... .......... .......... ...A.C..T. ..C....... .........C ........T. .T.....T..  [320]
#EF681858.1_Jamestown_Canyon_virus_isolate_1262-98_segment_S_nucleocapsid_protei .......... .......... .......... ...A.C..T. ..C....C.. .........C ........T. .T.....T..  [320]
#EF681859.1_Jamestown_Canyon_virus_isolate_3438-06_segment_S_nucleocapsid_protei .......... .......... .......... ...A.C..T. ..C....... .........C ........T. .T.....T..  [320]
#HM007350.1_Jamestown_Canyon_virus_strain_61V2235_nucleoprotein_and_NSs_protein_ .......... .......... .......... ...A.C..TA ..C....... .........C ........T. .T.....T..  [320]
#HM007353.1_Jamestown_Canyon_virus_strain_3573-03_nucleoprotein_gene_complete_cd .......... .......... .......... ...A.C..T. ..C....C.. .........C ........T. .T.....T..  [320]
#HM007356.1_Jamestown_Canyon_virus_strain_3324-04_nucleoprotein_and_NSs_protein_ .......... .......... .......... ...A.C..T. ..C....... .........C ........T. .T.....T..  [320]
#KM215518.1_Jamestown_Canyon_virus_isolate_F1819_nucleocapsid_protein_and_nonstr .......... .......... .........C ...A.C..T. ..C....... .........C ........T. .T.....T..  [320]
#KM215519.1_Jamestown_Canyon_virus_isolate_F6626_nucleocapsid_protein_and_nonstr .......... .......... .........C ...A.C..T. ..C....... .........C ........T. .T.....T..  [320]
#KM215520.1_Jamestown_Canyon_virus_isolate_ND0283_nucleocapsid_protein_and_nonst .......... .......... .......... ...A.C..T. ..C....... .........C ........T. .T.....T..  [320]
#KM215521.1_Jamestown_Canyon_virus_isolate_ND6194_nucleocapsid_protein_and_nonst .......... .......... .......... ...A.C..T. ..C....... .........  ........T. .T.....T..  [320]
#KM215522.1_Jamestown_Canyon_virus_isolate_W6701_nucleocapsid_protein_and_nonstr .......... .......... .......... ...A.C..T. ..C....... .........C ........T. .T.....T..  [320]
#KM215523.1_Jamestown_Canyon_virus_isolate_W14530_nucleocapsid_protein_and_nonst .......... .......... .......... ...A.C..T. ..C....... .........C ........T. .T.....T..  [320]
#KM215524.1_Jamestown_Canyon_virus_isolate_W16690_nucleocapsid_protein_and_nonst .......... .......... .......... ...A.C..TA ..C....... .........C ........T. .T.....T..  [320]
#KM215525.1_Jamestown_Canyon_virus_isolate_W18699_nucleocapsid_protein_and_nonst .......... .......... .......... ...A.C..TA ..C....... ....T....C ........T. .T.....T..  [320]
#KM215526.1_Jamestown_Canyon_virus_isolate_W15316_nucleocapsid_protein_and_nonst .......... .......... .......... ...A.C..T. ..C....... .........C ........T. .T.....T..  [320]
#KM215527.1_Jamestown_Canyon_virus_isolate_W17680_nucleocapsid_protein_and_nonst .......... .......... .......... ...A.C..T. ..C....... .........C ........T. .T.....T..  [320]
#KM215528.1_Jamestown_Canyon_virus_isolate_W19543_nucleocapsid_protein_and_nonst .......... .......... .......... ...A.C..T. ..C....... .........C ........T. .T.....T..  [320]
#KM215529.1_Jamestown_Canyon_virus_isolate_W19925_nucleocapsid_protein_and_nonst .......... .......... .......... ...A.C..T. ..C....... .........C ........T. .T.....T..  [320]
#KM215530.1_Jamestown_Canyon_virus_isolate_W20764_nucleocapsid_protein_and_nonst .......... .......... .......... ...A.C..T. ..C....... .........C ........T. .T.....T..  [320]
#KM215531.1_Jamestown_Canyon_virus_isolate_W22352_nucleocapsid_protein_and_nonst .......... .......... .......... ...A.C..T. ..C....... .........C ........T. .T..A..T..  [320]
#KM215532.1_Jamestown_Canyon_virus_isolate_F1829_nucleocapsid_protein_and_nonstr .......... .......... .........C ...A.C..T. ..C....... .........C ........T. .T.....T..  [320]
#KM215533.1_Jamestown_Canyon_virus_isolate_F6228_nucleocapsid_protein_and_nonstr .......... .......... .........C ...A.C..T. ..C....... .........C ........T. .T.....T..  [320]
#KM215534.1_Jamestown_Canyon_virus_isolate_F6235_nucleocapsid_protein_and_nonstr .......... .......... .........C ...A.C..T. ..C....... .........C ........T. .T.....T..  [320]
#KM215535.1_Jamestown_Canyon_virus_isolate_F10095_nucleocapsid_protein_and_nonst .......... .......... .......... ...A.C..T. ..C....... .........C ........T. .T.....T..  [320]
#KM215536.1_Jamestown_Canyon_virus_isolate_F13418_nucleocapsid_protein_and_nonst .......... .......... .......... ...A.C..T. ..C....... .........C ........T. .T.....T..  [320]
#KM215537.1_Jamestown_Canyon_virus_isolate_F14162_nucleocapsid_protein_and_nonst .......... .......... .......... ...A.C..TA ..C....... .........C ........T. .T.....T..  [320]
#KM215538.1_Jamestown_Canyon_virus_isolate_F14183_nucleocapsid_protein_and_nonst .......... .......... .......... ...A.C..TA ..C....... .........C ........T. .T.....T..  [320]
#KM215539.1_Jamestown_Canyon_virus_isolate_F14278_nucleocapsid_protein_and_nonst .......... .......... .......... ...A.C..T. ..C....... .........  ........T. .T.....T..  [320]
#KM215540.1_Jamestown_Canyon_virus_isolate_F16109_nucleocapsid_protein_and_nonst .......... .......... .......... ...A.C..T. ..C....... .........C ........T. .T.....T..  [320]
#KM215541.1_Jamestown_Canyon_virus_isolate_W8270_nucleocapsid_protein_and_nonstr .......... .......... .........C ...A.C..T. ..C....... .........C ........T. .T.....T..  [320]
#KM215542.1_Jamestown_Canyon_virus_isolate_W16455_nucleocapsid_protein_and_nonst .......... .......... .......... ...A.C..TA ..C....... .........C ........T. .T.....T..  [320]
#KM215543.1_Jamestown_Canyon_virus_isolate_W18738_nucleocapsid_protein_and_nonst .......... .......... .......... ...A.C..T. ..C....... .........C ........T. .T.....T..  [320]
#KM215544.1_Jamestown_Canyon_virus_isolate_F4276_nucleocapsid_protein_and_nonstr .......... .......... .......... ...A.C..T. ..C....... .........C ........T. .T.....T..  [320]
#KM215545.1_Jamestown_Canyon_virus_isolate_W19962_nucleocapsid_protein_and_nonst .......... .......... .......... ...A.C..T. ..C....... .........C ........T. .T.....T..  [320]
#KM215546.1_Jamestown_Canyon_virus_isolate_F5609_nucleocapsid_protein_and_nonstr .......... .......... .........C ...A.C..T. ..C....... .........C ........T. .T.....T..  [320]
#KM215547.1_Jamestown_Canyon_virus_isolate_F7826_nucleocapsid_protein_and_nonstr .......... .......... .........C ...A.C..T. ..C....... .........C ........T. .T.....T..  [320]
#KM215548.1_Jamestown_Canyon_virus_isolate_F7932_nucleocapsid_protein_and_nonstr .......... .......... .......... ...A.C.... ..C....... .........C ........T. .T.....T..  [320]
#KM215549.1_Jamestown_Canyon_virus_isolate_F8532_nucleocapsid_protein_and_nonstr .......... .......... .........C ...A.C..T. ..C....... .........C ........T. .T.....T..  [320]
#KM215550.1_Jamestown_Canyon_virus_isolate_F10720_nucleocapsid_protein_and_nonst .......... .......... .........C ...A.C..T. ..C....... .........C ........T. .T.....T..  [320]
#KM215551.1_Jamestown_Canyon_virus_isolate_ND1300_nucleocapsid_protein_and_nonst .......... .......... .......... ...A.C..T. ..C....... .........C ........T. .T.....T..  [320]
#KM215552.1_Jamestown_Canyon_virus_isolate_W13135_nucleocapsid_protein_and_nonst .......... .......... .........C ...A.C..T. ..C....... .........C ........T. .T.....T..  [320]
#KM215553.1_Jamestown_Canyon_virus_isolate_W16456_nucleocapsid_protein_and_nonst .......... .......... .......... ...A.C..TA ..C....... .........C ........T. .T.....T..  [320]
#KM215554.1_Jamestown_Canyon_virus_isolate_W16590_nucleocapsid_protein_and_nonst .......... .......... .........C ...A.C..T. ..C....... .........C ........T. .T.....T..  [320]
#KM215555.1_Jamestown_Canyon_virus_isolate_W19651_nucleocapsid_protein_and_nonst .......... .......... .......... ...A.C..T. ..C....... .........C ........T. .T.....T..  [320]
#KM215556.1_Jamestown_Canyon_virus_isolate_F3406_nucleocapsid_protein_and_nonstr .......... .......... .......... ...A.C..T. ..C....... .........C ........T. .T.....T..  [320]
#KM215557.1_Jamestown_Canyon_virus_isolate_F6577_nucleocapsid_protein_and_nonstr .......... .......... .......... ...A.C..T. ..C....... .........C ........T. .T.....T..  [320]
#KM215558.1_Jamestown_Canyon_virus_isolate_ND374_nucleocapsid_protein_and_nonstr .......... .......... .......... ...A.C..T. ..C....... .........C ........T. .T.....T..  [320]
#KM215559.1_Jamestown_Canyon_virus_isolate_W18393_nucleocapsid_protein_and_nonst .......... .......... .......... ...A.C..TA ..C....... ....T....C ........T. .T.....T..  [320]
#KM215560.1_Jamestown_Canyon_virus_isolate_W18901_nucleocapsid_protein_and_nonst .......... .......... .......... ...A.C..TA ..C....... ....T....C ........T. .T.....T..  [320]
```

```
#KX817317.1_Jamestown_Canyon_virus_strain_61V2235_segment_S_complete_sequence    .......... .......... .......... ...A.C..TA ..C....... .........C ........T. .T.....T..  [320]
#MH370817.1_Jamestown_Canyon_virus_isolate_L36708_segment_S_complete_sequence    .......... .......... .....C.... ...A.C..T. ..C....C.. .........C ........T. .T.....T..  [320]
#MH370820.1_Jamestown_Canyon_virus_isolate_MN256-260_segment_S_complete_sequence .......... .......... .......... ...A.C..T. ..C....... .........C ........T. .T.....T..  [320]
#U12799.1_Jamestown_Canyon_virus_DAV28_S_RNA_segment_N_and_NSs_protein_genes_com .......... .......... .......... ...A.C..TA ..C....... .........C ........T. .T.....T..  [320]
#U12796.1_Jamestown_Canyon_virus_61v2235_S_RNA_segment_N_and_NSs_protein_genes_c .......... .......... .......... ...A.C..TA ..C....... .........C ........T. .T.....T..  [320]
#KM215561.1_Jamestown_Canyon_virus_isolate_W23697_nucleocapsid_protein_and_nonst .......... .......... .......... ...A.C..TA ..C....... .........C ........T. .T.....T..  [320]
#KT288271.1_Inkoo_virus_strain_LEIV-15248Iv_segment_S_nucleoprotein_(N)_gene_com .......... .......... .......... ...A.C.... ..C....T.. ...CT....A ........C. .T........  [320]
#KT288274.1_Inkoo_virus_strain_LEIV-18154Yak_segment_S_nucleoprotein_(N)_gene_co .......... .......... .......... ...A.C..T. ..C....T.. .........C ........C. .T.....T..  [320]
#KT288275.1_Inkoo_virus_strain_LEIV-9874Kar_segment_S_nucleoprotein_(N)_gene_com .......... .......... .......... ...A.C..T. ..C....T.. ...CT....A ........C. .T........  [320]
#KT288277.1_Inkoo_virus_strain_LEIV-18784Yak_segment_S_nucleoprotein_(N)_gene_co .......... .......... .......... ...A.C..T. ..C....T.. .........C ........C. .T.....T..  [320]
#KT288280.1_Inkoo_virus_strain_LEIV-22780Tyum_segment_S_nucleoprotein_(N)_gene_c .......... .......... .......... ...A.C..T. ..C....T.. .........C ........C. .T.....T..  [320]
#KT288283.1_Inkoo_virus_strain_LEIV-18152Yak_segment_S_nucleoprotein_(N)_gene_co .......... .......... .......... ...A.C..T. ..C....T.. .........C ........C. .T.....T..  [320]
#KT288286.1_Inkoo_virus_strain_LEIV-21643Kra_segment_S_nucleoprotein_(N)_gene_co .......... .......... .......... ...A.C.... ..C....T.. ....T....A ........C. .T.....T..  [320]
#KX554935.1_Inkoo_virus_strain_Lovanger_nucleocapsid_protein_and_nonstructural_p .......... .......... .......... ...A.C.... ..C....T.. ...CT....A ........C. .T........  [320]
#U47137.1_Inkoo_virus_Prototype_KN3641_nucleocapsid_protein_and_non-structural_p .......... .......... .......... ...A.C.... ..C....T.. ....T....A ........C. .T.....T..  [320]
#U47138.1_Inkoo_virus_SW_AR_83-161_nucleocapsid_protein_and_non-structural_prote .......... .......... .......... ...A.C.... ..C....T.. ...CT....A ........T. .T........  [320]
#Z68496.1_Inkoo_virus_RNA_for_N_protein_and_RNA_for_NS_protein_strain_KN_3641    .......... .......... .......... ...A.C.... ..C....T.. ....T....A ........C. .T.....T..  [320]
```

```
#KT630290.1_Keystone_virus_strain_KEYV/Ochlerotatus_atlanticus/USA/KEYVLK01/2005 TGGAGAGGGT GAGGACGAGT CACAAAAGGA GCTGATCAAG AGCACTGTCA TCAATCCAAT TGCCGAGTCC AATGGGATTC  [400]
#KT630293.1_Keystone_virus_strain_KEYV/Ochlerotatus_atlanticus/USA/KEYVLK02/2005 .......... .......... .......... .......... .......... .......... .......... ..........  [400]
#MH016786.1_Keystone_virus_strain_KEYV/Homo_sapiens/Gainesville-1/2016_nucleopro .......... .......... .......... .......... .......... .......... .......... ..C.......  [400]
#KX817323.1_Keystone_virus_strain_B64-5587.05_segment_S_complete_sequence        .......... .......... .......... .......... .......... .......... .......... ..........  [400]
#MG821231.1_Keystone_virus_isolate_AR14033_segment_S_complete_sequence           ...G..A... .......... ....G..... .......... ..T..C.... ....C..G.. ...T..A..T ..........  [400]
#MG765471.1_Keystone_virus_isolate_AVA1709441_nucleocapsid_and_NSs_genes_complet ...G..A... .......... ....G..... .......... ..T..C.... ....C..G.. ...T..A..T ..........  [400]
#KX817329.1_Melao_virus_strain_TRVL_9375_segment_S_complete_sequence             CAA...AAA. ..A..T..A. .C..G..... AT.A...C.. .....CA... .......T.. ...T...... ........A.  [400]
#KX817335.1_Serra_do_Navio_virus_strain_BeAr_103645_segment_S_complete_sequence  CC.T.....A .....T..AG .T..G..A.. A..A..TCG. .CA..AA... .T..C..T.. ...A..A... .....A..A.  [400]
#KX817320.1_Jerry_Slough_virus_strain_BFS_4474_segment_S_complete_sequence       CACC.CA.A. ..C..T..A. .T....GA.. A..C..A.G. ......A... .A........ C..A..A... ........C.  [400]
#KX817338.1_South_River_virus_strain_NJO-94F_segment_S_complete_sequence         .AC...A.A. .....T..A. .C..G.G... ...T..ACGA .....CA... .A........ ...A...... .....A....  [400]
#GU018050.2_South_River_virus_isolate_SORV-252_nucleoprotein_and_NSs_protein_gen .AC...A.A. .......... .C....GA.. ......ACG. .....CA.T. .A.....G.. ...A..A... ..........  [400]
#EF681804.1_Jamestown_Canyon_virus_isolate_5592-02_segment_S_nucleocapsid_protei .ACTACA.A. ..T..T.... .C..G.GA.. A..C..A.G. .....CA... .T........ ...A...... .....C....  [400]
#EF681805.1_Jamestown_Canyon_virus_isolate_368-99_segment_S_nucleocapsid_protein .ACTACA.A. ..T..T.... .C..G.GA.. A..C..A.G. .....CA... .T........ ...A...... .....C....  [400]
#EF681806.1_Jamestown_Canyon_virus_isolate_6163-03_segment_S_nucleocapsid_protei .ACTACA.A. ..T..T.... .C..G.GA.. A..C..A.G. .....CA... .T........ ...A...... .....C....  [400]
#EF681807.1_Jamestown_Canyon_virus_isolate_468-04_segment_S_nucleocapsid_protein CACC..A.A. ..T..T.... .T....GA.. .T.A..A.G. .....CA... .A........ ...A..A... .....C....  [400]
#EF681808.1_Jamestown_Canyon_virus_isolate_2179-00_segment_S_nucleocapsid_protei .ACTACA.A. ..T..T.... .C..G.GA.. A..C..A.G. .....CA... .T........ ...A...... .....C....  [400]
#EF681809.1_Jamestown_Canyon_virus_isolate_779-98_segment_S_nucleocapsid_protein CACC..A.A. ..T..T.... .T....GA.. .T.A..A.G. .....CA... .A........ ...A..A... .....C....  [400]
#EF681810.1_Jamestown_Canyon_virus_isolate_810-98_segment_S_nucleocapsid_protein CACC..A.A. ..T..T.... .T....GA.. .T.A..A.G. .....CA... .A........ ...A..A... .....C....  [400]
#EF681811.1_Jamestown_Canyon_virus_isolate_811-00_segment_S_nucleocapsid_protein .ACTACA.A. ..T..T.... .C..G.GA.. A..C..A.G. .....CA... .T........ ...A...... .....C....  [400]
#EF681812.1_Jamestown_Canyon_virus_isolate_1697-03_segment_S_nucleocapsid_protei CACC..A.A. ..T..T.... .T....GA.. .T.A..A.G. .....CA... .A........ ...A..A... .....C....  [400]
#EF681813.1_Jamestown_Canyon_virus_isolate_1425-02_segment_S_nucleocapsid_protei CACC..A.A. ..T..T.... .T....GA.. .T.A..A.G. .....CA... .A........ ...A..A... .....C....  [400]
#EF681814.1_Jamestown_Canyon_virus_isolate_1441-04_segment_S_nucleocapsid_protei CACC..A.A. ..T..T.... .T....GA.. .T.A..A.G. .....CA... .A........ ...A..A... .....C....  [400]
#EF681815.1_Jamestown_Canyon_virus_isolate_928-00_segment_S_nucleocapsid_protein CACC..A.A. ..T..T.... .T....GA.. .T.A..A.G. .....CA... .A........ ...A..A... .....C....  [400]
#EF681816.1_Jamestown_Canyon_virus_isolate_1064-03_segment_S_nucleocapsid_protei CACC..A.A. ..T....... .T..G.GA.. ...C..A.G. .....CA... .A........ ...A..A... .....C....  [400]
#EF681817.1_Jamestown_Canyon_virus_isolate_1369-02_segment_S_nucleocapsid_protei .ACTACA.A. ..T..T.... .C..G.GA.. A..C..A.G. .....CA... .T........ ...A...... .....C....  [400]
#EF681818.1_Jamestown_Canyon_virus_isolate_1627-04_segment_S_nucleocapsid_protei .ACTACA.A. ..T..T.... .C..G.GA.. A..C..A.G. .....CA... .T........ ...A...... .....C....  [400]
#EF681819.1_Jamestown_Canyon_virus_isolate_1810-02_segment_S_nucleocapsid_protei .ACTACA.A. ..T..T.... .C..G.GA.. A..C..A.G. .....CA... .T........ ...A...... .....C....  [400]
#EF681820.1_Jamestown_Canyon_virus_isolate_2384-98_segment_S_nucleocapsid_protei .ACTACA.A. ..T..T.... .C..G.GA.. A..C..A.G. .....CA... .T........ ...A...... .....C....  [400]
#EF681821.1_Jamestown_Canyon_virus_isolate_2707-01_segment_S_nucleocapsid_protei CACC..A.A. ..T..T.... .T....GA.. .T.A..A.G. .....CA... .A........ ...A..A... .....C....  [400]
#EF681822.1_Jamestown_Canyon_virus_isolate_2718-01_segment_S_nucleocapsid_protei .ACTACA.A. ..T..T.... .C..G.GA.. A..C..A.G. .....CA... .T........ ...A...... .....C....  [400]
#EF681823.1_Jamestown_Canyon_virus_isolate_3280-03_segment_S_nucleocapsid_protei CACC..A.A. ..T..T.... .T....GA.. .T.A..A.G. .....CA... .A........ ...A..A... .....C....  [400]
#EF681824.1_Jamestown_Canyon_virus_isolate_3324-04_segment_S_nucleocapsid_protei .ACTACA.A. ..T..T.... .C..G.GA.. A..C..A.G. .....CA... .T........ ...A...... .....C....  [400]
#EF681825.1_Jamestown_Canyon_virus_isolate_3573-03_segment_S_nucleocapsid_protei CACC..A.A. ..T..T.... .T....GA.. .T.A..A.G. .....CA... .A........ ...A..A... .....C....  [400]
#EF681826.1_Jamestown_Canyon_virus_isolate_3682-00_segment_S_nucleocapsid_protei CACC..A.A. ..T..T.... .T....GA.. .T.A..A.G. .....CA... .A........ ...A..A... .....C....  [400]
#EF681827.1_Jamestown_Canyon_virus_isolate_4148-03_segment_S_nucleocapsid_protei CACC..A.A. ..T....... .T..G.GA.. ...C..A.G. .....CA... .A........ ...A..A... .....C....  [400]
#EF681828.1_Jamestown_Canyon_virus_isolate_4473-00_segment_S_nucleocapsid_protei CACC..A.A. ..T....... .T..G.GA.. ...C..A.G. .....CA... .A........ ...A..A... .....C....  [400]
#EF681829.1_Jamestown_Canyon_virus_isolate_4742-04_segment_S_nucleocapsid_protei .ACTACA.A. ..T..T.... .C..G.GA.. A..C..A.G. .....CA... .T........ ...A...... .....C....  [400]
#EF681830.1_Jamestown_Canyon_virus_isolate_2274-05_segment_S_nucleocapsid_protei .ACTACA.A. ..T..T.... .C..G.GA.. A..C..A.G. .....CA... .T........ ...A...... .....C....  [400]
#EF681831.1_Jamestown_Canyon_virus_isolate_1472-05_segment_S_nucleocapsid_protei .ACTACA.A. ..T..T.... .C..G.GA.. A..C..A.G. .....CA... .T........ ...A...... .....C....  [400]
#EF681832.1_Jamestown_Canyon_virus_isolate_4910-02_segment_S_nucleocapsid_protei CACC..A.A. ..T..T.... .T....GA.. .T.A..A.G. .....CA... .A........ ...A..A... .....C....  [400]
#EF681833.1_Jamestown_Canyon_virus_isolate_275-01_segment_S_nucleocapsid_protein .ACTACA.A. ..T..T.... .C..G.GA.. A..C..A.G. .....CA... .T........ ...A...... .....C....  [400]
#EF681834.1_Jamestown_Canyon_virus_isolate_339-05_segment_S_nucleocapsid_protein .ACTACA.A. ..T..T.... .C..G.GA.. A..C..A.G. .....CA... .T........ ...A...... .....C....  [400]
#EF681835.1_Jamestown_Canyon_virus_isolate_3836-05_segment_S_nucleocapsid_protei .ACTACA.A. ..T..T.... .C..G.GA.. A..C..A.G. .....CA... .T........ ...A...... .....C....  [400]
#EF681836.1_Jamestown_Canyon_virus_isolate_2286-00_segment_S_nucleocapsid_protei CACC..A.A. ..T..T.... .T....GA.. .T.A..A.G. .....CA... .A........ ...A..A... .....C....  [400]
#EF681837.1_Jamestown_Canyon_virus_isolate_1044-05_segment_S_nucleocapsid_protei .ACTACA.A. ..T..T.... .C..G.GA.. A..C..A.G. .....CA... .T........ ...A...... .....C....  [400]
#EF681838.1_Jamestown_Canyon_virus_isolate_978-99_segment_S_nucleocapsid_protein .ACTACA.A. ..T..T.... .C..G.GA.. A..C..A.G. .....CA... .T........ ...A...... .....C....  [400]
#EF681839.1_Jamestown_Canyon_virus_isolate_4832-01_segment_S_nucleocapsid_protei .ACTACA.A. ..T..T.... .C..G.GA.. A..C..A.G. .....CA... .T........ ...A...... .....C....  [400]
#EF681841.1_Jamestown_Canyon_virus_isolate_7101-03_segment_S_nucleocapsid_protei .ACTACA.A. ..T..T.... .C..G.GA.. A..C..A.G. .....CA... .T........ ...A...... .....C....  [400]
#EF681842.1_Jamestown_Canyon_virus_isolate_Simsbury_segment_S_nucleocapsid_prote .ACTACA.A. ..T..T.... .C..G.GA.. A..C..A.G. .....CA... .T........ ...A...... .....C....  [400]
#EF681843.1_Jamestown_Canyon_virus_isolate_8011-03_segment_S_nucleocapsid_protei .ACTACA.A. ..T..T.... .C..G.GA.. A..C..A.G. .....CA... .T........ ...A...... .....C....  [400]
#EF681844.1_Jamestown_Canyon_virus_isolate_8536-03_segment_S_nucleocapsid_protei CACC..A.A. ..T..T.... .T....GA.. .T.A..A.G. .....CA... .A........ ...A..A... .....C....  [400]
#EF681845.1_Jamestown_Canyon_virus_isolate_11497-03_segment_S_nucleocapsid_prote .ACTACA.A. ..T..T.... .C..G.GA.. A..C..A.G. .....CA... .T........ ...A...... .....C....  [400]
#EF681846.1_Jamestown_Canyon_virus_isolate_13995-03_segment_S_nucleocapsid_prote .ACTACA.A. ..T..T.... .C..G.GA.. A..C..A.G. .....CA... .T........ ...A...... .....C....  [400]
#EF681847.1_Jamestown_Canyon_virus_isolate_1768-98_segment_S_nucleocapsid_protei CACC..A.A. ..T..T.... .T....GA.. .T.A..A.G. .....CA... .A........ ...A..A... .....C....  [400]
#EF681848.1_Jamestown_Canyon_virus_isolate_1385-06_segment_S_nucleocapsid_protei .ACTACA.A. ..T..T.... .C..G.GA.. A..C..A.G. .....CA... .T........ ...A...... .....C....  [400]
#EF681849.1_Jamestown_Canyon_virus_isolate_2989-06_segment_S_nucleocapsid_protei .ACTACA.A. ..T..T.... .C..G.GA.. A..C..A.G. .....CA... .T........ ...A...... .....C....  [400]
```

```
#EF681850.1_Jamestown_Canyon_virus_isolate_3381-06_segment_S_nucleocapsid_protei .ACTACA.A. ..T..T.... .C..G.GA.. A..C..A.G. .....CA... .T........ ...A...... .....C....  [400]
#EF681851.1_Jamestown_Canyon_virus_isolate_4095-06_segment_S_nucleocapsid_protei .ACTACA.A. ..T..T.... .C..G.GA.. A..C..A.G. .....CA... .T........ ...A...... .....C....  [400]
#EF681852.1_Jamestown_Canyon_virus_isolate_4078-06_segment_S_nucleocapsid_protei .ACTACA.A. ..T..T.... .C..G.GA.. A..C..A.G. .....CA... .T........ ...A...... .....C....  [400]
#EF681853.1_Jamestown_Canyon_virus_isolate_11-92_segment_S_nucleocapsid_protein_ CACC..A.A. ..T..T.... .T....GA.. .T.A..A.G. .....CA... .A........ ...A..A... .....C....  [400]
#EF681854.1_Jamestown_Canyon_virus_isolate_23-97_segment_S_nucleocapsid_protein_ CACC..A.A. ..T..T.... .T....GA.. .T.A..A.G. .....CA... .A........ ...A..A... .....C....  [400]
#EF681855.1_Jamestown_Canyon_virus_isolate_25-97_segment_S_nucleocapsid_protein_ .ACTACA.A. ..T..T.... .C..G.GA.. A..C..A.G. .....CA... .T........ ...A...... .....C....  [400]
#EF681856.1_Jamestown_Canyon_virus_isolate_29-97_segment_S_nucleocapsid_protein_ CACC..A.A. ..T..T.... .T....GA.. .T.A..A.G. .....CA... .A........ ...A..A... .....C....  [400]
#EF681857.1_Jamestown_Canyon_virus_isolate_423-99_segment_S_nucleocapsid_protein .ACTACA.A. ..T..T.... .C..G.GA.. A..C..A.G. .....CA... .T........ ...A...... .....C....  [400]
#EF681858.1_Jamestown_Canyon_virus_isolate_1262-98_segment_S_nucleocapsid_protei CACC..A.A. ..T..T.... .T....GA.. .T.A..A.G. .....CA... .A........ ...A..A... .....C....  [400]
#EF681859.1_Jamestown_Canyon_virus_isolate_3438-06_segment_S_nucleocapsid_protei .ACTACA.A. ..T..T.... .C..G.GA.. A..C..A.G. .....CA... .T........ ...A...... .....C....  [400]
#HM007350.1_Jamestown_Canyon_virus_strain_61V2235_nucleoprotein_and_NSs_protein_ .AACTCA.A. ..T....... .T..G.GA.. A..C..A.G. .....CA... .A........ ...A..A... .....A....  [400]
#HM007353.1_Jamestown_Canyon_virus_strain_3573-03_nucleoprotein_gene_complete_cd CACC..A.A. ..T..T.... .T....GA.. .T.A..A.G. .....CA... .A........ ...A..A... .....C....  [400]
#HM007356.1_Jamestown_Canyon_virus_strain_3324-04_nucleoprotein_and_NSs_protein_ .ACTACA.A. ..T..T.... .C..G.GA.. A..C..A.G. .....CA... .T........ ...A...... .....C....  [400]
#KM215518.1_Jamestown_Canyon_virus_isolate_F1819_nucleocapsid_protein_and_nonstr CACCACA.A. ..T..T.... .C....GA.. A..C..A.G. .....CA... .A........ ...A...... .....C....  [400]
#KM215519.1_Jamestown_Canyon_virus_isolate_F6626_nucleocapsid_protein_and_nonstr CACCACA.A. ..T..T.... .C....GA.. A..C..A.G. .....CA... .A........ ...A...... .....C....  [400]
#KM215520.1_Jamestown_Canyon_virus_isolate_ND0283_nucleocapsid_protein_and_nonst CACCACA.A. ..T..T.... .T....GA.. A..C..A.G. .....CA... .A........ ...A...... .....C....  [400]
#KM215521.1_Jamestown_Canyon_virus_isolate_ND6194_nucleocapsid_protein_and_nonst CACCACA.A. ..T..T.... .C....GA.. A..C..A.G. .....CA... .A........ ...A...... .....C....  [400]
#KM215522.1_Jamestown_Canyon_virus_isolate_W6701_nucleocapsid_protein_and_nonstr CACCACA.A. ..T..T.... .T....GA.. A..C..A.G. .....CA... .A........ ...A...... .....C....  [400]
#KM215523.1_Jamestown_Canyon_virus_isolate_W14530_nucleocapsid_protein_and_nonst CACCACA.A. ..T..T.... .C....GA.. A..C..A.G. .....CA... .A........ ...A...... .....C....  [400]
#KM215524.1_Jamestown_Canyon_virus_isolate_W16690_nucleocapsid_protein_and_nonst CACCACA.A. ..T..T.... .C....GA.. A..C..A.G. .....CA... .A........ ...A...... .....C....  [400]
#KM215525.1_Jamestown_Canyon_virus_isolate_W18699_nucleocapsid_protein_and_nonst CACC.CA.A. ..C..T..A. .T....GA.. A..C..A.GA .....CA... .A........ C..A..A... ........C.  [400]
#KM215526.1_Jamestown_Canyon_virus_isolate_W15316_nucleocapsid_protein_and_nonst CACCACA.A. ..T..T.... .T....GA.. A..C..A.G. .....CA... .A........ ...A...... .....C....  [400]
#KM215527.1_Jamestown_Canyon_virus_isolate_W17680_nucleocapsid_protein_and_nonst CACCACA.A. ..T..T.... .C....GA.. A..C..A.G. .....CA... .A........ ...A...... .....C....  [400]
#KM215528.1_Jamestown_Canyon_virus_isolate_W19543_nucleocapsid_protein_and_nonst CACCACA.A. ..T..T.... .T....GA.. A..C..A.G. .....CA... .A........ ...A...... .....C....  [400]
#KM215529.1_Jamestown_Canyon_virus_isolate_W19925_nucleocapsid_protein_and_nonst CACCACA.A. ..T..T.... .C....GA.. A..C..A.G. .....CA... .A........ ...A...... .....C....  [400]
#KM215530.1_Jamestown_Canyon_virus_isolate_W20764_nucleocapsid_protein_and_nonst CACCACA.A. ..T..T.... .T....GA.. A..C..A.G. .....CA... .A........ ...A...... .....C....  [400]
#KM215531.1_Jamestown_Canyon_virus_isolate_W22352_nucleocapsid_protein_and_nonst CACTACA.A. ..T..T.... .C..G.GA.. A..C..A.G. .....CA... .A........ ...A...... .....C....  [400]
#KM215532.1_Jamestown_Canyon_virus_isolate_F1829_nucleocapsid_protein_and_nonstr CACCACA.A. ..T..T.... .C....GA.. A..C..A.G. .....CA... .A........ ...A...... .....C....  [400]
#KM215533.1_Jamestown_Canyon_virus_isolate_F6228_nucleocapsid_protein_and_nonstr CACCACA.A. ..T..T.... .C....GA.. A..C..A.G. .....CA... .A........ ...A...... .....C....  [400]
#KM215534.1_Jamestown_Canyon_virus_isolate_F6235_nucleocapsid_protein_and_nonstr CACCACA.A. ..T..T.... .C....GA.. A..C..A.G. .....CA... .A........ ...A...... .....C....  [400]
#KM215535.1_Jamestown_Canyon_virus_isolate_F10095_nucleocapsid_protein_and_nonst CACCACA.A. ..T..T.... .C....GA.. A..C..A.G. .....CA... .A........ ...G...... .....C....  [400]
#KM215536.1_Jamestown_Canyon_virus_isolate_F13418_nucleocapsid_protein_and_nonst CACCACA.A. ..T..T.... .T....GA.. A..C..A.G. .....CA... .A........ ...A...... .....C....  [400]
#KM215537.1_Jamestown_Canyon_virus_isolate_F14162_nucleocapsid_protein_and_nonst CACCACA.A. ..T..T.... .C....GA.. A..C..A.G. .....CA... .A........ ...A...... .....C....  [400]
#KM215538.1_Jamestown_Canyon_virus_isolate_F14183_nucleocapsid_protein_and_nonst CACCACA.A. ..T..T.... .C....GA.. A..C..A.G. .....CA... .A........ ...A...... .....C....  [400]
#KM215539.1_Jamestown_Canyon_virus_isolate_F14278_nucleocapsid_protein_and_nonst CACCACA.A. ..T..T.... .T....GA.. A..C..A.G. .....CA... .A........ ...A...... .....A....  [400]
#KM215540.1_Jamestown_Canyon_virus_isolate_F16109_nucleocapsid_protein_and_nonst CACCACA.A. ..T..T.... .C....GA.. A..C..A.G. .....CA... .A........ ...A...... .....C....  [400]
#KM215541.1_Jamestown_Canyon_virus_isolate_W8270_nucleocapsid_protein_and_nonstr CACCACA.A. ..T..T.... .C....GA.. A..C..A.G. .....CA... .A........ ...A...... .....C....  [400]
#KM215542.1_Jamestown_Canyon_virus_isolate_W16455_nucleocapsid_protein_and_nonst CACCACA.A. ..T..T.... .C....GA.. A..C..A.G. .....CA... .A........ ...A...... .....C....  [400]
#KM215543.1_Jamestown_Canyon_virus_isolate_W18738_nucleocapsid_protein_and_nonst CACCACA.A. ..T..T.... .T....GA.. A..C..A.G. .....CA... .A........ ...A...... .....C....  [400]
#KM215544.1_Jamestown_Canyon_virus_isolate_F4276_nucleocapsid_protein_and_nonstr CACCACA.A. ..T..T.... .C....GA.. A..C..A.G. .....CA... .A........ ...A...... .....C....  [400]
#KM215545.1_Jamestown_Canyon_virus_isolate_W19962_nucleocapsid_protein_and_nonst CACCACA.A. ..T..T.... .C....GA.. A..C..A.G. .....CA... .A........ ...A...... .....C....  [400]
#KM215546.1_Jamestown_Canyon_virus_isolate_F5609_nucleocapsid_protein_and_nonstr CACCACA.A. ..T..T.... .C....GA.. A..C..A.G. .....CA... .A........ ...A...... .....T....  [400]
#KM215547.1_Jamestown_Canyon_virus_isolate_F7826_nucleocapsid_protein_and_nonstr CACCACA.A. ..T..T.... .C....GA.. A..C..A.G. .....CA... .A........ ...A...... .....C....  [400]
#KM215548.1_Jamestown_Canyon_virus_isolate_F7932_nucleocapsid_protein_and_nonstr .ACCACA.A. ..T..T.... .C....GA.. A..C..A.G. .....CA... .A........ ...A...... .....C....  [400]
#KM215549.1_Jamestown_Canyon_virus_isolate_F8532_nucleocapsid_protein_and_nonstr CACCACA.A. ..T..T.... .C....GA.. A..C..A.G. .....CA... .A........ ...A...... .....C....  [400]
#KM215550.1_Jamestown_Canyon_virus_isolate_F10720_nucleocapsid_protein_and_nonst CACCACA.A. ..T..T.... .C....GA.. A..C..A.G. .....CA... .A........ ...A...... .....C....  [400]
#KM215551.1_Jamestown_Canyon_virus_isolate_ND1300_nucleocapsid_protein_and_nonst .ACCACA.A. ..T..T.... .C....GA.. A..C..A.G. .....CA... .A........ ...A...... .....C....  [400]
#KM215552.1_Jamestown_Canyon_virus_isolate_W13135_nucleocapsid_protein_and_nonst CACCACA.A. ..T..T.... .C....GA.. A..C..A.G. .....CA... .A........ ...A...... .....C....  [400]
#KM215553.1_Jamestown_Canyon_virus_isolate_W16456_nucleocapsid_protein_and_nonst CACCACA.A. ..T..T.... .C....GA.. A..C..A.G. .....CA... .A........ ...A...... .....C....  [400]
#KM215554.1_Jamestown_Canyon_virus_isolate_W16590_nucleocapsid_protein_and_nonst CACCACA.A. ..T..T.... .C....GA.. A..C..A.G. .....CA... .A........ ...A...... .....C....  [400]
#KM215555.1_Jamestown_Canyon_virus_isolate_W19651_nucleocapsid_protein_and_nonst .ACCACA.A. ..T..T.... .C....GA.. A..C..A.G. .....CA... .A........ ...A...... .....C....  [400]
#KM215556.1_Jamestown_Canyon_virus_isolate_F3406_nucleocapsid_protein_and_nonstr .ACCACA.A. ..T..T.... .C....GA.. A..C..A.G. .....CA... .A........ ...A...... .....C....  [400]
#KM215557.1_Jamestown_Canyon_virus_isolate_F6577_nucleocapsid_protein_and_nonstr CACCACA.A. ..T..T.... .T....GA.. A..C..A.G. .....CA... .A........ ...A...... .....C....  [400]
#KM215558.1_Jamestown_Canyon_virus_isolate_ND374_nucleocapsid_protein_and_nonstr .ACCACA.A. ..T..T.... .C....GA.. A..C..A.G. .....CA... .A........ ...A...... .....C....  [400]
#KM215559.1_Jamestown_Canyon_virus_isolate_W18393_nucleocapsid_protein_and_nonst CACC.CA.A. ..C..T..A. .T....GA.. A..C..A.GA .....CA... .A........ C..A..A... ........C.  [400]
#KM215560.1_Jamestown_Canyon_virus_isolate_W18901_nucleocapsid_protein_and_nonst CACC.CA.A. ..C..T..A. .T....GA.. A..C..A.GA .....CA... .A........ C..A..A... ........C.  [400]
#KX817317.1_Jamestown_Canyon_virus_strain_61V2235_segment_S_complete_sequence    .AACTCA.A. ..T....... .T..G.GA.. A..C..A.G. .....CA... .A........ ...A..A... .....A....  [400]
```

```
#MH370817.1_Jamestown_Canyon_virus_isolate_L36708_segment_S_complete_sequence    CACC..A.A. ..T..T.... .T....RA.. .T.A..A.G. .....CA... .A........ ...A..A... .....C....  [400]
#MH370820.1_Jamestown_Canyon_virus_isolate_MN256-260_segment_S_complete_sequence CACCACA.A. ..T..T.... .C....GA.. A..C..A.G. .....CA... .A........ ...A...... .....C....  [400]
#U12799.1_Jamestown_Canyon_virus_DAV28_S_RNA_segment_N_and_NSs_protein_genes_com .AACTCA.A. ..T....... .T..G.GA.. A..C..A.G. .....CA... .A........ ...A..A... .....A....  [400]
#U12796.1_Jamestown_Canyon_virus_61v2235_S_RNA_segment_N_and_NSs_protein_genes_c .AACTCA.A. ..T....... .T..G.GA.. A..C..A.G. .....CA... .A........ ...A..A... .....A....  [400]
#KM215561.1_Jamestown_Canyon_virus_isolate_W23697_nucleocapsid_protein_and_nonst CACCACA.A. ..T..T.... .C....GA.. A..C..A.G. .....CA... .A........ ...A...... .....C....  [400]
#KT288271.1_Inkoo_virus_strain_LEIV-15248Iv_segment_S_nucleoprotein_(N)_gene_com CACT..A.A. ..C..T.... .C....GA.. A..C..A.G. .....CA... .A..C..G.. ...A..A..G .....C....  [400]
#KT288274.1_Inkoo_virus_strain_LEIV-18154Yak_segment_S_nucleoprotein_(N)_gene_co CACT....A. ..T..T.... .C....GA.. A..C..A.G. .....CA... .A..C..... ...A..A..A ..C..C..C.  [400]
#KT288275.1_Inkoo_virus_strain_LEIV-9874Kar_segment_S_nucleoprotein_(N)_gene_com CACT..A.A. ..C..T.... .C....GA.. A..C..A.G. .....CA... .A..C..G.. ...A..A..G .....C....  [400]
#KT288277.1_Inkoo_virus_strain_LEIV-18784Yak_segment_S_nucleoprotein_(N)_gene_co CACT....A. ..T..T.... .C....GA.. A..C..A.G. .....CA... .A..C..... ...A..A..A ..C..C..C.  [400]
#KT288280.1_Inkoo_virus_strain_LEIV-22780Tyum_segment_S_nucleoprotein_(N)_gene_c CACT..A.A. ..T..T.... .C....GA.. A..C..A.G. .....CA... .A..C..... ...A..A..A ..C..C..C.  [400]
#KT288283.1_Inkoo_virus_strain_LEIV-18152Yak_segment_S_nucleoprotein_(N)_gene_co CACT....A. ..T..T.... .C....GA.. A..C..A.G. .....CA... .A..C..... ...A..A..A ..C..C..C.  [400]
#KT288286.1_Inkoo_virus_strain_LEIV-21643Kra_segment_S_nucleoprotein_(N)_gene_co CACT..A.A. ..T..T.... .C....GA.. A..C..A.G. .....CA... .A..C..G.. ...A..A..A .....C....  [400]
#KX554935.1_Inkoo_virus_strain_Lovanger_nucleocapsid_protein_and_nonstructural_p CACT..A.A. ..C..T.... .C....GA.. A..C..A.G. .....CA... .A..C..G.. ...A..A..G ..C..C....  [400]
#U47137.1_Inkoo_virus_Prototype_KN3641_nucleocapsid_protein_and_non-structural_p CACT..A.A. ..C..T.... .C....GA.. A..C..A.GA .....CA... .A..C..G.. ...A..A..A .....C....  [400]
#U47138.1_Inkoo_virus_SW_AR_83-161_nucleocapsid_protein_and_non-structural_prote CACT..A.A. ..C..T.... .C....GA.. A..C..A.G. .....CA... .A..C..... ...A..A..G .....C....  [400]
#Z68496.1_Inkoo_virus_RNA_for_N_protein_and_RNA_for_NS_protein_strain_KN_3641    CACT..A.A. ..C..T.... .C....GA.. A..C..A.GA .....CA... .A..C..G.. ...A..A..A .....C....  [400]
```

```
#KT630290.1_Keystone_virus_strain_KEYV/Ochlerotatus_atlanticus/USA/KEYVLK01/2005 GCTGGGGCAA CGGTGTAGAA ATCTATCTCT CCTTCTTCCC AGGAACTGAA ATGTTTCTGG AATTGTTCAA ATTCTACCCA  [480]
#KT630293.1_Keystone_virus_strain_KEYV/Ochlerotatus_atlanticus/USA/KEYVLK02/2005 .......... T......... .......... .......... .......... .......... .......... ..........  [480]
#MH016786.1_Keystone_virus_strain_KEYV/Homo_sapiens/Gainesville-1/2016_nucleopro .......... T......... .......... .......... .......... .......... .......... ..........  [480]
#KX817323.1_Keystone_virus_strain_B64-5587.05_segment_S_complete_sequence        .......... .......... .......... .......... .......... .......... .......... ..........  [480]
#MG821231.1_Keystone_virus_isolate_AR14033_segment_S_complete_sequence           .......... T..G.C.... .......... .......T.. G......... ......T... .......... ..........  [480]
#MG765471.1_Keystone_virus_isolate_AVA1709441_nucleocapsid_and_NSs_genes_complet .......... T..G.C.... .......... .......T.. G......... .....CT... .......... ..........  [480]
#KX817329.1_Melao_virus_strain_TRVL_9375_segment_S_complete_sequence             A.....CA.. T..G..T... ..A..C..T. .A..T..... ...G...... .......... ..GCC....G ...T..T..T  [480]
#KX817335.1_Serra_do_Navio_virus_strain_BeAr_103645_segment_S_complete_sequence  AT....CA.. T..A.C.... ..T..C..T. .A........ .........G ........T. .GGCT..... ..........  [480]
#KX817320.1_Jerry_Slough_virus_strain_BFS_4474_segment_S_complete_sequence       AT...AA... ....CC.... ..T.....T. .A..T..... ......C... .....C..A. ..A.C..... G........G  [480]
#KX817338.1_South_River_virus_strain_NJO-94F_segment_S_complete_sequence         AT...AA... T...CC.... ..T..C..T. .A.....T.. ......A... .......... ..GCT..... .........T  [480]
#GU018050.2_South_River_virus_isolate_SORV-252_nucleoprotein_and_NSs_protein_gen .T...AA... T...CC.... ........T. .A..T..... T.....A... ......T... .GGCT..... .........T  [480]
#EF681804.1_Jamestown_Canyon_virus_isolate_5592-02_segment_S_nucleocapsid_protei AT...AA... T..CCC...G ..T.....T. .A.....T.. ......A... ........T. ..A.T..... ......T..C  [480]
#EF681805.1_Jamestown_Canyon_virus_isolate_368-99_segment_S_nucleocapsid_protein AT...AA... T..CCC...G ..T.....T. .A.....T.. ......A... .......... ..A.T..... ......T..C  [480]
#EF681806.1_Jamestown_Canyon_virus_isolate_6163-03_segment_S_nucleocapsid_protei AT...AA... T..CCC...G ..T.....T. .A.....T.. ......A... .......... ..A.T..... ......T..C  [480]
#EF681807.1_Jamestown_Canyon_virus_isolate_468-04_segment_S_nucleocapsid_protein AT...AA... T..ACC.... ..T..C..T. .A..T..... ...G..A... .....CT... ..G.T..... ...T.....C  [480]
#EF681808.1_Jamestown_Canyon_virus_isolate_2179-00_segment_S_nucleocapsid_protei AT...AA... T..CCC...G ..T.....T. .A.....T.. ......A... .......... ..A.T..... ......T..C  [480]
#EF681809.1_Jamestown_Canyon_virus_isolate_779-98_segment_S_nucleocapsid_protein AT...AA... T..ACC.... ..T..C..T. .A..T..... ...G..A... .....CT... ..G.T..... ...T.....C  [480]
#EF681810.1_Jamestown_Canyon_virus_isolate_810-98_segment_S_nucleocapsid_protein AT...AA... T..ACC.... ..T..C..T. .A..T..... ...G..A... .....CT... ..G.T..... ...T.....C  [480]
#EF681811.1_Jamestown_Canyon_virus_isolate_811-00_segment_S_nucleocapsid_protein AT...AA... T..CCC...G ..T.....T. .A.....T.. ......A... .......... ..A.T..... ......T..C  [480]
#EF681812.1_Jamestown_Canyon_virus_isolate_1697-03_segment_S_nucleocapsid_protei AT...AA... T..ACC.... ..T..C..T. .A..T..... ...G..A... .....CT... ..G.T..... ...T.....C  [480]
#EF681813.1_Jamestown_Canyon_virus_isolate_1425-02_segment_S_nucleocapsid_protei AT...AA... T..ACC.... ..T..C..T. .A..T..... ...G..A... .....CT... ..G.T..... ...T.....C  [480]
#EF681814.1_Jamestown_Canyon_virus_isolate_1441-04_segment_S_nucleocapsid_protei AT...AA... T..ACC.... ..T..C..T. .G..T..... ...G..A... .....CT... ..G.T..... ...T.....C  [480]
#EF681815.1_Jamestown_Canyon_virus_isolate_928-00_segment_S_nucleocapsid_protein AT...AA... T..ACC.... ..T..C..T. .A..T..... ...G..A... .....CT... ..G.T..... ...T.....C  [480]
#EF681816.1_Jamestown_Canyon_virus_isolate_1064-03_segment_S_nucleocapsid_protei AT...AA... T..CCC.... ..T..C..T. .A........ ...G..A... .......... ..G.T..T.. .........C  [480]
#EF681817.1_Jamestown_Canyon_virus_isolate_1369-02_segment_S_nucleocapsid_protei AT...AA... T..CCC...G ..T.....T. .A.....T.. ......A... .......... ..A.T..... ......T..C  [480]
#EF681818.1_Jamestown_Canyon_virus_isolate_1627-04_segment_S_nucleocapsid_protei AT...AA... T..CCC...G ..T.....T. .A.....T.. ......A... .......... ..A.T..... ......T..C  [480]
#EF681819.1_Jamestown_Canyon_virus_isolate_1810-02_segment_S_nucleocapsid_protei AT...AA... T..CCC...G ..T.....T. .A.....T.. ......A... .......... ..A.T..... ......T..C  [480]
#EF681820.1_Jamestown_Canyon_virus_isolate_2384-98_segment_S_nucleocapsid_protei AT...AA... T..CCC...G ..T.....T. .A.....T.. ......A... .......... ..A.T..... ......T..C  [480]
#EF681821.1_Jamestown_Canyon_virus_isolate_2707-01_segment_S_nucleocapsid_protei AT...AA... T..ACC.... ..T..C..T. .A..T..... ...G..A... .....CT... ..G.T..... ...T.....C  [480]
#EF681822.1_Jamestown_Canyon_virus_isolate_2718-01_segment_S_nucleocapsid_protei AT...AA... T..CCC...G ..T.....T. .A.....T.. ......A... .......... ..A.T..... ......T..C  [480]
#EF681823.1_Jamestown_Canyon_virus_isolate_3280-03_segment_S_nucleocapsid_protei AT...AA... T..ACC.... ..T..C..T. .A..T..... ...G..A... .....CT... ..G.T..... ...T.....C  [480]
#EF681824.1_Jamestown_Canyon_virus_isolate_3324-04_segment_S_nucleocapsid_protei AT...AA... T..CCC...G ..T.....T. .A.....T.. ......A... .......... ..A.T..... ......T..C  [480]
#EF681825.1_Jamestown_Canyon_virus_isolate_3573-03_segment_S_nucleocapsid_protei AT...AA... T..ACC.... ..T..C..T. .A..T..... ...G..A... .....CT... ..G.T..... ...T.....C  [480]
#EF681826.1_Jamestown_Canyon_virus_isolate_3682-00_segment_S_nucleocapsid_protei AT...AA... T..ACC.... ..T..C..T. .A..T..... ...G..A... .....CT... ..G.T..... ...T.....C  [480]
#EF681827.1_Jamestown_Canyon_virus_isolate_4148-03_segment_S_nucleocapsid_protei AT...AA... T..CCC.... ..T..C..T. .A........ ...G..A... .......... ..G.T..T.. .........C  [480]
#EF681828.1_Jamestown_Canyon_virus_isolate_4473-00_segment_S_nucleocapsid_protei AT...AA... T..CCC.... ..T..C..T. .A........ ...G..A... .......... ..G.T..T.. .........C  [480]
#EF681829.1_Jamestown_Canyon_virus_isolate_4742-04_segment_S_nucleocapsid_protei AT...AA... T..CCC...G ..T.....T. .A.....T.. ......A... .......... ..A.T..... ......T..C  [480]
#EF681830.1_Jamestown_Canyon_virus_isolate_2274-05_segment_S_nucleocapsid_protei AT...AA... T..CCC...G ..T.....T. .A.....T.. ......A... .......... ..A.T..... ......T..C  [480]
#EF681831.1_Jamestown_Canyon_virus_isolate_1472-05_segment_S_nucleocapsid_protei AT...AA... T..CCC...G ..T.....T. .A.....T.. ......A... .......... ..A.T..... ......T..C  [480]
#EF681832.1_Jamestown_Canyon_virus_isolate_4910-02_segment_S_nucleocapsid_protei AT...AA... T..ACC.... ..T..C..T. .A..T..... ...G..A... .....CT... ..G.T..... .........C  [480]
#EF681833.1_Jamestown_Canyon_virus_isolate_275-01_segment_S_nucleocapsid_protein AT...AA... T..CCC...G ..T.....T. .A.....T.. ......A... .......... ..A.T..... ......T..C  [480]
#EF681834.1_Jamestown_Canyon_virus_isolate_339-05_segment_S_nucleocapsid_protein AT...AA... T..CCC...G ..T.....T. .A.....T.. ......A... .......... ..A.T..... ......T..C  [480]
#EF681835.1_Jamestown_Canyon_virus_isolate_3836-05_segment_S_nucleocapsid_protei AT...AA... T..CCC...G ..T.....T. .A.....T.. ......A... .......... ..A.T..... ......T..C  [480]
#EF681836.1_Jamestown_Canyon_virus_isolate_2286-00_segment_S_nucleocapsid_protei AT...AA... T..ACC.... ..T..C..T. .A..T..... ...G..A... .....CT... ..G.T..... ...T.....C  [480]
#EF681837.1_Jamestown_Canyon_virus_isolate_1044-05_segment_S_nucleocapsid_protei AT...AA... T..CCC...G ..T.....T. .A.....T.. ......A... .......... ..A.T..... ......T..C  [480]
#EF681838.1_Jamestown_Canyon_virus_isolate_978-99_segment_S_nucleocapsid_protein AT...AA... T..CCC...G ..T.....T. .A.....T.. ......A... .......... ..A.T..... ......T..C  [480]
#EF681839.1_Jamestown_Canyon_virus_isolate_4832-01_segment_S_nucleocapsid_protei AT...AA... T..CCC...G ..T.....T. .A.....T.. ......A... .......... ..A.T..... ......T..C  [480]
#EF681841.1_Jamestown_Canyon_virus_isolate_7101-03_segment_S_nucleocapsid_protei AT...AA... T..CCC...G ..T.....T. .A.....T.. ......A... .......... ..A.T..... ......T..C  [480]
#EF681842.1_Jamestown_Canyon_virus_isolate_Simsbury_segment_S_nucleocapsid_prote AT...AA... T..CCC...G ..T.....T. .A.....T.. ......A... ......T... ..A.T..... ......T..C  [480]
#EF681843.1_Jamestown_Canyon_virus_isolate_8011-03_segment_S_nucleocapsid_protei AT...AA... T..CCC...G ..T.....T. .A.....T.. ......A... .......... ..A.T..... ......T..C  [480]
#EF681844.1_Jamestown_Canyon_virus_isolate_8536-03_segment_S_nucleocapsid_protei AT...AA... T..ACC.... ..T..C..T. .A..T..... ...G..A... .....CT... ..G.T..... ...T.....C  [480]
#EF681845.1_Jamestown_Canyon_virus_isolate_11497-03_segment_S_nucleocapsid_prote AT...AA... T..CCC...G ..T.....T. .A.....T.. ......A... .......... ..A.T..... ......T..C  [480]
#EF681846.1_Jamestown_Canyon_virus_isolate_13995-03_segment_S_nucleocapsid_prote AT...AA... T..CCC...G ..T.....T. .A.....T.. ......A... .......... ..A.T..... ......T..C  [480]
#EF681847.1_Jamestown_Canyon_virus_isolate_1768-98_segment_S_nucleocapsid_protei AT...AA... T..ACC.... ..T..C..T. .A..T..... ...G..A... .....CT... ..G.T..... ...T.....C  [480]
#EF681848.1_Jamestown_Canyon_virus_isolate_1385-06_segment_S_nucleocapsid_protei AT...AA... T..CCC...G ..T.....T. .A.....T.. ......A... .......... ..A.T..... ......T..C  [480]
#EF681849.1_Jamestown_Canyon_virus_isolate_2989-06_segment_S_nucleocapsid_protei AT...AA... T..CCC...G ..T.....T. .A.....T.. ......A... .......... ..A.T..... ......T..C  [480]
```

```
#EF681850.1_Jamestown_Canyon_virus_isolate_3381-06_segment_S_nucleocapsid_protei AT...AA... T..CCC...G ..T.....T. .A.....T.. ......A... .......... ..A.T..... ......T..C  [480]
#EF681851.1_Jamestown_Canyon_virus_isolate_4095-06_segment_S_nucleocapsid_protei AT...AA... T..CCC...G ..T.....T. .A.....T.. ......A... .......... ..A.T..... ......T..C  [480]
#EF681852.1_Jamestown_Canyon_virus_isolate_4078-06_segment_S_nucleocapsid_protei AT...AA... T..CCC...G ..T.....T. .A.....T.. ......A... .......... ..A.T..... ......T..C  [480]
#EF681853.1_Jamestown_Canyon_virus_isolate_11-92_segment_S_nucleocapsid_protein_ AT...AA... T..ACC.... ..T..C..T. .A..T..... ...G..A... .....CT... ..G.T..... ...T.....C  [480]
#EF681854.1_Jamestown_Canyon_virus_isolate_23-97_segment_S_nucleocapsid_protein_ AT...AA... T..ACC.... ..T..C..T. .A..T..... ...G..A... .....CT... ..G.T..... ...T.....C  [480]
#EF681855.1_Jamestown_Canyon_virus_isolate_25-97_segment_S_nucleocapsid_protein_ AT...AA... T..CCC...G ..T.....T. .A.....T.. ......A... .......... ..A.T..... ......T..C  [480]
#EF681856.1_Jamestown_Canyon_virus_isolate_29-97_segment_S_nucleocapsid_protein_ AT...AA... T..ACC.... ..T..C..T. .A..T..... ...G..A... .....CT... ..G.T..... ...T.....C  [480]
#EF681857.1_Jamestown_Canyon_virus_isolate_423-99_segment_S_nucleocapsid_protein AT...AA... T..CCC...G ..T.....T. .A.....T.. ......A... .......... ..A.T..... ......T..C  [480]
#EF681858.1_Jamestown_Canyon_virus_isolate_1262-98_segment_S_nucleocapsid_protei AT...AA... T..ACC.... ..T..C..T. .A..T..... ...G..A... .....CT... ..G.T..... ...T.....C  [480]
#EF681859.1_Jamestown_Canyon_virus_isolate_3438-06_segment_S_nucleocapsid_protei AT...AA... T..CCC...G ..T.....T. .A.....T.. ......A... .......... ..A.T..... ......T..C  [480]
#HM007350.1_Jamestown_Canyon_virus_strain_61V2235_nucleoprotein_and_NSs_protein_ AT...AA... T..CCC.... ..T.....T. .G........ ...G..C... .....C..A. ..A.T..... G........G  [480]
#HM007353.1_Jamestown_Canyon_virus_strain_3573-03_nucleoprotein_gene_complete_cd AT...AA... T..ACC.... ..T..C..T. .A..T..... ...G..A... .....CT... ..G.T..... ...T.....C  [480]
#HM007356.1_Jamestown_Canyon_virus_strain_3324-04_nucleoprotein_and_NSs_protein_ AT...AA... T..CCC...G ..T.....T. .A.....T.. ......A... .......... ..A.T..... ......T..C  [480]
#KM215518.1_Jamestown_Canyon_virus_isolate_F1819_nucleocapsid_protein_and_nonstr AT...AA... T..CCC...G ..T.....T. .A........ ...G..A... .......... ..A.T..... ......T..C  [480]
#KM215519.1_Jamestown_Canyon_virus_isolate_F6626_nucleocapsid_protein_and_nonstr AT...AA... T..CCC...G ..T.....T. .A........ ...G..A... .......... ..A.T..... ......T..C  [480]
#KM215520.1_Jamestown_Canyon_virus_isolate_ND0283_nucleocapsid_protein_and_nonst AT...AA... T..CCC...G ..T.....T. .A........ ......A... .......... ..A.T..... ......T..C  [480]
#KM215521.1_Jamestown_Canyon_virus_isolate_ND6194_nucleocapsid_protein_and_nonst AT...AA... T..CCC...G ..T.....T. .A........ ......A... .......... ..A.T..... ......T..C  [480]
#KM215522.1_Jamestown_Canyon_virus_isolate_W6701_nucleocapsid_protein_and_nonstr AT...AA... T..CCC...G ..T.....T. .A........ ......A... .......... ..A.T..... ......T..C  [480]
#KM215523.1_Jamestown_Canyon_virus_isolate_W14530_nucleocapsid_protein_and_nonst AT...AA... T..CCC...G ..T..C..T. .A........ ......A... .......... ..A.T..... .........C  [480]
#KM215524.1_Jamestown_Canyon_virus_isolate_W16690_nucleocapsid_protein_and_nonst AT...AA... T..CCC...G ..T..C..T. .A........ ......A... .......... ..A.T..... .........C  [480]
#KM215525.1_Jamestown_Canyon_virus_isolate_W18699_nucleocapsid_protein_and_nonst AT...AA... T...CC.... ..T.....T. .A..T..... ......C... .....C..A. ..A.C..... G........G  [480]
#KM215526.1_Jamestown_Canyon_virus_isolate_W15316_nucleocapsid_protein_and_nonst AT...AA... T..CCC...G ..T.....T. .A........ ...G..A... .......... ..A.T..... ......T..C  [480]
#KM215527.1_Jamestown_Canyon_virus_isolate_W17680_nucleocapsid_protein_and_nonst AT...AA... T..CCC...G ..T.....T. .A........ ......A... .......... ..A.T..... ......T..C  [480]
#KM215528.1_Jamestown_Canyon_virus_isolate_W19543_nucleocapsid_protein_and_nonst AT...AA... T..CCC...G ..T.....T. .A........ ......A... .......... ..A.T..... ......T..C  [480]
#KM215529.1_Jamestown_Canyon_virus_isolate_W19925_nucleocapsid_protein_and_nonst AT...AA... T..CCC...G ..T..C..T. .A........ ......A... .......... ..A.T..... .........C  [480]
#KM215530.1_Jamestown_Canyon_virus_isolate_W20764_nucleocapsid_protein_and_nonst AT...AA... T..CCC...G ..T.....T. .A.....T.. ......A... .......... ..A.T..... ......T..C  [480]
#KM215531.1_Jamestown_Canyon_virus_isolate_W22352_nucleocapsid_protein_and_nonst AT...AA... T..CCC...G ..T.....T. .A.....T.. ......G... .......... ..A.T..... ......T..C  [480]
#KM215532.1_Jamestown_Canyon_virus_isolate_F1829_nucleocapsid_protein_and_nonstr AT...AA... T..CCC...G ..T.....T. .A........ ...G..A... .......... ..A.T..... ......T..C  [480]
#KM215533.1_Jamestown_Canyon_virus_isolate_F6228_nucleocapsid_protein_and_nonstr AT...AA... T..CCC...G ..T.....T. .A........ ...G..A... .......... ..A.T..... ......T..C  [480]
#KM215534.1_Jamestown_Canyon_virus_isolate_F6235_nucleocapsid_protein_and_nonstr AT...AA... T..CCC...G ..T.....T. .A........ ...G..A... .......... ..A.T..... ......T..C  [480]
#KM215535.1_Jamestown_Canyon_virus_isolate_F10095_nucleocapsid_protein_and_nonst AT...AA... T..CCC...G ..T.....T. .A........ ......A... .......... ..A.T..... ......T..C  [480]
#KM215536.1_Jamestown_Canyon_virus_isolate_F13418_nucleocapsid_protein_and_nonst AT...AA... T..CCC...G ..T.....T. .A........ ......A... .......... ..A.T..... ......T..C  [480]
#KM215537.1_Jamestown_Canyon_virus_isolate_F14162_nucleocapsid_protein_and_nonst AT...AA... T..CCC...G ..T..C..T. .A........ ......A... .......... ..A.T..... .........C  [480]
#KM215538.1_Jamestown_Canyon_virus_isolate_F14183_nucleocapsid_protein_and_nonst AT...AA... T..CCC...G ..T..C..T. .A........ ......A... .......... ..A.T..... .........C  [480]
#KM215539.1_Jamestown_Canyon_virus_isolate_F14278_nucleocapsid_protein_and_nonst AT...AA... T..CCC...G ..T.....T. .A........ ...G..A... .......... ..A.T..... ......T..C  [480]
#KM215540.1_Jamestown_Canyon_virus_isolate_F16109_nucleocapsid_protein_and_nonst AT...AA... T..CCC...G ..T.....T. .A........ ......A... .......... ..A.T..... ......T..C  [480]
#KM215541.1_Jamestown_Canyon_virus_isolate_W8270_nucleocapsid_protein_and_nonstr AT...AA... T..CCC...G ..T.....T. .A........ ...G..A... .......... ..A.T..... ......T..C  [480]
#KM215542.1_Jamestown_Canyon_virus_isolate_W16455_nucleocapsid_protein_and_nonst AT...AA... T..CCC...G ..T..C..T. .A........ ......A... .......... ..A.T..... .........C  [480]
#KM215543.1_Jamestown_Canyon_virus_isolate_W18738_nucleocapsid_protein_and_nonst AT...AA... T..CCC...G ..T.....T. .A........ ......A... .......... ..A.T..... ......T..C  [480]
#KM215544.1_Jamestown_Canyon_virus_isolate_F4276_nucleocapsid_protein_and_nonstr AT...AA... T..CCC...G ..T.....T. .A........ ......A... .......... ..A.T..... ......T..C  [480]
#KM215545.1_Jamestown_Canyon_virus_isolate_W19962_nucleocapsid_protein_and_nonst AT...AA... T..CCC...G ..T..C..T. .A........ ......A... .......... ..A.T..... .........C  [480]
#KM215546.1_Jamestown_Canyon_virus_isolate_F5609_nucleocapsid_protein_and_nonstr AT...AA... T..CCC...G ..T.....T. .A..T..... ...G..A... .......... ..A.T..... ......T..C  [480]
#KM215547.1_Jamestown_Canyon_virus_isolate_F7826_nucleocapsid_protein_and_nonstr AT...AA... T..CCC...G ..T.....T. .A........ ...G..A... .......... ..A.T..... ......T..C  [480]
#KM215548.1_Jamestown_Canyon_virus_isolate_F7932_nucleocapsid_protein_and_nonstr AT...AA... T..CCC...G ..T..C..T. .A........ ......A... .......... ..A.T..... ......T..C  [480]
#KM215549.1_Jamestown_Canyon_virus_isolate_F8532_nucleocapsid_protein_and_nonstr AT...AA... T..CCC...G ..T.....T. .A........ ...G..A... .......... ..A.T..... ......T..C  [480]
#KM215550.1_Jamestown_Canyon_virus_isolate_F10720_nucleocapsid_protein_and_nonst AT...AA... T..CCC...G ..T.....T. .A........ ...G..A... .......... ..A.T..... ......T..C  [480]
#KM215551.1_Jamestown_Canyon_virus_isolate_ND1300_nucleocapsid_protein_and_nonst AT...AA... T..CCC...G ..T.....T. .A........ ......A... .......... ..A.T..... ......T..C  [480]
#KM215552.1_Jamestown_Canyon_virus_isolate_W13135_nucleocapsid_protein_and_nonst AT...AA... T..CCC...G ..T.....T. .A........ ...G..A... .......... ..A.T..... ......T..C  [480]
#KM215553.1_Jamestown_Canyon_virus_isolate_W16456_nucleocapsid_protein_and_nonst AT...AA... T..CCC...G ..T..C..T. .A........ ......A... .......... ..A.T..... .........C  [480]
#KM215554.1_Jamestown_Canyon_virus_isolate_W16590_nucleocapsid_protein_and_nonst AT...AA... T..CCC...G ..T.....T. .A........ ...G..A... .......... ..A.T..... ......T..C  [480]
#KM215555.1_Jamestown_Canyon_virus_isolate_W19651_nucleocapsid_protein_and_nonst AT...AA... T..CCC...G ..T.....T. .A........ ......A... .......... ..A.T..... ......T..C  [480]
#KM215556.1_Jamestown_Canyon_virus_isolate_F3406_nucleocapsid_protein_and_nonstr AT...AA... T..CCC...G ..T.....T. .A........ ......A... .......... ..A.T..... ......T..C  [480]
#KM215557.1_Jamestown_Canyon_virus_isolate_F6577_nucleocapsid_protein_and_nonstr AT...AA... T..CCC...G ..T.....T. .A........ ......A... .......... ..A.T..... ......T..C  [480]
#KM215558.1_Jamestown_Canyon_virus_isolate_ND374_nucleocapsid_protein_and_nonstr AT...AA... T..CCC...G ..T.....T. .A........ ......A... .......... ..A.T..... ......T..C  [480]
#KM215559.1_Jamestown_Canyon_virus_isolate_W18393_nucleocapsid_protein_and_nonst AT...AA... T...CC.... ..T.....T. .A..T..... ......C... .....C..A. ..A.C..... G........G  [480]
#KM215560.1_Jamestown_Canyon_virus_isolate_W18901_nucleocapsid_protein_and_nonst AT...AA... T...CC.... ..T.....T. .A..T..... ......C... .....C..A. ..A.C..... G........G  [480]
#KX817317.1_Jamestown_Canyon_virus_strain_61V2235_segment_S_complete_sequence    AT...AA... T..CCC.... ..T.....T. .G........ ...G..C... .....C..A. ..A.T..... G........G  [480]
```

```
#MH370817.1_Jamestown_Canyon_virus_isolate_L36708_segment_S_complete_sequence    AT...AA... T..ACC.... ..T..C..T. .A..T..... ...G..A... .....CT... ..G.T..... ...T.....C  [480]
#MH370820.1_Jamestown_Canyon_virus_isolate_MN256-260_segment_S_complete_sequence AT...AA... T..CCC...G ..T.....T. .A........ ......A... .......... ..A.T..... ......T..C  [480]
#U12799.1_Jamestown_Canyon_virus_DAV28_S_RNA_segment_N_and_NSs_protein_genes_com AT...AA... T..CCC.... ..T.....T. .G........ ...G..C... .....C..A. ..A.T..... G........G  [480]
#U12796.1_Jamestown_Canyon_virus_61v2235_S_RNA_segment_N_and_NSs_protein_genes_c AT...AA... T..CCC.... ..T.....T. .G........ ...G..C... .....C..A. ..A.T..... G........G  [480]
#KM215561.1_Jamestown_Canyon_virus_isolate_W23697_nucleocapsid_protein_and_nonst AT...AA... T..CCC...G ..T..C..T. .A........ ......A... .......... ..A.T..... .........C  [480]
#KT288271.1_Inkoo_virus_strain_LEIV-15248Iv_segment_S_nucleoprotein_(N)_gene_com AT...AA... T..CCC.... ..T..C..T. .A........ ......A... ........A. ..G.T..... .........T  [480]
#KT288274.1_Inkoo_virus_strain_LEIV-18154Yak_segment_S_nucleoprotein_(N)_gene_co AT...AC... T..CCC.... ..T..C..T. .A........ ......A... .....C..A. ..G.T..... .........T  [480]
#KT288275.1_Inkoo_virus_strain_LEIV-9874Kar_segment_S_nucleoprotein_(N)_gene_com AT...AA... T..CCC.... ..T..C..T. .A........ ......A... ........A. ..G.T..... .........T  [480]
#KT288277.1_Inkoo_virus_strain_LEIV-18784Yak_segment_S_nucleoprotein_(N)_gene_co AT...AC... T..CCC.... ..T..C..T. .A........ ......A... .....C..A. ..G.T..... .........T  [480]
#KT288280.1_Inkoo_virus_strain_LEIV-22780Tyum_segment_S_nucleoprotein_(N)_gene_c AT...AC... T..CCC.... ..T..C..T. .A........ ......A... .....C..A. ..G.T..... .........T  [480]
#KT288283.1_Inkoo_virus_strain_LEIV-18152Yak_segment_S_nucleoprotein_(N)_gene_co AT...AC... T..CCC.... ..T..C..T. .A........ ......A... .....C..A. ..G.T..... .........T  [480]
#KT288286.1_Inkoo_virus_strain_LEIV-21643Kra_segment_S_nucleoprotein_(N)_gene_co AT...AA... T..CCC.... ..T..C..T. .A........ ......A... ........A. ..G.T..... .........T  [480]
#KX554935.1_Inkoo_virus_strain_Lovanger_nucleocapsid_protein_and_nonstructural_p AT...AA... T..CCC.... ..T..C..T. .A........ ......A... ........A. ..G.T..... .........T  [480]
#U47137.1_Inkoo_virus_Prototype_KN3641_nucleocapsid_protein_and_non-structural_p AT...AA... T..CCC.... ..T.....T. .A........ ......A... ........A. ..G.T..... .........T  [480]
#U47138.1_Inkoo_virus_SW_AR_83-161_nucleocapsid_protein_and_non-structural_prote AT...AA... T..CCC.... ..T..C..T. .A...C.... ......A... ........A. ..G.T..... .........T  [480]
#Z68496.1_Inkoo_virus_RNA_for_N_protein_and_RNA_for_NS_protein_strain_KN_3641    AT...AA... T..CCC.... ..T.....T. .A........ ......A... ........A. ..G.T..... .........T  [480]
```

```
#KT630290.1_Keystone_virus_strain_KEYV/Ochlerotatus_atlanticus/USA/KEYVLK01/2005 TTGACAATTG GCATCTACAG AGTGAAACAT GGAATGATGG ATGCTCAGTA CCTAAAAAAA GCTCTGAGAC AGCGCTATGG  [560]
#KT630293.1_Keystone_virus_strain_KEYV/Ochlerotatus_atlanticus/USA/KEYVLK02/2005 .......... .......... .......... .......... .......... .......... .......... .......C..  [560]
#MH016786.1_Keystone_virus_strain_KEYV/Homo_sapiens/Gainesville-1/2016_nucleopro .......... .......... .......... .......... .......... .......... .......... ..........  [560]
#KX817323.1_Keystone_virus_strain_B64-5587.05_segment_S_complete_sequence        .......... .......... .......... .......... .......... .......... .......... ..........  [560]
#MG821231.1_Keystone_virus_isolate_AR14033_segment_S_complete_sequence           .......... ....T..... ...A..G... .......... .......... ...C..G..G .......... ..........  [560]
#MG765471.1_Keystone_virus_isolate_AVA1709441_nucleocapsid_and_NSs_genes_complet .......... ....T..... ...A..G... .......... .......... ...C..G..G .......... ..........  [560]
#KX817329.1_Melao_virus_strain_TRVL_9375_segment_S_complete_sequence             C.A....... .A........ ...C.....C ..GC...... .CC....... T.....G..G .....AC... ..........  [560]
#KX817335.1_Serra_do_Navio_virus_strain_BeAr_103645_segment_S_complete_sequence  C......... .G..A..... ...C..G... .......... ....C..... ...G..G..G ..AT...... ....A.....  [560]
#KX817320.1_Jerry_Slough_virus_strain_BFS_4474_segment_S_complete_sequence       ..A..C.... .A..T..... ...C...... ..T....... ..C.C..... T..G..G..G .....C..G. .A........  [560]
#KX817338.1_South_River_virus_strain_NJO-94F_segment_S_complete_sequence         C.A..C.... .A..T..... ...C...... ..TC...... ..C.C..A.. .T....G... ..C..C.... .A.....C..  [560]
#GU018050.2_South_River_virus_isolate_SORV-252_nucleoprotein_and_NSs_protein_gen C.A..C.... .A..T..... ...C..G... ..TT...... ..C....... TT.G..G... ..C..C..G. .A........  [560]
#EF681804.1_Jamestown_Canyon_virus_isolate_5592-02_segment_S_nucleocapsid_protei .....C.... .A..T..... ...C..G... ..T....... .CC....... T..G..G..G .....C.... ..........  [560]
#EF681805.1_Jamestown_Canyon_virus_isolate_368-99_segment_S_nucleocapsid_protein .....C.... .A..T..... ...C..G... ..T....... .CC....... T..G..G..G .....C.... ..........  [560]
#EF681806.1_Jamestown_Canyon_virus_isolate_6163-03_segment_S_nucleocapsid_protei .....C.... .A..T..... ...C..G... ..T....... .CC....... T..G..G..G ..C..C.... ..........  [560]
#EF681807.1_Jamestown_Canyon_virus_isolate_468-04_segment_S_nucleocapsid_protein ..A..C..C. .G..T..... ...C...... ..T....... ..C....... .T.......G .....C.... .A........  [560]
#EF681808.1_Jamestown_Canyon_virus_isolate_2179-00_segment_S_nucleocapsid_protei .....C.... .A..T..... ...C..G... ..T....... .CC....... T..G..G..G .....C.... ..........  [560]
#EF681809.1_Jamestown_Canyon_virus_isolate_779-98_segment_S_nucleocapsid_protein ..A..C..C. .G..T..... ...C...... ..T....... ..C....... .T.......G .....C.... .A........  [560]
#EF681810.1_Jamestown_Canyon_virus_isolate_810-98_segment_S_nucleocapsid_protein ..A..C..C. .G..T..... ...C...... ..T....... ..C.C..... .T.......G .....C.... .A........  [560]
#EF681811.1_Jamestown_Canyon_virus_isolate_811-00_segment_S_nucleocapsid_protein .....C.... .A..T..... ...C..G... ..T....... .CC....... T..G..G..G .....C.... ..........  [560]
#EF681812.1_Jamestown_Canyon_virus_isolate_1697-03_segment_S_nucleocapsid_protei ..A..C..C. .G..T..... ...C...... ..T....... ..C....... .T.......G .....C.... .A........  [560]
#EF681813.1_Jamestown_Canyon_virus_isolate_1425-02_segment_S_nucleocapsid_protei ..A..C..C. .G..T..... ...C...... ..T....... ..C....... .T.......G .....C.... .A........  [560]
#EF681814.1_Jamestown_Canyon_virus_isolate_1441-04_segment_S_nucleocapsid_protei ..A..C..C. .G..T..... ...C...... ..T....... ..C....... .T.......G .....C.... .A........  [560]
#EF681815.1_Jamestown_Canyon_virus_isolate_928-00_segment_S_nucleocapsid_protein ..A..C..C. .G..T..... ...C...... ..T....... ..C....... .T.......G .....C.... .A........  [560]
#EF681816.1_Jamestown_Canyon_virus_isolate_1064-03_segment_S_nucleocapsid_protei .....C.... .G..T..... ...C..G... ..T....... ..C....... ......G..G ..C..C.... .A........  [560]
#EF681817.1_Jamestown_Canyon_virus_isolate_1369-02_segment_S_nucleocapsid_protei .....C.... .A..T..... ...C..G... ..T....... .CC....... T..G..G..G .....C.... ..........  [560]
#EF681818.1_Jamestown_Canyon_virus_isolate_1627-04_segment_S_nucleocapsid_protei .....C.... .A..T..... ...C..G... ..T....... .CC....... T..G..G..G .....C.... ..........  [560]
#EF681819.1_Jamestown_Canyon_virus_isolate_1810-02_segment_S_nucleocapsid_protei .....C.... .A..T..... ...C..G... ..T....... .CC....... T..G..G..G .....C.... ..........  [560]
#EF681820.1_Jamestown_Canyon_virus_isolate_2384-98_segment_S_nucleocapsid_protei .....C.... .A..T..... ...C..G... ..T....... .CC....... T..G..G..G .....C.... ..........  [560]
#EF681821.1_Jamestown_Canyon_virus_isolate_2707-01_segment_S_nucleocapsid_protei ..A..C..C. .G..T..... ...C...... ..T....... ..C....... .T.......G .....C.... .A........  [560]
#EF681822.1_Jamestown_Canyon_virus_isolate_2718-01_segment_S_nucleocapsid_protei .....C.... .A..T..... ...C..G... ..T....... .CC....... T..G..G..G .....C.... ..........  [560]
#EF681823.1_Jamestown_Canyon_virus_isolate_3280-03_segment_S_nucleocapsid_protei ..A..C..C. .G..T..... ...C...... ..T....... ..C....... .T.......G .....C.... .A........  [560]
#EF681824.1_Jamestown_Canyon_virus_isolate_3324-04_segment_S_nucleocapsid_protei .....C.... .A..T..... ...C..G... ..T....... .CC....... T..G..G..G .....C.... ..........  [560]
#EF681825.1_Jamestown_Canyon_virus_isolate_3573-03_segment_S_nucleocapsid_protei ..A..C..C. .G..T..... ...C...... ..T....... ..C.C..... .T.......G .....C.... .A........  [560]
#EF681826.1_Jamestown_Canyon_virus_isolate_3682-00_segment_S_nucleocapsid_protei ..A..C..C. .G..T..... ...C...... ..T....... ..C....... .T.......G .....C.... .A........  [560]
#EF681827.1_Jamestown_Canyon_virus_isolate_4148-03_segment_S_nucleocapsid_protei .....C.... .G..T..... ...C..G... ..T....... ..C....... ......G..G ..C..C.... .A........  [560]
#EF681828.1_Jamestown_Canyon_virus_isolate_4473-00_segment_S_nucleocapsid_protei .....C.... .G..T..... ...C..G... ..T....... ..C....... ......G..G ..C..C.... .A........  [560]
#EF681829.1_Jamestown_Canyon_virus_isolate_4742-04_segment_S_nucleocapsid_protei .....C.... .A..T..... ...C..G... ..T....... .CC....... T..G..G... .....C.... ..........  [560]
#EF681830.1_Jamestown_Canyon_virus_isolate_2274-05_segment_S_nucleocapsid_protei .....C.... .A..T..... ...C..G... ..T....... .CC....... T..G..G..G .....C.... ..........  [560]
#EF681831.1_Jamestown_Canyon_virus_isolate_1472-05_segment_S_nucleocapsid_protei .....C.... .A..T..... ...C..G... ..T....... .CC....... T..G..G..G .....C.... ..........  [560]
#EF681832.1_Jamestown_Canyon_virus_isolate_4910-02_segment_S_nucleocapsid_protei ..A..C..C. .G..T..... ...C...... ..T....... ..C....... .T.......G .....C.... .A........  [560]
#EF681833.1_Jamestown_Canyon_virus_isolate_275-01_segment_S_nucleocapsid_protein .....C.... .A..T..... ...C..G... ..T....... .CC....... T..G..G..G .....C.... ..........  [560]
#EF681834.1_Jamestown_Canyon_virus_isolate_339-05_segment_S_nucleocapsid_protein .....C.... .A..T..... ...C..G... ..T....... .CC....... T..G..G..G .....C.... ..........  [560]
#EF681835.1_Jamestown_Canyon_virus_isolate_3836-05_segment_S_nucleocapsid_protei .....C.... .A..T..... ...C..G... ..T....... .CC....... T..G..G..G .....C.... ..........  [560]
#EF681836.1_Jamestown_Canyon_virus_isolate_2286-00_segment_S_nucleocapsid_protei ..A..C..C. .G..T..... ...C...... ..T....... ..C.C..... .T.......G .....C.... .A........  [560]
#EF681837.1_Jamestown_Canyon_virus_isolate_1044-05_segment_S_nucleocapsid_protei .....C.... .A..T..... ...C..G... ..T....... .CC....... T..G..G..G .....C.... ..........  [560]
#EF681838.1_Jamestown_Canyon_virus_isolate_978-99_segment_S_nucleocapsid_protein .....C.... .A..T..... ...C..G... ..T....... .CC....... T..G..G... .....C.... ..........  [560]
#EF681839.1_Jamestown_Canyon_virus_isolate_4832-01_segment_S_nucleocapsid_protei .....C.... .A..T..... ...C..G... ..T....... .CC....... T..G..G..G .....C.... ..........  [560]
#EF681841.1_Jamestown_Canyon_virus_isolate_7101-03_segment_S_nucleocapsid_protei .....C.... .A..T..... ...C..G... ..T....... .CC....... T..G..G..G .....C.... ..........  [560]
#EF681842.1_Jamestown_Canyon_virus_isolate_Simsbury_segment_S_nucleocapsid_prote .....C.... .A..T..... ...C..G... ..T....... .CC....... T..G..G..G .....C.... ..........  [560]
#EF681843.1_Jamestown_Canyon_virus_isolate_8011-03_segment_S_nucleocapsid_protei .....C.... .A..T..... ...C..G... ..T....... .CC....... T..G..G..G .....C.... ..........  [560]
#EF681844.1_Jamestown_Canyon_virus_isolate_8536-03_segment_S_nucleocapsid_protei ..A..C..C. .G..T..... ...C...... ..T....... ..C....... .T.......G .....C.... .A........  [560]
#EF681845.1_Jamestown_Canyon_virus_isolate_11497-03_segment_S_nucleocapsid_prote .....C.... .A..T..... ...C..G... ..T....... .CC....... T..G..G..G .....C.... ..........  [560]
#EF681846.1_Jamestown_Canyon_virus_isolate_13995-03_segment_S_nucleocapsid_prote .....C.... .A..T..... ...C..G... ..T....... .CC....... T..G..G..G .....C.... ..........  [560]
#EF681847.1_Jamestown_Canyon_virus_isolate_1768-98_segment_S_nucleocapsid_protei ..A..C..C. .G..T..... ...C...... ..T....... ..C....... .T.......G .....C.... .A........  [560]
#EF681848.1_Jamestown_Canyon_virus_isolate_1385-06_segment_S_nucleocapsid_protei .....C.... .A..T..... ...C..G... ..T....... .CC....... T..G..G..G .....C.... ..........  [560]
#EF681849.1_Jamestown_Canyon_virus_isolate_2989-06_segment_S_nucleocapsid_protei .....C.... .A..T..... ...C..G... ..T....... .CC....... T..G..G..G .....C.... ..........  [560]
```

```
#EF681850.1_Jamestown_Canyon_virus_isolate_3381-06_segment_S_nucleocapsid_protei .....C.... .A..T..... ...C..G... ..T....... .CC....... T..G..G..G .....C.... .......... [560]
#EF681851.1_Jamestown_Canyon_virus_isolate_4095-06_segment_S_nucleocapsid_protei .....C.... .A..T..... ...C..G... ..T....... .CC....... T..G..G..G .....C.... .......... [560]
#EF681852.1_Jamestown_Canyon_virus_isolate_4078-06_segment_S_nucleocapsid_protei .....C.... .A..T..... ...C..G... ..T....... .CC....... T..G..G..G .....C.... .......... [560]
#EF681853.1_Jamestown_Canyon_virus_isolate_11-92_segment_S_nucleocapsid_protein_ ..A..C..C. .G..T..... ...C...... ..T....... ..C....... .T.......G .....C.... .A........ [560]
#EF681854.1_Jamestown_Canyon_virus_isolate_23-97_segment_S_nucleocapsid_protein_ ..A..C..C. .G..T..... ...C...... ..T....... ..C....... .T.......G .....C.... .A........ [560]
#EF681855.1_Jamestown_Canyon_virus_isolate_25-97_segment_S_nucleocapsid_protein_ .....C.... .A..T..... ...C..G... ..T....... .CC....... T..G..G..G .....C.... .......... [560]
#EF681856.1_Jamestown_Canyon_virus_isolate_29-97_segment_S_nucleocapsid_protein_ ..A..C..C. .G..T..... ...C...... ..T....... ..C....... .T.......G .....C.... .A........ [560]
#EF681857.1_Jamestown_Canyon_virus_isolate_423-99_segment_S_nucleocapsid_protein .....C.... .A..T..... ...C..G... ..T....... .CC....... T..G..G... .....C.... .......... [560]
#EF681858.1_Jamestown_Canyon_virus_isolate_1262-98_segment_S_nucleocapsid_protei ..A..C..C. .G..T..... ...C...... ..T....... ..C.C..... .T.......G .....C.... .A........ [560]
#EF681859.1_Jamestown_Canyon_virus_isolate_3438-06_segment_S_nucleocapsid_protei .....C.... .A..T..... ...C..G... ..T....... .CC....... T..G..G... .....C.... .......... [560]
#HM007350.1_Jamestown_Canyon_virus_strain_61V2235_nucleoprotein_and_NSs_protein_ ..A..C..C. .G..T..... ...C...... ..T....... ..C.C..... .T.G..G..G .....C..G. .A........ [560]
#HM007353.1_Jamestown_Canyon_virus_strain_3573-03_nucleoprotein_gene_complete_cd ..A..C..C. .G..T..... ...C...... ..T....... ..C.C..... .T.......G .....C.... .A........ [560]
#HM007356.1_Jamestown_Canyon_virus_strain_3324-04_nucleoprotein_and_NSs_protein_ .....C.... .A..T..... ...C..G... ..T....... .CC....... T..G..G..G .....C.... .......... [560]
#KM215518.1_Jamestown_Canyon_virus_isolate_F1819_nucleocapsid_protein_and_nonstr .....C.... .A..T..... ...C..G... ..T....... .CC.G..... T..G..G..G .....C.... .......... [560]
#KM215519.1_Jamestown_Canyon_virus_isolate_F6626_nucleocapsid_protein_and_nonstr .....C.... .A..T..... ...C..G... ..T....... .CC.G..... T..G..G..G .....C.... .......... [560]
#KM215520.1_Jamestown_Canyon_virus_isolate_ND0283_nucleocapsid_protein_and_nonst .....C.... .A..T..... ...C..G... ..T....... .CC.A..... T..G..G..G .....C.... .......... [560]
#KM215521.1_Jamestown_Canyon_virus_isolate_ND6194_nucleocapsid_protein_and_nonst .....C.... .A..T..... ...C..G... ..T....... .CC.G..... T..G..G..G .....C.... .......... [560]
#KM215522.1_Jamestown_Canyon_virus_isolate_W6701_nucleocapsid_protein_and_nonstr .....C.... .A..T..... ...C..G... ..T....... .CC.G..... T..G..G..G .....C.... .......... [560]
#KM215523.1_Jamestown_Canyon_virus_isolate_W14530_nucleocapsid_protein_and_nonst .....C.... .A..T..... ...C..G... ..T....... .CC.G..... T..G..G..G .....C..G. .......... [560]
#KM215524.1_Jamestown_Canyon_virus_isolate_W16690_nucleocapsid_protein_and_nonst .....C.... .A..T..... ...C..G... ..T....... .CC.G..... T..G..G..G .....C..G. .......... [560]
#KM215525.1_Jamestown_Canyon_virus_isolate_W18699_nucleocapsid_protein_and_nonst ..A..C.... .A..T..... ...C...... ..T....... ..C.C..... T..G..G..G ..C..C..G. .A........ [560]
#KM215526.1_Jamestown_Canyon_virus_isolate_W15316_nucleocapsid_protein_and_nonst .....C.... .A..T..... ...C..G... ..T....... .CC.G..... T..G..G..G .....C.... .......... [560]
#KM215527.1_Jamestown_Canyon_virus_isolate_W17680_nucleocapsid_protein_and_nonst .....C..A. .A..T..... ...C..G... ..T....... .CC.G..... T..G..G..G .....C.... .......... [560]
#KM215528.1_Jamestown_Canyon_virus_isolate_W19543_nucleocapsid_protein_and_nonst .....C.... .A..T..... ...C..G... ..T....... .CC.G..... T..G..G..G .....C.... .......... [560]
#KM215529.1_Jamestown_Canyon_virus_isolate_W19925_nucleocapsid_protein_and_nonst .....C.... .A..T..... ...C..G... ..T....... .CC.G..... T..G..G..G .....C..G. .......... [560]
#KM215530.1_Jamestown_Canyon_virus_isolate_W20764_nucleocapsid_protein_and_nonst .....C.... .A..T..... ...C..G... ..T....... .CC.G..... T..G..G..G .....C.... .......... [560]
#KM215531.1_Jamestown_Canyon_virus_isolate_W22352_nucleocapsid_protein_and_nonst .....C.... .T..T..... ...C..G... ..T....... .CC.G..... T..G..G..G .....C.... .......... [560]
#KM215532.1_Jamestown_Canyon_virus_isolate_F1829_nucleocapsid_protein_and_nonstr .....C.... .A..T..... ...C..G... ..T....... .CC.G..... T..G..G..G .....C.... .......... [560]
#KM215533.1_Jamestown_Canyon_virus_isolate_F6228_nucleocapsid_protein_and_nonstr .....C.... .A..T..... ...C..G... ..T....... .CC.G..... T..G..G..G .....C.... .......... [560]
#KM215534.1_Jamestown_Canyon_virus_isolate_F6235_nucleocapsid_protein_and_nonstr .....C.... .A..T..... ...C..G... ..T....... .CC.G..... T..G..G..G .....C.... .......... [560]
#KM215535.1_Jamestown_Canyon_virus_isolate_F10095_nucleocapsid_protein_and_nonst .....C.... .A..T..... ...C..G... ..T....... .CC.G..... T..G..G..G .....C.... .......... [560]
#KM215536.1_Jamestown_Canyon_virus_isolate_F13418_nucleocapsid_protein_and_nonst .....C.... .A..T..... ...C..G... ..T....... .CC.G..... T..G..G..G .....C.... .......... [560]
#KM215537.1_Jamestown_Canyon_virus_isolate_F14162_nucleocapsid_protein_and_nonst .....C.... .A..T..... ...C..G... ..T....... .CC.G..... T..G..G..G .....C..G. .......... [560]
#KM215538.1_Jamestown_Canyon_virus_isolate_F14183_nucleocapsid_protein_and_nonst .....C.... .A..T..... ...C..G... ..T....... .CC.G..... T..G..G..G .....C..G. .......... [560]
#KM215539.1_Jamestown_Canyon_virus_isolate_F14278_nucleocapsid_protein_and_nonst .....C.... .A..T..... ...C..G... ..T....... .CC.A..... T..G..G..G .....T.... .......... [560]
#KM215540.1_Jamestown_Canyon_virus_isolate_F16109_nucleocapsid_protein_and_nonst .....C.... .A..T..... ...C..G... ..T....... .CC.G..... T..G..G..G .....C.... .......... [560]
#KM215541.1_Jamestown_Canyon_virus_isolate_W8270_nucleocapsid_protein_and_nonstr .....C.... .A..T..... ...C..G... ..T....... .CC.G..... T..G..G..G .....C.... .......... [560]
#KM215542.1_Jamestown_Canyon_virus_isolate_W16455_nucleocapsid_protein_and_nonst .....C.... .A..T..... ...C..G... ..T....... .CC.G..... T..G..G..G .....C..G. .......... [560]
#KM215543.1_Jamestown_Canyon_virus_isolate_W18738_nucleocapsid_protein_and_nonst .....C.... .A..T..... ...C..G... ..T....... .CC.G..... T..G..G..G .....C.... .......... [560]
#KM215544.1_Jamestown_Canyon_virus_isolate_F4276_nucleocapsid_protein_and_nonstr .....C.... .A..T..... ...C..G... ..T....... .CC.G..... T..G..G..G .....C.... .......... [560]
#KM215545.1_Jamestown_Canyon_virus_isolate_W19962_nucleocapsid_protein_and_nonst .....C.... .A..T..... ...C..G... ..T....... .CC.G..... T..G..G..G .....C..G. .......... [560]
#KM215546.1_Jamestown_Canyon_virus_isolate_F5609_nucleocapsid_protein_and_nonstr .....C.... .A..T..... ...T..G... ..T....... .CC.G..... T..G..G..G .....C.... .......... [560]
#KM215547.1_Jamestown_Canyon_virus_isolate_F7826_nucleocapsid_protein_and_nonstr .....C.... .A..T..... ...C..G... ..T....... .CC.G..... T..G..G..G .....C.... .......... [560]
#KM215548.1_Jamestown_Canyon_virus_isolate_F7932_nucleocapsid_protein_and_nonstr .....C.... .A..T..... ...C...... ..T....... .CC.G..... T..G..G..G .....C.... .......... [560]
#KM215549.1_Jamestown_Canyon_virus_isolate_F8532_nucleocapsid_protein_and_nonstr .....C.... .A..T..... ...C..G... ..T....... .CC.G..... T..G..G..G .....C.... .......... [560]
#KM215550.1_Jamestown_Canyon_virus_isolate_F10720_nucleocapsid_protein_and_nonst .....C.... .A..T..... ...C..G... ..T....... .CC.G..... T..G..G..G .....C.... .......... [560]
#KM215551.1_Jamestown_Canyon_virus_isolate_ND1300_nucleocapsid_protein_and_nonst .....C.... .A..T..... ...C..G... ..T....... .CC.G..... T..G..G..G .....C.... .......... [560]
#KM215552.1_Jamestown_Canyon_virus_isolate_W13135_nucleocapsid_protein_and_nonst .....C.... .T..T..... ...C..G... ..T....... .CC.G..... T..G..G..G .....C.... .......... [560]
#KM215553.1_Jamestown_Canyon_virus_isolate_W16456_nucleocapsid_protein_and_nonst .....C.... .A..T..... ...C..G... ..T....... .CC.G..... T..G..G..G .....C..G. .......... [560]
#KM215554.1_Jamestown_Canyon_virus_isolate_W16590_nucleocapsid_protein_and_nonst .....C.... .T..T..... ...C..G... ..T....... .CC.G..... T..G..G..G .....C.... .......... [560]
#KM215555.1_Jamestown_Canyon_virus_isolate_W19651_nucleocapsid_protein_and_nonst .....C.... .A..T..... ...C..G... ..T....... .CC.G..... T..G..G..G .....C.... .......... [560]
#KM215556.1_Jamestown_Canyon_virus_isolate_F3406_nucleocapsid_protein_and_nonstr .....C.... .A..T..... ...C..G... ..T....... .CC.G..... T..G..G..G .....C.... .......... [560]
#KM215557.1_Jamestown_Canyon_virus_isolate_F6577_nucleocapsid_protein_and_nonstr .....C.... .A..T..... ...C..G... ..T....... .CC.G..... T..G..G..G .....C.... .......... [560]
#KM215558.1_Jamestown_Canyon_virus_isolate_ND374_nucleocapsid_protein_and_nonstr .....C.... .A..T..... ...C..G... ..T....... .CC.G..... ...G..G..G .....C.... .......... [560]
#KM215559.1_Jamestown_Canyon_virus_isolate_W18393_nucleocapsid_protein_and_nonst ..A..C.... .A..T..... ...C...... ..T....... ..C.C..... T..G..G..G ..C..C..G. .A........ [560]
#KM215560.1_Jamestown_Canyon_virus_isolate_W18901_nucleocapsid_protein_and_nonst ..A..C.... .A..T..... ...C...... ..T....... ..C.C..... T..G..G..G ..C..C..G. .A........ [560]
#KX817317.1_Jamestown_Canyon_virus_strain_61V2235_segment_S_complete_sequence    ..A..C..C. .G..T..... ...C...... ..T....... ..C.C..... .T.G..G..G .....C..G. .A........ [560]
```

```
#MH370817.1_Jamestown_Canyon_virus_isolate_L36708_segment_S_complete_sequence    ..A..C..C. .G..T..... ...C...... ..T....... ..C....... .T.......G ....C..... .A........  [560]
#MH370820.1_Jamestown_Canyon_virus_isolate_MN256-260_segment_S_complete_sequence ....C..... .A..T..... ...C..G... ..T....... .CC.G..... T..G..G..G .......... ..........  [560]
#U12799.1_Jamestown_Canyon_virus_DAV28_S_RNA_segment_N_and_NSs_protein_genes_com ..A..C..C. .G..T..... ...C...... ..T....... ..C.C..... .T.G..G..G ....C..G.. .A........  [560]
#U12796.1_Jamestown_Canyon_virus_61v2235_S_RNA_segment_N_and_NSs_protein_genes_c ..A..C..C. .G..T..... ...C...... ..T....... ..C.C..... .T.G..G..G ....C..G.. .A........  [560]
#KM215561.1_Jamestown_Canyon_virus_isolate_W23697_nucleocapsid_protein_and_nonst ....C..... .A..T..... ...C..G... ..T....... .CC.G..... T..G..G..G ....C..G.. ..........  [560]
#KT288271.1_Inkoo_virus_strain_LEIV-15248Iv_segment_S_nucleoprotein_(N)_gene_com ..A..C.... .A..T..... ...C..G... ..T....... ..C....... ...G..G..G ..A..T..G. .A........  [560]
#KT288274.1_Inkoo_virus_strain_LEIV-18154Yak_segment_S_nucleoprotein_(N)_gene_co ..A..C..A. .A..T..... ...C...... ..T....... ..C....... ...G..G..G ..A..T..G. .A........  [560]
#KT288275.1_Inkoo_virus_strain_LEIV-9874Kar_segment_S_nucleoprotein_(N)_gene_com ..A..C.... .A..T..... ...C..G... ..T....... ..C....... ...G..G..G ..A..T..G. .A........  [560]
#KT288277.1_Inkoo_virus_strain_LEIV-18784Yak_segment_S_nucleoprotein_(N)_gene_co ..A..C..A. .A..T..... ...C...... ..T....... ..C....... ...G..G..G ..A..T..G. .A........  [560]
#KT288280.1_Inkoo_virus_strain_LEIV-22780Tyum_segment_S_nucleoprotein_(N)_gene_c ..A..C..A. .A..T..... ...C..G... ..C....... ..C....... ...G..G..G ..A..T..G. .A........  [560]
#KT288283.1_Inkoo_virus_strain_LEIV-18152Yak_segment_S_nucleoprotein_(N)_gene_co ..A..C..A. .A..T..... ...C...... ..T....... ..C....... ...G..G..G ..A..T..G. .A........  [560]
#KT288286.1_Inkoo_virus_strain_LEIV-21643Kra_segment_S_nucleoprotein_(N)_gene_co ..A..C.... .A..T..... ...C..G... ..T....... ..C....... ...G..G..G ..A..T..G. .A..T.....  [560]
#KX554935.1_Inkoo_virus_strain_Lovanger_nucleocapsid_protein_and_nonstructural_p ..A..C.... .A..T..... ...C..G... ..T....... ..C....... ...G..G..G ..A..T..G. .A........  [560]
#U47137.1_Inkoo_virus_Prototype_KN3641_nucleocapsid_protein_and_non-structural_p ..A..C.... .A..T..... ...C..G... ..T....... ..C....... ......G..G ..A..T..G. .A..T.....  [560]
#U47138.1_Inkoo_virus_SW_AR_83-161_nucleocapsid_protein_and_non-structural_prote ..A..C.... .G..T..... ...C..G... ..T....... ..C....... ...G..G..G ..A..T..G. .A.....C..  [560]
#Z68496.1_Inkoo_virus_RNA_for_N_protein_and_RNA_for_NS_protein_strain_KN_3641    ..A..C.... .A..T..... ...C..G... ..T....... ..C....... ......G..G ..A..T..G. .A..T.....  [560]
```

```
#KT630290.1_Keystone_virus_strain_KEYV/Ochlerotatus_atlanticus/USA/KEYVLK01/2005 GACATTGACT GCAGACAAAT GGATGGCTCA AAAGACATCT ATGATTACAA AGAGCCTCAA AGATGTAGAG CAGCTCAAGT  [640]
#KT630293.1_Keystone_virus_strain_KEYV/Ochlerotatus_atlanticus/USA/KEYVLK02/2005 .......... .....T.... .......... .......... .......... .......... ......G... .....T....  [640]
#MH016786.1_Keystone_virus_strain_KEYV/Homo_sapiens/Gainesville-1/2016_nucleopro .......... .......... .......... .......... .......... .......... .......... ..........  [640]
#KX817323.1_Keystone_virus_strain_B64-5587.05_segment_S_complete_sequence        .......... .......... .......... .......... .......... .......... .......... ..........  [640]
#MG821231.1_Keystone_virus_isolate_AR14033_segment_S_complete_sequence           ......A... .......... .......... ...A...... ......G... .......... ...AA.T... ..........  [640]
#MG765471.1_Keystone_virus_isolate_AVA1709441_nucleocapsid_and_NSs_genes_complet ......A... ........G. .......... ...A...... ......G... .......... ...AA.T... ..........  [640]
#KX817329.1_Melao_virus_strain_TRVL_9375_segment_S_complete_sequence             C..C...... .......... .......A.. ...A..CA.. ......G.T. .A.....G.. G......... .....G....  [640]
#KX817335.1_Serra_do_Navio_virus_strain_BeAr_103645_segment_S_complete_sequence  A..CC....A ........G. .......A.. ...A..TGT. T.A..C.... .A.....A.. G.....T... ...T.G....  [640]
#KX817320.1_Jerry_Slough_virus_strain_BFS_4474_segment_S_complete_sequence       C..C...... .....G.... .......A.. G......GTG C.C...G.T. .....T.G.. G.....T... ........A.  [640]
#KX817338.1_South_River_virus_strain_NJO-94F_segment_S_complete_sequence         C..C.....G ..T..A.... .......A.. G......GTG C.C...G.C. .......A.. G.....G... .....T..A.  [640]
#GU018050.2_South_River_virus_isolate_SORV-252_nucleoprotein_and_NSs_protein_gen C..C..A... ..C..A.... .......A.. ...A...GTA C.T...G.C. .A...T.A.. ......G... .....T..A.  [640]
#EF681804.1_Jamestown_Canyon_virus_isolate_5592-02_segment_S_nucleocapsid_protei C..C...... .....G..G. .......A.. G......GTG C.C...G.T. ....T..A.. G........A .....T..A.  [640]
#EF681805.1_Jamestown_Canyon_virus_isolate_368-99_segment_S_nucleocapsid_protein C..C...... .....G..G. .......A.. G......GTG C.C...G.T. ....T..A.. G........A .....T..A.  [640]
#EF681806.1_Jamestown_Canyon_virus_isolate_6163-03_segment_S_nucleocapsid_protei C..C...... .....G..G. .......A.. G......GTG C.C...G.T. ....T..A.. G........A .....T..A.  [640]
#EF681807.1_Jamestown_Canyon_virus_isolate_468-04_segment_S_nucleocapsid_protein C..C...... .....G..G. .......A.. G......GTG C.C...G.C. ....T..A.. G.....T..A ..A.....A.  [640]
#EF681808.1_Jamestown_Canyon_virus_isolate_2179-00_segment_S_nucleocapsid_protei C.TC...... .....G..G. .......A.. G......GTG C.C...G.T. ....T..A.. G........A .....T..A.  [640]
#EF681809.1_Jamestown_Canyon_virus_isolate_779-98_segment_S_nucleocapsid_protein C..C...... .....G..G. .......A.. G......GTG C.C...G.C. ....T..A.. G.....T..A ..A.....A.  [640]
#EF681810.1_Jamestown_Canyon_virus_isolate_810-98_segment_S_nucleocapsid_protein C..C...... .....G..G. .......A.. G......GTG C.C...G.C. ....T..A.. G.....T..A ..A.....A.  [640]
#EF681811.1_Jamestown_Canyon_virus_isolate_811-00_segment_S_nucleocapsid_protein C..C...... .....G..G. .......A.. G......GTG C.C...G.T. .......A.. G........A .....T..A.  [640]
#EF681812.1_Jamestown_Canyon_virus_isolate_1697-03_segment_S_nucleocapsid_protei C..C...... .....G..G. .......A.. G......GTG C.C...G.C. ....T..A.. G.....T..A ..A.....A.  [640]
#EF681813.1_Jamestown_Canyon_virus_isolate_1425-02_segment_S_nucleocapsid_protei C..C...... .....G..G. .......A.. G......GTG C.C...G.C. ....T..A.. G.....T..A ..A.....A.  [640]
#EF681814.1_Jamestown_Canyon_virus_isolate_1441-04_segment_S_nucleocapsid_protei C..C...... .....G..G. .......A.. G......GTG C.C...G.C. ....T..A.. G.....T..A ..A.....A.  [640]
#EF681815.1_Jamestown_Canyon_virus_isolate_928-00_segment_S_nucleocapsid_protein C..C...... .....G..G. .......A.. G......GTG C.C...G.C. ....T..A.. G.....T..A ..A.....A.  [640]
#EF681816.1_Jamestown_Canyon_virus_isolate_1064-03_segment_S_nucleocapsid_protei C..C...... .....G..G. .......A.. G......GTG C.C...G.T. ....T..A.. G.....T... ........A.  [640]
#EF681817.1_Jamestown_Canyon_virus_isolate_1369-02_segment_S_nucleocapsid_protei C..C..A... .....G..G. .......A.. G......GTG C.C...G.T. ....T..A.. G........A .....T..A.  [640]
#EF681818.1_Jamestown_Canyon_virus_isolate_1627-04_segment_S_nucleocapsid_protei C..C...... .....G..G. .......A.. G......GTG C.T...G.T. ....T..A.. G........A .....T..A.  [640]
#EF681819.1_Jamestown_Canyon_virus_isolate_1810-02_segment_S_nucleocapsid_protei C..C...... .....G..G. .......A.. G......GTG C.C...G.T. ....T..A.. G........A .....T..A.  [640]
#EF681820.1_Jamestown_Canyon_virus_isolate_2384-98_segment_S_nucleocapsid_protei C..C...... .....G..G. .......G.. G......GTG C.C...G.T. ....T..A.. G........A .....T..A.  [640]
#EF681821.1_Jamestown_Canyon_virus_isolate_2707-01_segment_S_nucleocapsid_protei T..C...... .....G..G. .......A.. G......GTG C.C...G.C. ....T..A.. G.....T..A ..A.....A.  [640]
#EF681822.1_Jamestown_Canyon_virus_isolate_2718-01_segment_S_nucleocapsid_protei C..C...... .....G..G. .......A.. G......GTG C.C...G.T. ....T..A.. G........A .....T..A.  [640]
#EF681823.1_Jamestown_Canyon_virus_isolate_3280-03_segment_S_nucleocapsid_protei C..C...... .....G..G. .......A.. G......GTG C.C...G.C. ....T..A.. G.....T..A ..A.....A.  [640]
#EF681824.1_Jamestown_Canyon_virus_isolate_3324-04_segment_S_nucleocapsid_protei C..C...... .....G..G. .......A.. G......GTG C.C...G.T. ....T..A.. G........A .....T..A.  [640]
#EF681825.1_Jamestown_Canyon_virus_isolate_3573-03_segment_S_nucleocapsid_protei C..C...... .....G..G. .......A.. G......GTG C.C...G.T. ....T..A.. G.....T..A ..A.....A.  [640]
#EF681826.1_Jamestown_Canyon_virus_isolate_3682-00_segment_S_nucleocapsid_protei C..C...... .....G..G. .......A.. G......GTG C.C...G.C. ....T..A.. G.....T..A ..A.....A.  [640]
#EF681827.1_Jamestown_Canyon_virus_isolate_4148-03_segment_S_nucleocapsid_protei C..C...... .....G..G. .......A.. G......GTG C.C...G.T. ....T..A.. G.....T... ........A.  [640]
#EF681828.1_Jamestown_Canyon_virus_isolate_4473-00_segment_S_nucleocapsid_protei C..C...... .....G..G. .......A.. G......GTG C.C...G.T. ....T..A.. G.....T... ........A.  [640]
#EF681829.1_Jamestown_Canyon_virus_isolate_4742-04_segment_S_nucleocapsid_protei C..C...... .....G..G. .......A.. G......GTG C.C...G.T. ....T..A.. G........A .....T..A.  [640]
#EF681830.1_Jamestown_Canyon_virus_isolate_2274-05_segment_S_nucleocapsid_protei C..C...... .....G..G. .......A.. G......GTG C.C...G.T. ....T..A.. G........A .....T..A.  [640]
#EF681831.1_Jamestown_Canyon_virus_isolate_1472-05_segment_S_nucleocapsid_protei C..C...... .....G..G. .......A.. G......GTG C.C...G.T. ....T..A.. G........A .....T..A.  [640]
#EF681832.1_Jamestown_Canyon_virus_isolate_4910-02_segment_S_nucleocapsid_protei C..C...... .....G..G. .......A.. G......GTG C.C...G.C. ....T..A.. G.....T..A ..A.....A.  [640]
#EF681833.1_Jamestown_Canyon_virus_isolate_275-01_segment_S_nucleocapsid_protein C..C...... .....G..G. .......A.. G......GTG C.C...G.T. ....T..A.. G........A .....T..A.  [640]
#EF681834.1_Jamestown_Canyon_virus_isolate_339-05_segment_S_nucleocapsid_protein C..C...... .....G..G. .......A.. G......GTG C.C...G.T. ....T..A.. G........A .....T..A.  [640]
#EF681835.1_Jamestown_Canyon_virus_isolate_3836-05_segment_S_nucleocapsid_protei C..C...... .....G..G. .......A.. G......GTG C.C...G.T. ....T..A.. G........A .....T..A.  [640]
#EF681836.1_Jamestown_Canyon_virus_isolate_2286-00_segment_S_nucleocapsid_protei C..C...... .....G..G. .......A.. G......GTG C.C...G.C. ....T..A.. G.....T..A ..A.....A.  [640]
#EF681837.1_Jamestown_Canyon_virus_isolate_1044-05_segment_S_nucleocapsid_protei C..C...... .....G..G. .......A.. G......GTG C.C...G.T. ....T..A.. G........A .....T..A.  [640]
#EF681838.1_Jamestown_Canyon_virus_isolate_978-99_segment_S_nucleocapsid_protein C..C...... .....G..G. .......A.. G......GTG C.C...G.T. ....T..A.. G........A .....T..A.  [640]
#EF681839.1_Jamestown_Canyon_virus_isolate_4832-01_segment_S_nucleocapsid_protei C.TC...... .....G..G. .......A.. G......GTG C.C...G.T. ....T..A.. G........A .....T..A.  [640]
#EF681841.1_Jamestown_Canyon_virus_isolate_7101-03_segment_S_nucleocapsid_protei C..C...... .....G..G. .......A.. G......GTG C.C...G.T. ....T..A.. G........A .....T..A.  [640]
#EF681842.1_Jamestown_Canyon_virus_isolate_Simsbury_segment_S_nucleocapsid_prote C..C...... .....G..G. .......A.. G......GTG C.C...G.T. ....T..A.. G........  .....T..A.  [640]
#EF681843.1_Jamestown_Canyon_virus_isolate_8011-03_segment_S_nucleocapsid_protei C..C...... .....G..G. .......G.. G......GTG C.C...G.T. ....T..A.. G........A .....T..A.  [640]
#EF681844.1_Jamestown_Canyon_virus_isolate_8536-03_segment_S_nucleocapsid_protei C..C...... .....G..G. .......A.. G......GTG C.C...G.C. ....T..A.. G.....T..A ..A.....A.  [640]
#EF681845.1_Jamestown_Canyon_virus_isolate_11497-03_segment_S_nucleocapsid_prote C..C...... .....G..G. .......A.. G......GTG C.C...G.T. ....T..A.. G........A .....T..A.  [640]
#EF681846.1_Jamestown_Canyon_virus_isolate_13995-03_segment_S_nucleocapsid_prote C..C...... .....G..G. .......A.. G......GTG C.C...G.T. ....T..A.. G........A .....T..A.  [640]
#EF681847.1_Jamestown_Canyon_virus_isolate_1768-98_segment_S_nucleocapsid_protei C..C...... .....G..G. .......A.. G......GTG C.C...G.C. ....T..A.. G.....T..A ..A.....A.  [640]
#EF681848.1_Jamestown_Canyon_virus_isolate_1385-06_segment_S_nucleocapsid_protei C..C...... .....G..G. .......A.. G......GTG C.C...G.T. ....T..A.. G........A .....T..A.  [640]
#EF681849.1_Jamestown_Canyon_virus_isolate_2989-06_segment_S_nucleocapsid_protei C..C...... .....G..G. .......A.. G......GTG C.C...G.T. ....T..A.. G........A .....T..A.  [640]
```

#EF681850.1_Jamestown_Canyon_virus_isolate_3381-06_segment_S_nucleocapsid_protei C.TC...... .....G..G. ....A.. G......GTG C.C...G.T. ....T..A.. G........A .....T..A. [640]
#EF681851.1_Jamestown_Canyon_virus_isolate_4095-06_segment_S_nucleocapsid_protei C..C...... .....G..G. ....A.. G......GTG C.C...G.T. ....T..A.. G........A .....T..A. [640]
#EF681852.1_Jamestown_Canyon_virus_isolate_4078-06_segment_S_nucleocapsid_protei C..C...... .....G..G. ....A.. G......GTG C.C...G.T. ....T..A.. G........A .....T..A. [640]
#EF681853.1_Jamestown_Canyon_virus_isolate_11-92_segment_S_nucleocapsid_protein_ C..C...... .....G..G. ....A.. G......GTG C.C...G.C. ....T..A.. G.....T..A ..A.....A. [640]
#EF681854.1_Jamestown_Canyon_virus_isolate_23-97_segment_S_nucleocapsid_protein_ C..C...... .....G..G. ....A.. G......GTG C.C...G.C. ....T..A.. G.....T..A ..A.....A. [640]
#EF681855.1_Jamestown_Canyon_virus_isolate_25-97_segment_S_nucleocapsid_protein_ C..C...... .....G..G. ....A.. G......GTG C.C...G.T. ....T..A.. G........A .....T..A. [640]
#EF681856.1_Jamestown_Canyon_virus_isolate_29-97_segment_S_nucleocapsid_protein_ C..C...... .....G..G. ....A.. G......GTG C.C...G.C. ....T..A.. G.....T..A ..A.....A. [640]
#EF681857.1_Jamestown_Canyon_virus_isolate_423-99_segment_S_nucleocapsid_protein C..C...... .....G..G. ....A.. G......GTG C.C...G.T. ....T..A.. G........A .....T..A. [640]
#EF681858.1_Jamestown_Canyon_virus_isolate_1262-98_segment_S_nucleocapsid_protei C..C...... .....G..G. ....A.. G......GTG C.C...G.C. ....T..A.. G.....T..A ..A.....A. [640]
#EF681859.1_Jamestown_Canyon_virus_isolate_3438-06_segment_S_nucleocapsid_protei C..C...... .....G..G. ....A.. G......GTG C.C...G.T. ....T..A.. G........A .....T..A. [640]
#HM007350.1_Jamestown_Canyon_virus_strain_61V2235_nucleoprotein_and_NSs_protein_ C..C...... .....G..G. ....G.. G......GTG C.C...G.T. .....T.G.. G.....C... ....A. [640]
#HM007353.1_Jamestown_Canyon_virus_strain_3573-03_nucleoprotein_gene_complete_cd C..C...... .....G..G. ....A.. G......GTG C.C...G.T. ....T..A.. G.....T..A ..A.....A. [640]
#HM007356.1_Jamestown_Canyon_virus_strain_3324-04_nucleoprotein_and_NSs_protein_ C..C...... .....G..G. ....A.. G......GTG C.C...G.T. ....T..A.. G........A .....T..A. [640]
#KM215518.1_Jamestown_Canyon_virus_isolate_F1819_nucleocapsid_protein_and_nonstr C..T...... .....G..G. ....A.. G......GTG C.C...G.T. ....T..A.. G........A .....T..A. [640]
#KM215519.1_Jamestown_Canyon_virus_isolate_F6626_nucleocapsid_protein_and_nonstr C..T...... .....G..G. ....A.. G......GTG C.C...G.T. ....T..A.. G........A .....T..A. [640]
#KM215520.1_Jamestown_Canyon_virus_isolate_ND0283_nucleocapsid_protein_and_nonst C..C...... .....G..G. ....A.. G......GTG C.C...G.T. ....T..A.. G........A .....T..A. [640]
#KM215521.1_Jamestown_Canyon_virus_isolate_ND6194_nucleocapsid_protein_and_nonst C..C..A... .....G..G. ....A.. G......GTG C.C...G.T. ....T..A.. G........A .....T..A. [640]
#KM215522.1_Jamestown_Canyon_virus_isolate_W6701_nucleocapsid_protein_and_nonstr C..C...... .....G..G. ....A.. G..A...GTG C.C...G.T. ....T..A.. G........A .....T..A. [640]
#KM215523.1_Jamestown_Canyon_virus_isolate_W14530_nucleocapsid_protein_and_nonst C..C...... .....G..G. ....A.. G......GTG C.C...G.T. ....T..A.. G........A .....T..A. [640]
#KM215524.1_Jamestown_Canyon_virus_isolate_W16690_nucleocapsid_protein_and_nonst C..C...... .....G..G. ....A.. G......GTG C.C...G.T. ....T..A.. G........A .....T..A. [640]
#KM215525.1_Jamestown_Canyon_virus_isolate_W18699_nucleocapsid_protein_and_nonst C..C...... .....G.... ....A.. G......GTG C.C...G.T. .....T.G.. G.....T... ....A. [640]
#KM215526.1_Jamestown_Canyon_virus_isolate_W15316_nucleocapsid_protein_and_nonst C..C...... .....G..G. ....A.. ....GTG C.C...G.T. ....T..A.. G........A .....T..A. [640]
#KM215527.1_Jamestown_Canyon_virus_isolate_W17680_nucleocapsid_protein_and_nonst C..C..A... .....G..G. ....A.. G......GTG C.C...G.T. ....T..A.. G........A .....T..A. [640]
#KM215528.1_Jamestown_Canyon_virus_isolate_W19543_nucleocapsid_protein_and_nonst C..CC..... .....G..G. ....A.. G......GTG C.C...G.T. ....T..A.. G........A .....T..A. [640]
#KM215529.1_Jamestown_Canyon_virus_isolate_W19925_nucleocapsid_protein_and_nonst C..C...... .....G..G. ....A.. G......GTG C.C...G.T. .A..T..A.. G........A .....T..A. [640]
#KM215530.1_Jamestown_Canyon_virus_isolate_W20764_nucleocapsid_protein_and_nonst C..C...... .....G..G. ....A.. G......GTG C.C...G.T. ....T..A.. G........A .....T..A. [640]
#KM215531.1_Jamestown_Canyon_virus_isolate_W22352_nucleocapsid_protein_and_nonst C..C...... .....G..G. ....A.. G......GTG C.C...G.T. ....T..A.. G........A .....T..A. [640]
#KM215532.1_Jamestown_Canyon_virus_isolate_F1829_nucleocapsid_protein_and_nonstr C..T...... .....G..G. ....A.. G......GTG C.C...G.T. ....T..A.. G........A .....T..A. [640]
#KM215533.1_Jamestown_Canyon_virus_isolate_F6228_nucleocapsid_protein_and_nonstr C..T...... .....G..G. ....A.. G......GTG C.C...G.T. ....T..A.. G........A .....T..A. [640]
#KM215534.1_Jamestown_Canyon_virus_isolate_F6235_nucleocapsid_protein_and_nonstr C..T...... .....G..G. ....A.. G......GTG C.C...G.T. ....T..A.. G........A .....T..A. [640]
#KM215535.1_Jamestown_Canyon_virus_isolate_F10095_nucleocapsid_protein_and_nonst C..C..A... .....G..G. ....A.. G......GTG C.C...G.T. ....T..A.. G........A .....T..A. [640]
#KM215536.1_Jamestown_Canyon_virus_isolate_F13418_nucleocapsid_protein_and_nonst C..C...... .....G..G. ....A.. G......GTG C.C...G.T. ....T..A.. G........A .....T..A. [640]
#KM215537.1_Jamestown_Canyon_virus_isolate_F14162_nucleocapsid_protein_and_nonst C..C...... .....G..G. ....A.. G......GTG C.C...G.T. ....T..A.. G........A .....T..A. [640]
#KM215538.1_Jamestown_Canyon_virus_isolate_F14183_nucleocapsid_protein_and_nonst C..C...... .....G..G. ....A.. G......GTG C.C...G.T. ....T..A.. G........A .....T..A. [640]
#KM215539.1_Jamestown_Canyon_virus_isolate_F14278_nucleocapsid_protein_and_nonst C..C...... .....G..G. ....A.. G......GTG C.C...G.T. ....T..A.. G........A .....T..A. [640]
#KM215540.1_Jamestown_Canyon_virus_isolate_F16109_nucleocapsid_protein_and_nonst C..C..A... .....G..G. ....A.. G......GTG C.C...G.T. ....T..A.. G........A .....T..A. [640]
#KM215541.1_Jamestown_Canyon_virus_isolate_W8270_nucleocapsid_protein_and_nonstr C..T...... .....G..G. ....A.. G......GTG C.C...G.T. ....T..A.. G........A .....T..A. [640]
#KM215542.1_Jamestown_Canyon_virus_isolate_W16455_nucleocapsid_protein_and_nonst C..C...... .....G..G. ....A.. G......GTG C.C...G.T. ....T..A.. G........A .....T..A. [640]
#KM215543.1_Jamestown_Canyon_virus_isolate_W18738_nucleocapsid_protein_and_nonst C..C...... .....G..G. ....A.. G..A...GTG C.C...G.T. ....T..A.. G........A .....T..A. [640]
#KM215544.1_Jamestown_Canyon_virus_isolate_F4276_nucleocapsid_protein_and_nonstr C..C..A... .....G..G. ....A.. G......GTG C.C...G.T. ....T..A.. G........A .....T..A. [640]
#KM215545.1_Jamestown_Canyon_virus_isolate_W19962_nucleocapsid_protein_and_nonst C..C...... .....G..G. ....A.. G......GTG C.C...G.T. .A..T..A.. G........A .....T..A. [640]
#KM215546.1_Jamestown_Canyon_virus_isolate_F5609_nucleocapsid_protein_and_nonstr C..T...... .....G..G. ....A.. G......GTG C.C...G.T. ....T..A.. G........A .....T..A. [640]
#KM215547.1_Jamestown_Canyon_virus_isolate_F7826_nucleocapsid_protein_and_nonstr C..T...... .....G..G. ....A.. G......GTG C.C...G.T. ....T..A.. G........A .....T..A. [640]
#KM215548.1_Jamestown_Canyon_virus_isolate_F7932_nucleocapsid_protein_and_nonstr C..C...... .....G..G. ....A.. G......GTG C.C...G.T. ....T..A.. G........A .....T..A. [640]
#KM215549.1_Jamestown_Canyon_virus_isolate_F8532_nucleocapsid_protein_and_nonstr C..T...... .....G..G. ....A.. G......GTG C.C...G.T. ....T..A.. G........A .....T..A. [640]
#KM215550.1_Jamestown_Canyon_virus_isolate_F10720_nucleocapsid_protein_and_nonst C..T...... .....G..G. ....A.. G......GTG C.C...G.T. ....T..A.. G........A .....T..A. [640]
#KM215551.1_Jamestown_Canyon_virus_isolate_ND1300_nucleocapsid_protein_and_nonst C..C..A... .....G..G. ....A.. G......GTG C.C...G.T. ....T..A.. G........A .....T..A. [640]
#KM215552.1_Jamestown_Canyon_virus_isolate_W13135_nucleocapsid_protein_and_nonst C..T...... .....G..G. ....A.. G......GTG C.C...G.T. ....T..A.. G........A .....T..A. [640]
#KM215553.1_Jamestown_Canyon_virus_isolate_W16456_nucleocapsid_protein_and_nonst C..C...... .....G..G. ....A.. G......GTG C.C...G.T. ....T..A.. G........A .....T..A. [640]
#KM215554.1_Jamestown_Canyon_virus_isolate_W16590_nucleocapsid_protein_and_nonst C..T...... .....G..G. ....A.. G......GTG C.C...G.T. ....T..A.. G........A .....T..A. [640]
#KM215555.1_Jamestown_Canyon_virus_isolate_W19651_nucleocapsid_protein_and_nonst C..C..A... .....G..G. ....A.. G......GTG C.C...G.T. ....T..A.. G........A .....T..A. [640]
#KM215556.1_Jamestown_Canyon_virus_isolate_F3406_nucleocapsid_protein_and_nonstr C..C..A... .....G..G. ....A.. G......GTG C.C...G.T. ....T..A.. G........A .....T..A. [640]
#KM215557.1_Jamestown_Canyon_virus_isolate_F6577_nucleocapsid_protein_and_nonstr C..C...... .....G..G. ....A.. G......GTG C.C...G.T. ....T..A.. G........A .....T..A. [640]
#KM215558.1_Jamestown_Canyon_virus_isolate_ND374_nucleocapsid_protein_and_nonstr C..C..A... .....G..G. ....A.. G......GTG C.C...G.T. ....T..A.. G........A .....T..A. [640]
#KM215559.1_Jamestown_Canyon_virus_isolate_W18393_nucleocapsid_protein_and_nonst C..C...... .....G.... ....A.. G......GTG C.C...G.T. .....T.G.. G.....T... ....A. [640]
#KM215560.1_Jamestown_Canyon_virus_isolate_W18901_nucleocapsid_protein_and_nonst C..C...... .....G.... ....A.. G......GTG C.C...G.T. .....T.G.. G.....T... ....A. [640]
#KX817317.1_Jamestown_Canyon_virus_strain_61V2235_segment_S_complete_sequence C..C...... .....G..G. ....G.. G......GTG C.C...G.T. .....T.G.. G.....C... ....A. [640]

```
#MH370817.1_Jamestown_Canyon_virus_isolate_L36708_segment_S_complete_sequence    C..C...... .....G..G. .......A.. G......GTG C.C...G.C. ....T..A.. G.....T..A ..A.....A.  [640]
#MH370820.1_Jamestown_Canyon_virus_isolate_MN256-260_segment_S_complete_sequence C..C..A... .....G..G. .......A.. G......GTG C.C...G.T. ....T..A.. G........A .....T..A.  [640]
#U12799.1_Jamestown_Canyon_virus_DAV28_S_RNA_segment_N_and_NSs_protein_genes_com C..C...... .....G..G. .......A.. G......GTG C.C...G.T. .....T.G.. G.....C... ........A.  [640]
#U12796.1_Jamestown_Canyon_virus_61v2235_S_RNA_segment_N_and_NSs_protein_genes_c C..C...... .....G..G. .......G.. G......GTG C.C...G.T. .....T.G.. G.....C... ........A.  [640]
#KM215561.1_Jamestown_Canyon_virus_isolate_W23697_nucleocapsid_protein_and_nonst C..C...... .....G..G. .......A.. G......GTG C.C...G.T. ....T..A.. G........A .....T..A.  [640]
#KT288271.1_Inkoo_virus_strain_LEIV-15248Iv_segment_S_nucleoprotein_(N)_gene_com C..C...... .....G.... .......A.. G......GTG C.C...G.T. .A..T..A.. G.....T... .....T..A.  [640]
#KT288274.1_Inkoo_virus_strain_LEIV-18154Yak_segment_S_nucleoprotein_(N)_gene_co C..C...... .....G.... .......A.. G......GTA C.C...G.T. ....T..A.. G.....T... ........A.  [640]
#KT288275.1_Inkoo_virus_strain_LEIV-9874Kar_segment_S_nucleoprotein_(N)_gene_com C..C...... .....G.... .......A.. G......GTG C.C...G.T. .A..T..A.. G.....T... .....T..A.  [640]
#KT288277.1_Inkoo_virus_strain_LEIV-18784Yak_segment_S_nucleoprotein_(N)_gene_co C..C...... .....G.... .......A.. G......GTA C.C...G.T. ....T..A.. G.....T... ........A.  [640]
#KT288280.1_Inkoo_virus_strain_LEIV-22780Tyum_segment_S_nucleoprotein_(N)_gene_c C..C..A... .....G.... .......A.. G......GTG C.C...G.T. ....T..A.. G.....C... ........A.  [640]
#KT288283.1_Inkoo_virus_strain_LEIV-18152Yak_segment_S_nucleoprotein_(N)_gene_co C..C...... .....G.... .......A.. G......GTA C.C...G.T. ....T..A.. G.....T... ........A.  [640]
#KT288286.1_Inkoo_virus_strain_LEIV-21643Kra_segment_S_nucleoprotein_(N)_gene_co C..C...... .....A.... .......A.. G......GTG C.C...G.T. .A.....A.. G.....T... .....T..A.  [640]
#KX554935.1_Inkoo_virus_strain_Lovanger_nucleocapsid_protein_and_nonstructural_p C..C...... .....G.... .......A.. G......GTG C.C...G.T. .A..T..A.. G.....T... .....T..A.  [640]
#U47137.1_Inkoo_virus_Prototype_KN3641_nucleocapsid_protein_and_non-structural_p C..C...... .....G.... .......A.. G......GTG C.C...G.T. .A.....A.. G.....T... .....T..A.  [640]
#U47138.1_Inkoo_virus_SW_AR_83-161_nucleocapsid_protein_and_non-structural_prote C..C...... .....G.... .......A.. G......GTG C.C...G.T. .A..T..A.. G.....T... .....T..A.  [640]
#Z68496.1_Inkoo_virus_RNA_for_N_protein_and_RNA_for_NS_protein_strain_KN_3641    C..C...... .....G.... .......A.. G......GTG C.C...G.T. .A.....A.. G.....T... .....T..A.  [640]
```

```
#KT630290.1_Keystone_virus_strain_KEYV/Ochlerotatus_atlanticus/USA/KEYVLK01/2005 GGGGAAAGGG TGGTCTCAGC GATACTGCCA GAGCTTTTCT TGCTAAATTC GGTGTGAGAC TTCCATGA [708]
#KT630293.1_Keystone_virus_strain_KEYV/Ochlerotatus_atlanticus/USA/KEYVLK02/2005 .......... ...C...... .......... .......C.. .......... ..C....... ........ [708]
#MH016786.1_Keystone_virus_strain_KEYV/Homo_sapiens/Gainesville-1/2016_nucleopro .......... .......... .......... .......... .......... .......... ........ [708]
#KX817323.1_Keystone_virus_strain_B64-5587.05_segment_S_complete_sequence        .......... .......... .......... .......... .......... .......... ........ [708]
#MG821231.1_Keystone_virus_isolate_AR14033_segment_S_complete_sequence           .......A.. .......... .......... ..A....C.. ......G... ..C..C.AG. ........ [708]
#MG765471.1_Keystone_virus_isolate_AVA1709441_nucleocapsid_and_NSs_genes_complet .......A.. .......... .......... ..A....C.. ......G... ..C..C.AG. ........ [708]
#KX817329.1_Melao_virus_strain_TRVL_9375_segment_S_complete_sequence             ....T..... G.....T..T ...G....T. ..A.A..C.. .CAG..G... .....T.... ......A. [708]
#KX817335.1_Serra_do_Navio_virus_strain_BeAr_103645_segment_S_complete_sequence  ......GA.. A...T.G..T ...G...... ..A.A..C.. AT.......T .....C.AG. ......A. [708]
#KX817320.1_Jerry_Slough_virus_strain_BFS_4474_segment_S_complete_sequence       ......GA.. A.....T..T ...G....A. ..A....CT. GATC.....T .....A.A.. .G....A. [708]
#KX817338.1_South_River_virus_strain_NJO-94F_segment_S_complete_sequence         ......GA.. A........T ...G....A. ..A.C..CT. GATC.....T .......A.T .G..T... [708]
#GU018050.2_South_River_virus_isolate_SORV-252_nucleoprotein_and_NSs_protein_gen ......GA.. A......... ...G....A. .GA.C..CT. GAT......T .......A.T .G..T... [708]
#EF681804.1_Jamestown_Canyon_virus_isolate_5592-02_segment_S_nucleocapsid_protei ......GA.. G..G..T... ...G....A. ..A.C..CT. GATC.....T .......A.. .G..T.A. [708]
#EF681805.1_Jamestown_Canyon_virus_isolate_368-99_segment_S_nucleocapsid_protein ......GA.. G..G..T... ...G....A. ..A.C..CT. GATC.....T .......A.. .G..T.A. [708]
#EF681806.1_Jamestown_Canyon_virus_isolate_6163-03_segment_S_nucleocapsid_protei ......GA.. G..G..T... ...G....A. ..A.C..CT. GATC.....T .......A.. .G..T.A. [708]
#EF681807.1_Jamestown_Canyon_virus_isolate_468-04_segment_S_nucleocapsid_protein ......GA.. A..G..T... ...G....A. ..A.A..CT. GATC...... .....A.A.. .G..T... [708]
#EF681808.1_Jamestown_Canyon_virus_isolate_2179-00_segment_S_nucleocapsid_protei ......GA.. G..G..T... ...G....A. ..A.C..CT. GATC.....T .......A.. .G..T.A. [708]
#EF681809.1_Jamestown_Canyon_virus_isolate_779-98_segment_S_nucleocapsid_protein ......GA.. A..G..T... ...G....A. ..A.A..CT. GATC...... .....A.A.. .G..T... [708]
#EF681810.1_Jamestown_Canyon_virus_isolate_810-98_segment_S_nucleocapsid_protein ......GA.. A..G..T... ...G....A. ..A.A..CT. GATC...... .....A.A.. .G..T... [708]
#EF681811.1_Jamestown_Canyon_virus_isolate_811-00_segment_S_nucleocapsid_protein ......GA.. G..G..T... ...G....A. ..A.C..CT. GATC.....T .......A.. .G..T.A. [708]
#EF681812.1_Jamestown_Canyon_virus_isolate_1697-03_segment_S_nucleocapsid_protei ......GA.. A..G..T... ...G....A. ..A.A..CT. GATC...... .....A.A.. .G..T... [708]
#EF681813.1_Jamestown_Canyon_virus_isolate_1425-02_segment_S_nucleocapsid_protei ......GA.. A..G..T... ...G....A. ..A.A..CT. GATC...... .....A.A.. .G..T... [708]
#EF681814.1_Jamestown_Canyon_virus_isolate_1441-04_segment_S_nucleocapsid_protei ......GA.. A..G..T... ...G....A. ..A.A..CT. GATC...... .....A.A.. .G..T... [708]
#EF681815.1_Jamestown_Canyon_virus_isolate_928-00_segment_S_nucleocapsid_protein ......GA.. A..G..T... ...G....A. ..A.A..CT. GATC...... .....A.A.. .G..T... [708]
#EF681816.1_Jamestown_Canyon_virus_isolate_1064-03_segment_S_nucleocapsid_protei ......GA.. A..G.....T ...G....A. ..A.C..C.. GATC.....T .......A.. .G..T.A. [708]
#EF681817.1_Jamestown_Canyon_virus_isolate_1369-02_segment_S_nucleocapsid_protei ......GA.. G..G..T... ...G....A. ..A.C..CT. GATC.....T .......A.. .G..C.A. [708]
#EF681818.1_Jamestown_Canyon_virus_isolate_1627-04_segment_S_nucleocapsid_protei ......GA.. G..G..T... ...G....A. ..A.C..CT. GATC.....T .......A.. .G..T.A. [708]
#EF681819.1_Jamestown_Canyon_virus_isolate_1810-02_segment_S_nucleocapsid_protei ......GA.. G..G..T... ...G....A. ..A.C..CT. GATC.....T .......A.. .G..T.A. [708]
#EF681820.1_Jamestown_Canyon_virus_isolate_2384-98_segment_S_nucleocapsid_protei ......GA.. G..G..T... ...G....A. ..A.C..CT. GATC.....T .......A.. .G..T.A. [708]
#EF681821.1_Jamestown_Canyon_virus_isolate_2707-01_segment_S_nucleocapsid_protei ......GA.. A..G..T... ...G....A. ..A.A..CT. GATC...... .....A.A.. .G..T... [708]
#EF681822.1_Jamestown_Canyon_virus_isolate_2718-01_segment_S_nucleocapsid_protei ......GA.. G..G..T... ...G....A. ..A.C..CT. GATC.....T .......A.. .G..T.A. [708]
#EF681823.1_Jamestown_Canyon_virus_isolate_3280-03_segment_S_nucleocapsid_protei ......GA.. A..G..T... ...G....A. ..A.A..CT. GATC...... .....A.A.. .A..T... [708]
#EF681824.1_Jamestown_Canyon_virus_isolate_3324-04_segment_S_nucleocapsid_protei ......GA.. G..G..T... ...G....A. ..A.C..CT. GATC.....T .......A.. .G..T.A. [708]
#EF681825.1_Jamestown_Canyon_virus_isolate_3573-03_segment_S_nucleocapsid_protei ......GA.. A..G..T... ...G....A. ..A.A..CT. GATC...... .....A.A.. .G..T... [708]
#EF681826.1_Jamestown_Canyon_virus_isolate_3682-00_segment_S_nucleocapsid_protei ......GA.. A..G..T... ...G....A. ..A.A..CT. GATC...... .....A.A.. .G..T... [708]
#EF681827.1_Jamestown_Canyon_virus_isolate_4148-03_segment_S_nucleocapsid_protei ......GA.. A..G.....T ...G....A. ..A.C..C.. GATC.....T .......A.. .G..T.A. [708]
#EF681828.1_Jamestown_Canyon_virus_isolate_4473-00_segment_S_nucleocapsid_protei ......GA.. A..G.....T ...G....A. ..A.C..C.. GATC.....T .......A.. .G..T.A. [708]
#EF681829.1_Jamestown_Canyon_virus_isolate_4742-04_segment_S_nucleocapsid_protei ......GA.. G..G..T... ...G....A. ..A.C..CT. GATC.....T .......A.. .G..T.A. [708]
#EF681830.1_Jamestown_Canyon_virus_isolate_2274-05_segment_S_nucleocapsid_protei ......GA.. G..G..T... ...G....A. ..A.C..C.. GATC.....T .......A.. .G..T.A. [708]
#EF681831.1_Jamestown_Canyon_virus_isolate_1472-05_segment_S_nucleocapsid_protei ......GA.. G..G..T... ...G....A. ..A.C..CT. GATC.....T .......A.. .G..C.A. [708]
#EF681832.1_Jamestown_Canyon_virus_isolate_4910-02_segment_S_nucleocapsid_protei ......GA.. A..G..T... ...G....A. ..A.A..CT. GATC...... .....A.A.. .G..T... [708]
#EF681833.1_Jamestown_Canyon_virus_isolate_275-01_segment_S_nucleocapsid_protein ......GA.. G..G..T... ...G....A. ..A.C..CT. GATC.....T .......A.. .G..T.A. [708]
#EF681834.1_Jamestown_Canyon_virus_isolate_339-05_segment_S_nucleocapsid_protein ......GA.. G..G..T... ...G....A. ..A.C..CT. GATC.....T .......A.. .G..T.A. [708]
#EF681835.1_Jamestown_Canyon_virus_isolate_3836-05_segment_S_nucleocapsid_protei ......GA.. G..G..T... ...G....A. ..A.C..C.. GATC.....T .......A.. .G..T.A. [708]
#EF681836.1_Jamestown_Canyon_virus_isolate_2286-00_segment_S_nucleocapsid_protei ......GA.. A..G..T... ...G....A. ..A.A..CT. GATC...... .....A.A.. .G..T... [708]
#EF681837.1_Jamestown_Canyon_virus_isolate_1044-05_segment_S_nucleocapsid_protei ......GA.. G..G..T... ...G....A. ..A.C..CT. GATC.....T .......A.. .G..T.A. [708]
#EF681838.1_Jamestown_Canyon_virus_isolate_978-99_segment_S_nucleocapsid_protein ......GA.. G..G..T... ...G....A. ..A.C..CT. GATC.....T .......A.. .G..T.A. [708]
#EF681839.1_Jamestown_Canyon_virus_isolate_4832-01_segment_S_nucleocapsid_protei ......GA.. G..G..T... ...G....A. ..A.C..CT. GATC.....T .......A.. .G..T.A. [708]
#EF681841.1_Jamestown_Canyon_virus_isolate_7101-03_segment_S_nucleocapsid_protei ......GA.. G..G..T... ...G....A. ..A.C..C.. GATC.....T .......A.. .G..T.A. [708]
#EF681842.1_Jamestown_Canyon_virus_isolate_Simsbury_segment_S_nucleocapsid_prote ......GA.. A..G..T... ...G....A. ..A.C..CT. GATC.....T .......A.. .G..T.A. [708]
#EF681843.1_Jamestown_Canyon_virus_isolate_8011-03_segment_S_nucleocapsid_protei ......GA.. G..G..T... ...G....A. ..A.C..CT. GATC.....T .......A.. .G..T.A. [708]
#EF681844.1_Jamestown_Canyon_virus_isolate_8536-03_segment_S_nucleocapsid_protei ......GA.. A..G..T... ...G....A. ..A.A..CT. GATC...... .....A.A.. .G..T... [708]
#EF681845.1_Jamestown_Canyon_virus_isolate_11497-03_segment_S_nucleocapsid_prote ......GA.. G..G...... ...G....A. ..A.C..CT. GATC.....T .......A.. .G..T.A. [708]
#EF681846.1_Jamestown_Canyon_virus_isolate_13995-03_segment_S_nucleocapsid_prote ......GA.. G..G..T... ...G....A. ..A.C..C.. GATC.....T .......A.. .G..T.A. [708]
#EF681847.1_Jamestown_Canyon_virus_isolate_1768-98_segment_S_nucleocapsid_protei ......GA.. A..G..T... ...G....A. ..A.A..CT. GATC...... .....A.A.. .G..T... [708]
#EF681848.1_Jamestown_Canyon_virus_isolate_1385-06_segment_S_nucleocapsid_protei ......GA.. G..G..T... ...G....A. ..A.C..CT. GATC.....T .......A.. .G..T.A. [708]
#EF681849.1_Jamestown_Canyon_virus_isolate_2989-06_segment_S_nucleocapsid_protei ......GA.. A..G..T... ...G....A. ..A.C..CT. GATC.....T .......A.. .G..T.A. [708]
```

```
#EF681850.1_Jamestown_Canyon_virus_isolate_3381-06_segment_S_nucleocapsid_protei .....GA.. G..G..T... ...G....A. ..A.C..CT. GATC.....T .......A.. .G..T.A. [708]
#EF681851.1_Jamestown_Canyon_virus_isolate_4095-06_segment_S_nucleocapsid_protei .....GA.. G..G..T... ...G....A. ..A.C..CT. GATC.....T .......A.. .G..T.A. [708]
#EF681852.1_Jamestown_Canyon_virus_isolate_4078-06_segment_S_nucleocapsid_protei .....GA.. G..G..T... ...G....A. ..A.C..CT. GATC.....T .......A.. .G..T.A. [708]
#EF681853.1_Jamestown_Canyon_virus_isolate_11-92_segment_S_nucleocapsid_protein_ .....GA.. A..G..T... ...G....A. ..A.A..CT. GATC...... .....A.A.. .G..T... [708]
#EF681854.1_Jamestown_Canyon_virus_isolate_23-97_segment_S_nucleocapsid_protein_ .....GA.. A..G..T... ...G....A. ..A.A..CT. GATC...... .....A.A.. .G..T... [708]
#EF681855.1_Jamestown_Canyon_virus_isolate_25-97_segment_S_nucleocapsid_protein_ .....GA.. G..G...... ...G....A. ..A.C..CT. GATC.....T .......A.. .G..T.A. [708]
#EF681856.1_Jamestown_Canyon_virus_isolate_29-97_segment_S_nucleocapsid_protein_ .....GA.. A..G..T... ...G....A. ..A.A..CT. GATC...... .....A.A.. .G..T... [708]
#EF681857.1_Jamestown_Canyon_virus_isolate_423-99_segment_S_nucleocapsid_protein .....GA.. G..G..T... ...G....A. ..A.C..CT. GATC.....T .......A.. .G..T.A. [708]
#EF681858.1_Jamestown_Canyon_virus_isolate_1262-98_segment_S_nucleocapsid_protei .....GA.. A..G..T... ...G....A. ..A.A..CT. GATC...... .....A.A.. .G..T... [708]
#EF681859.1_Jamestown_Canyon_virus_isolate_3438-06_segment_S_nucleocapsid_protei .....GA.. G..G..T... ...G....A. ..A.C..CT. GATC.....T .......A.. .G..T.A. [708]
#HM007350.1_Jamestown_Canyon_virus_strain_61V2235_nucleoprotein_and_NSs_protein_ .....GA.. A..A..T..T ...G....A. ..A.C..CT. GATC.....T .....A.AGT .G....A. [708]
#HM007353.1_Jamestown_Canyon_virus_strain_3573-03_nucleoprotein_gene_complete_cd .....GA.. A..G..T... ...G....A. ..A.A..CT. GATC...... .....A.A.. .G..T... [708]
#HM007356.1_Jamestown_Canyon_virus_strain_3324-04_nucleoprotein_and_NSs_protein_ .....GA.. G..G..T... ...G....A. ..A.C..CT. GATC.....T .......A.. .G..T.A. [708]
#KM215518.1_Jamestown_Canyon_virus_isolate_F1819_nucleocapsid_protein_and_nonstr .....GA.. A..A..T..T ...G....A. ..A.C..CT. GATC.....T .......A.T .G..T.A. [708]
#KM215519.1_Jamestown_Canyon_virus_isolate_F6626_nucleocapsid_protein_and_nonstr .....GA.. A..A..T... ...G....A. ..A.C..CT. GATC.....T .......A.T .G..T.A. [708]
#KM215520.1_Jamestown_Canyon_virus_isolate_ND0283_nucleocapsid_protein_and_nonst .....GA.. A..C..T... ...G....A. ..A.C..CT. GATC.....T .......A.T .G..T.A. [708]
#KM215521.1_Jamestown_Canyon_virus_isolate_ND6194_nucleocapsid_protein_and_nonst .....GA.. A..C..T... ...G....A. ..A.C..CT. GATC.....T .......A.T .G..T.A. [708]
#KM215522.1_Jamestown_Canyon_virus_isolate_W6701_nucleocapsid_protein_and_nonstr .....GA.. A..C..T... ...G....A. ..A.C..CT. GATC.....T .......A.T .G..T.A. [708]
#KM215523.1_Jamestown_Canyon_virus_isolate_W14530_nucleocapsid_protein_and_nonst .....GA.. A..C..T... ...G....A. ..A.C..CT. GATC.....T .......A.T .G..T.A. [708]
#KM215524.1_Jamestown_Canyon_virus_isolate_W16690_nucleocapsid_protein_and_nonst .....GA.. A..C..T... ...G....A. ..A.C..CT. GATC.....T .......A.T .G..T.A. [708]
#KM215525.1_Jamestown_Canyon_virus_isolate_W18699_nucleocapsid_protein_and_nonst .....GA.. A.....T..T ...G....A. ..A....C.. GATC.....T .....A.A.. .G....A. [708]
#KM215526.1_Jamestown_Canyon_virus_isolate_W15316_nucleocapsid_protein_and_nonst .....GA.. A..C..T... ...G....A. ..A.C..CT. GATC.....T .......A.T .G..T.A. [708]
#KM215527.1_Jamestown_Canyon_virus_isolate_W17680_nucleocapsid_protein_and_nonst .....GA.. A..C..T... ...G....A. ..A.C..CT. GATC.....T .....A.A.T .G..T.A. [708]
#KM215528.1_Jamestown_Canyon_virus_isolate_W19543_nucleocapsid_protein_and_nonst .....GA.. A..C..T... ...G....A. ..A.C..CT. GATC.....T .......A.T .G..T.A. [708]
#KM215529.1_Jamestown_Canyon_virus_isolate_W19925_nucleocapsid_protein_and_nonst .....GA.. A..C..T... ...G....A. ..A.C..CT. GATC.....T .......A.T .G..T.A. [708]
#KM215530.1_Jamestown_Canyon_virus_isolate_W20764_nucleocapsid_protein_and_nonst .....GA.. A..C..T... ...G....A. ..A.C..CT. AAT......T .....A.A.T .G..T.A. [708]
#KM215531.1_Jamestown_Canyon_virus_isolate_W22352_nucleocapsid_protein_and_nonst .....GA.. A..G..T..T ...G....A. ..A.C..CT. GATC.....T .......A.. .G..T.A. [708]
#KM215532.1_Jamestown_Canyon_virus_isolate_F1829_nucleocapsid_protein_and_nonstr .....GA.. A..A..T..T ...G....A. ..A.C..CT. GATC.....T .......A.T .G..T.A. [708]
#KM215533.1_Jamestown_Canyon_virus_isolate_F6228_nucleocapsid_protein_and_nonstr .....GA.. A..A..T... ...G....A. ..A.C..CT. GATC.....T .......A.T .G..T.A. [708]
#KM215534.1_Jamestown_Canyon_virus_isolate_F6235_nucleocapsid_protein_and_nonstr .....GA.. A..A..T... ...G....A. ..A.C..CT. GATC.....T .......A.T .G..T.A. [708]
#KM215535.1_Jamestown_Canyon_virus_isolate_F10095_nucleocapsid_protein_and_nonst .....GA.. A..C..T... ...G....A. ..A.C..CT. GATC.....T .......A.T .G..T.A. [708]
#KM215536.1_Jamestown_Canyon_virus_isolate_F13418_nucleocapsid_protein_and_nonst .....GA.. A..C..T... ...G....A. ..A.C..CT. GATC.....T .......A.T .G..T.A. [708]
#KM215537.1_Jamestown_Canyon_virus_isolate_F14162_nucleocapsid_protein_and_nonst .....GA.. A..C..T... ...G....A. ..A.C..CT. GATC.....T .......A.T .G..T.A. [708]
#KM215538.1_Jamestown_Canyon_virus_isolate_F14183_nucleocapsid_protein_and_nonst .....GA.. A..C..T... ...G....A. ..A.C..CT. GATC.....T .......A.T .G..T.A. [708]
#KM215539.1_Jamestown_Canyon_virus_isolate_F14278_nucleocapsid_protein_and_nonst .....GA.. A..C..T... ...G....A. ..A.C..CT. GATC.....T .......A.T .G..T.A. [708]
#KM215540.1_Jamestown_Canyon_virus_isolate_F16109_nucleocapsid_protein_and_nonst .....GA.. A..C..T... ...G....A. ..A.C..CT. GATC.....T .......A.T .G..T.A. [708]
#KM215541.1_Jamestown_Canyon_virus_isolate_W8270_nucleocapsid_protein_and_nonstr .....GA.. A..A..T... ...G....A. ..A.C..CT. GATC.....T .......A.T .G..T.A. [708]
#KM215542.1_Jamestown_Canyon_virus_isolate_W16455_nucleocapsid_protein_and_nonst .....GA.. A..C...... ...G....A. ..A.C..CT. GATC.....T .......A.T .G..T.A. [708]
#KM215543.1_Jamestown_Canyon_virus_isolate_W18738_nucleocapsid_protein_and_nonst .....GA.. A..C..T... ...G....A. ..A.C..CT. GATC.....T .......A.T .G..T.A. [708]
#KM215544.1_Jamestown_Canyon_virus_isolate_F4276_nucleocapsid_protein_and_nonstr .....GA.. A..C..T... ...G....A. ..A.C..CT. GATC.....T .......A.T .G..T.A. [708]
#KM215545.1_Jamestown_Canyon_virus_isolate_W19962_nucleocapsid_protein_and_nonst .....GA.. A..C..T... ...G....A. ..A.C..CT. GATC.....T .......A.T .G..T.A. [708]
#KM215546.1_Jamestown_Canyon_virus_isolate_F5609_nucleocapsid_protein_and_nonstr .....GA.. A..A...... ...G....A. ..A.C..CT. GA.C.....T .......A.T .G..T.A. [708]
#KM215547.1_Jamestown_Canyon_virus_isolate_F7826_nucleocapsid_protein_and_nonstr .....GA.. A..A..T... ...G....A. ..A.C..CT. GATC.....T .......A.T .G..T.A. [708]
#KM215548.1_Jamestown_Canyon_virus_isolate_F7932_nucleocapsid_protein_and_nonstr .....GA.. A..C..T... ...G....A. ..A.C..CT. GATC.....T .......A.T .G..T.A. [708]
#KM215549.1_Jamestown_Canyon_virus_isolate_F8532_nucleocapsid_protein_and_nonstr .....GA.. A..A..T... ...G....A. ..A.C..CT. GATC.....T .......A.T .G..T.A. [708]
#KM215550.1_Jamestown_Canyon_virus_isolate_F10720_nucleocapsid_protein_and_nonst .....GA.. A..A..T... ...G....A. ..A.C..CT. GATC.....T .......A.T .G..T.A. [708]
#KM215551.1_Jamestown_Canyon_virus_isolate_ND1300_nucleocapsid_protein_and_nonst .....GA.. A..C..T... ...G....A. ..A.C..CT. GATC.....T .......A.T .G..T.A. [708]
#KM215552.1_Jamestown_Canyon_virus_isolate_W13135_nucleocapsid_protein_and_nonst .....GA.. A..A..T... ...G....A. ..A.C..CT. GATC.....T .......A.T .G..T.A. [708]
#KM215553.1_Jamestown_Canyon_virus_isolate_W16456_nucleocapsid_protein_and_nonst .....GA.. A..C...... ...G....A. ..A.C..CT. GATC.....T .......A.T .G..T.A. [708]
#KM215554.1_Jamestown_Canyon_virus_isolate_W16590_nucleocapsid_protein_and_nonst .....GA.. A..A..T... ...G....A. ..A.C..CT. GATC.....T .......A.T .G..T.A. [708]
#KM215555.1_Jamestown_Canyon_virus_isolate_W19651_nucleocapsid_protein_and_nonst .....GA.. A..C..T... ...G....A. ..A.C..CT. GATC.....T .......A.T .G..T.A. [708]
#KM215556.1_Jamestown_Canyon_virus_isolate_F3406_nucleocapsid_protein_and_nonstr .....GA.. A..C..T... ...G....A. ..A.C..CT. GATC.....T .......A.T .G..T.A. [708]
#KM215557.1_Jamestown_Canyon_virus_isolate_F6577_nucleocapsid_protein_and_nonstr .....GA.. A..C..T... ...G....A. ..A.C..CT. GATC.....T .......A.T .G..T.A. [708]
#KM215558.1_Jamestown_Canyon_virus_isolate_ND374_nucleocapsid_protein_and_nonstr .....GA.. A..C..T... ...G....A. ..A.C..CT. GATC.....T .......A.T .G..T.A. [708]
#KM215559.1_Jamestown_Canyon_virus_isolate_W18393_nucleocapsid_protein_and_nonst .....GA.. A.....T..T ...G....A. ..A....C.. GATC.....T .....A.A.. .G....A. [708]
#KM215560.1_Jamestown_Canyon_virus_isolate_W18901_nucleocapsid_protein_and_nonst .....GA.. A.....T..T ...G....A. ..A....C.. GATC.....T .....A.A.. .G....A. [708]
#KX817317.1_Jamestown_Canyon_virus_strain_61V2235_segment_S_complete_sequence    .....GA.. A..A..T..T ...G....A. ..A.C..CT. GATC.....T .....A.AGT .G....A. [708]
```

```
#MH370817.1_Jamestown_Canyon_virus_isolate_L36708_segment_S_complete_sequence    ......GA.. A..G..T... ...G....A. ..A.A..CT. GATC...... .....A.A.. .G..T... [708]
#MH370820.1_Jamestown_Canyon_virus_isolate_MN256-260_segment_S_complete_sequence ......GA.. A..C..T... ...G....A. ..A.C..CT. GATC.....T .....A.A.T .G..T.A. [708]
#U12799.1_Jamestown_Canyon_virus_DAV28_S_RNA_segment_N_and_NSs_protein_genes_com ......GA.. A..A..T..T ...G....A. ..A.C..CT. GATC.....T .....A.AGT .G....A. [708]
#U12796.1_Jamestown_Canyon_virus_61v2235_S_RNA_segment_N_and_NSs_protein_genes_c ......GA.. A..A..T..T ...G....A. ..A.C..CT. GATC.....T .....A.AGT .G....A. [708]
#KM215561.1_Jamestown_Canyon_virus_isolate_W23697_nucleocapsid_protein_and_nonst ......GA.. A..C..T... ...G....A. ..A.C..C.. GATC..G..T .......A.T .G..T.A. [708]
#KT288271.1_Inkoo_virus_strain_LEIV-15248Iv_segment_S_nucleoprotein_(N)_gene_com ....G.GA.. A..A..T..T ...G....A. ..A.C..C.. GATC.....T .....A.A.. .G..T.A. [708]
#KT288274.1_Inkoo_virus_strain_LEIV-18154Yak_segment_S_nucleoprotein_(N)_gene_co ......GA.. A..A..T... ...G....A. ..A.C..CT. GATC.....T .......A.. .G..T.A. [708]
#KT288275.1_Inkoo_virus_strain_LEIV-9874Kar_segment_S_nucleoprotein_(N)_gene_com ....G.GA.. A..A..T..T ...G....A. ..A.C..C.. GATC.....T .....A.A.. .G..T.A. [708]
#KT288277.1_Inkoo_virus_strain_LEIV-18784Yak_segment_S_nucleoprotein_(N)_gene_co ......GA.. A..A..T... ...G....A. ..A.C..CT. GATC.....T .......A.. .G..T.A. [708]
#KT288280.1_Inkoo_virus_strain_LEIV-22780Tyum_segment_S_nucleoprotein_(N)_gene_c ......GA.. A..A..T... ...G....A. ..A.C..CT. GATC.....T .......A.. .G..T.A. [708]
#KT288283.1_Inkoo_virus_strain_LEIV-18152Yak_segment_S_nucleoprotein_(N)_gene_co ......GA.. A..A..T... ...G....A. ..A.C..CT. GATC.....T .......A.. .G..T.A. [708]
#KT288286.1_Inkoo_virus_strain_LEIV-21643Kra_segment_S_nucleoprotein_(N)_gene_co ......GA.. A..A..T... ...G....A. ..A....CT. GATC.....T .....A.A.. .G..T.A. [708]
#KX554935.1_Inkoo_virus_strain_Lovanger_nucleocapsid_protein_and_nonstructural_p ....G.GA.. A..A..T..T ...G....A. ..A.C..C.. GATC.....T .....A.A.. .G..T.A. [708]
#U47137.1_Inkoo_virus_Prototype_KN3641_nucleocapsid_protein_and_non-structural_p ......GA.. A..A..T..T ...G....A. ..A....CT. GATC.....T .....A.AG. .G..T.A. [708]
#U47138.1_Inkoo_virus_SW_AR_83-161_nucleocapsid_protein_and_non-structural_prote ....G.GA.. A..A..T..T ...G....A. ..A.C..C.. GATC.....T .....A.A.. .G..T.A. [708]
#Z68496.1_Inkoo_virus_RNA_for_N_protein_and_RNA_for_NS_protein_strain_KN_3641    ......GA.. A..A..T..T ...G....A. ..A....CT. GATC.....T .....A.AG. .G..T.A. [708]
```

Supplemental Document 3: merged fragments reassortment analyses. Segments L and M were merged, separated by 10 "Ns" and scanned with RDP4 algorithms. The breakpoints identified by RDP4 match the boundaries of the segments.

Breakpoint Positions relative to B64-5587.05 (segments L+M)				
**Start**	**End**	**Reassorting Sequence(s)**	**Minor Parental Sequence(s)**	**Major Parental Sequence(s)**
6958	11440	^B64-5587.05_segLM	AVA1709441_segLM	Unknown (StJohnsCounty-FL3_2019_segLM)
		KEYVLK01_2005_segLM	AR14033_segLM	Unknown(StJohnsCounty-FL2_2019_segLM)
		KEYVLK02_2005_segLM	StJohnsCounty-FL_2019_segLM	Unknown(StJohnsCounty-FL4_2019_segLM)
		Gainesville-1_2016_segLM		Unknown(StJohnsCounty-FL5_2019_segLM)

Table key:
^ = The reassorted sequence may have been misidentified (one of the identified parents might be the reassorted)
Minor Parent = Parent contributing the smaller fraction of sequence.
Major Parent = Parent contributing the larger fraction of sequence.
Unknown = Only one parent and a reassorted need be in the alignment for a reassortment event to be detectable. The sequence listed as unknown was used to infer the existence of a missing parental sequence.
